# Construction of mate pair full-length cDNAs libraries and characterization of transcriptional start sites and termination sites

**DOI:** 10.1093/nar/gku600

**Published:** 2014-07-17

**Authors:** Kyoko Matsumoto, Ayako Suzuki, Hiroyuki Wakaguri, Sumio Sugano, Yutaka Suzuki

**Affiliations:** Graduate School of Frontier Sciences, University of Tokyo, 5-1-5 Kashiwanoha, Kashiwa, Chiba 277-8562, Japan

## Abstract

To identify and characterize transcript structures ranging from transcriptional start sites (TSSs) to poly(A)-addition sites (PASs), we constructed and analyzed human TSS/PAS mate pair full-length cDNA libraries from 14 tissue types and four cell lines. The collected information enabled us to define TSS cluster (TSC) and PAS cluster (PAC) relationships for a total of 8530/9400 RefSeq genes, as well as 4251/5618 of their putative alternative promoters/terminators and 4619/4605 intervening transcripts, respectively. Analyses of the putative alternative TSCs and alternative PACs revealed that their selection appeared to be mostly independent, with rare exceptions. In those exceptional cases, pairs of transcript units rarely overlapped one another and were occasionally separated by Rad21/CTCF. We also identified a total of 172 similar cases in which TSCs and PACs spanned adjacent but distinct genes. In these cases, different transcripts may utilize different functional units of a particular gene or of adjacent genes. This approach was also useful for identifying fusion gene transcripts in cancerous cells. Furthermore, we could construct cDNA libraries in which 3′-end mate pairs were distributed randomly over the transcripts. These libraries were useful for assembling the internal structure of previously uncharacterized alternative promoter products, as well as intervening transcripts.

## INTRODUCTION

To define gene regions in the genome and to identify the exact structures of their encoding transcripts, it is essential to know the exact transcriptional start site (TSS) and poly(A)-addition site (PAS). Conversely, the term gene itself and the modular architecture of genes and genomes could be defined by TSSs and PASs (1). Indeed, in certain extreme cases, ribonucleic acids (RNAs) are transcribed from components that overlap two genes; thus, the definitions of genes are not always straightforward (2). Accurate positional information on TSSs has been collected in a genome-wide manner by intensive analyses of the so-called full-length complementary deoxyribonucleic acids (cDNAs) using cap structure trapping technologies, such as oligo capping (3,4) and cap analysis of gene expression (5–7). Information on PASs has also been accumulated mainly using the 3′-end information of expressed sequence tags (ESTs) (8), followed recently by intensive RNA Seq analysis (9,10). More recently, the so-called PA Seq method has also been developed to detect PAS sites (11).

In spite of intensive efforts, for a number of genes, especially for a number of intervening long non-protein coding RNAs (lncRNAs) (12–16), it remains elusive which TSSs and PASs should be paired, thus enabling us to define the transcript regions between them. There is also a concern that although the ENCODE (17–19) or modENCODE (20) project has generated genome-wide catalogs of transcripts using primarily RNA Seq in a wide variety of cell types from various species, the sequence depth for each data set might still be inadequate to cover rare transcripts, such as lncRNAs. Although the transcript regions are sufficiently covered, it is often difficult to define gene boundaries based solely on the tag information derived from fragmented transcripts. Indeed, this lack of precise structure of the transcripts imposes serious problems on associating RNA Seq tags with particular transcript units to calculate their expression levels. Moreover, it is unclear whether there is any association between TSSs and PASs. There is also a discussion regarding potential alternative promoters and whether their transcripts have protein-coding potential or represent non-protein coding short RNAs, such as promoter-associated short RNAs (21). It is difficult to distinguish these two possibilities solely using RNA Seq.

In this study, we constructed and analyzed TSS/PAS mate pair full-length cDNA libraries (TSS/PAS library) in which the TSS and PAS originated from a single messenger RNA (mRNA) were connected. These connected tags could be analyzed on the next-generation sequencing platforms. For this purpose, we circularized the mRNAs and tagged the 5′- and 3′-ends with a cap-replacing oligo and a dT adaptor primer, respectively. Ni *et al.* (22) reported the construction of a mate pair library using a similar method. However, their methodology was described only in a model cell system in flies and has not been practically applied to any other organisms, including humans. Furthermore, as was reported in an initial method paper, to our knowledge their method has not been used for data production to actually analyze transcript structure. Ruan *et al.* also reported a similar method, RNA-PET, in which TSS clusters (TSCs) and PAS clusters (PACs) are ligated and analyzed (23). However, their method requires cloning of the full-length cDNAs to the plasmid vector first; thus, delicate handling of the materials in the long procedures is necessary. Here, we propose a much simpler protocol to achieve a similar analysis. In addition, we also developed a new procedure to construct TSS/Random libraries, in which the 3′-ends of the mRNAs were tagged with random hexamer primers instead of dT adaptor primers. By using TSS/Random libraries, we also expected to be able to analyze the relationship between TSSs and internal exons.

**Figure 1.**
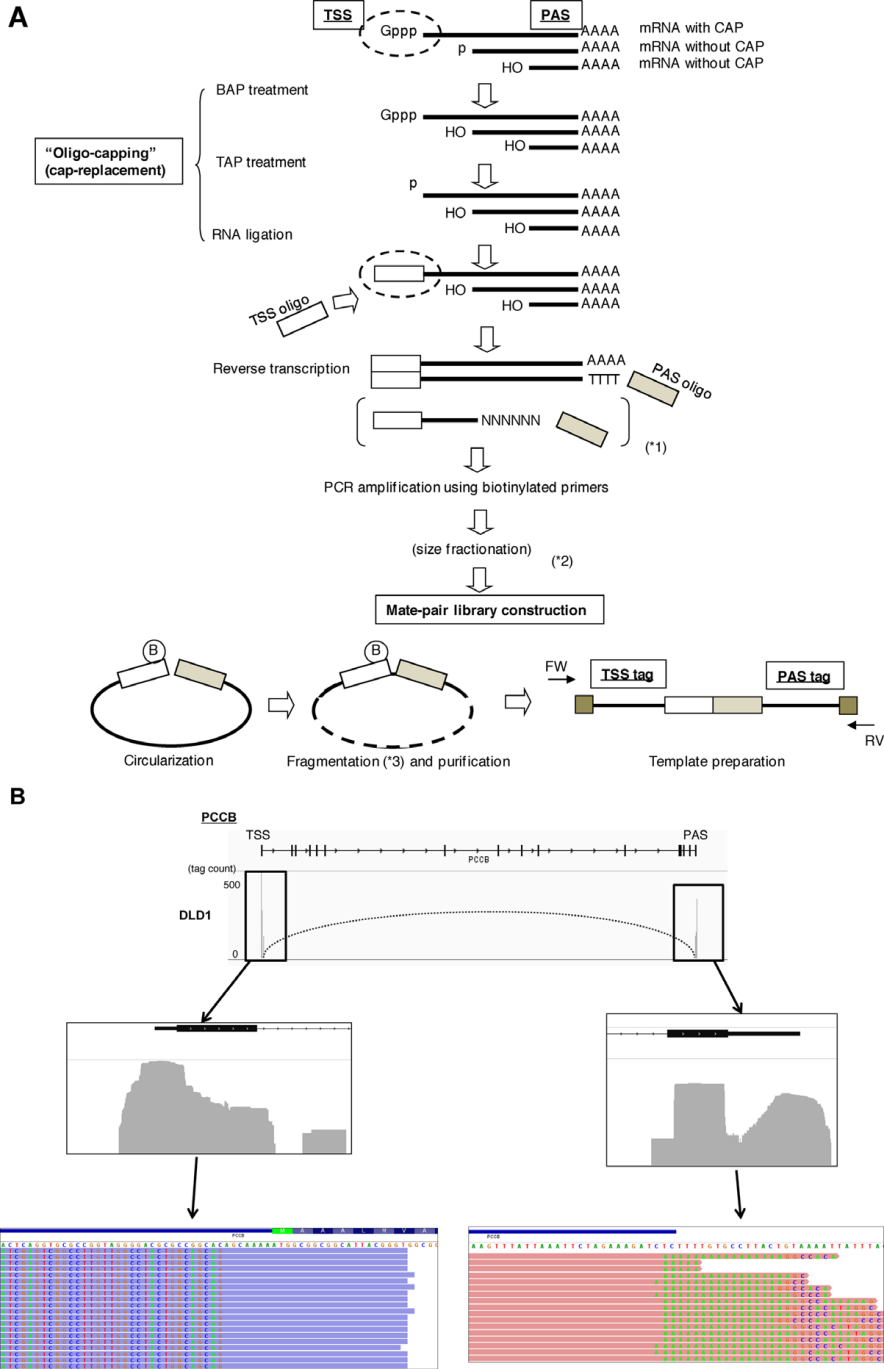
Construction and characterization of the mate pair full-length cDNA libraries. (**A**) Schematic representation of the mate pair full-length cDNA library construction. Briefly, the cap structure of RNA was replaced with a synthetic oligo-ribonucleotide by BAP-TAP-RNA ligase treatment. Full-length cDNAs with adaptor sites at both ends were amplified by PCR. Biotinylated-oligo was used for the 5′-end PCR primer. The PCR amplicons were circularized and fragmented. Subsequently, the fragments with biotin were recovered by avidin column. Non-circularized fragments were degraded by exonuclease treatment. For the purified ‘TSS-PAS’ mate pair fragments, sequence adaptors were ligated to the both ends, and templates for the next-generation sequencing were generated. Details of the protocols are described in the Materials and Methods section and Supplementary Methods as well as on our web site: http://dbtss.hgc.jp/cgi-bin/protocol_matepairLibrary.cgi. (**B**) Examples of TSCs and PACs identified in the TSS/PAS libraries. The case of the *PCCB* gene is shown. The peaks indicate the tag counts from the TSS/PAS mate pair libraries (upper). The numbers of tag counts are shown in the margin. The dotted curve indicates the TSC-PAC pair. The cell type from which the TSC/PAC was derived is shown in the left margin. Magnifications of the upper panels are shown in the middle panels for the indicated regions. Lower panels show the tag sequences. Bases derived from adaptor or poly(A) sequences, which were thus mismatched to the reference genome, are highlighted.

**Figure 2.**
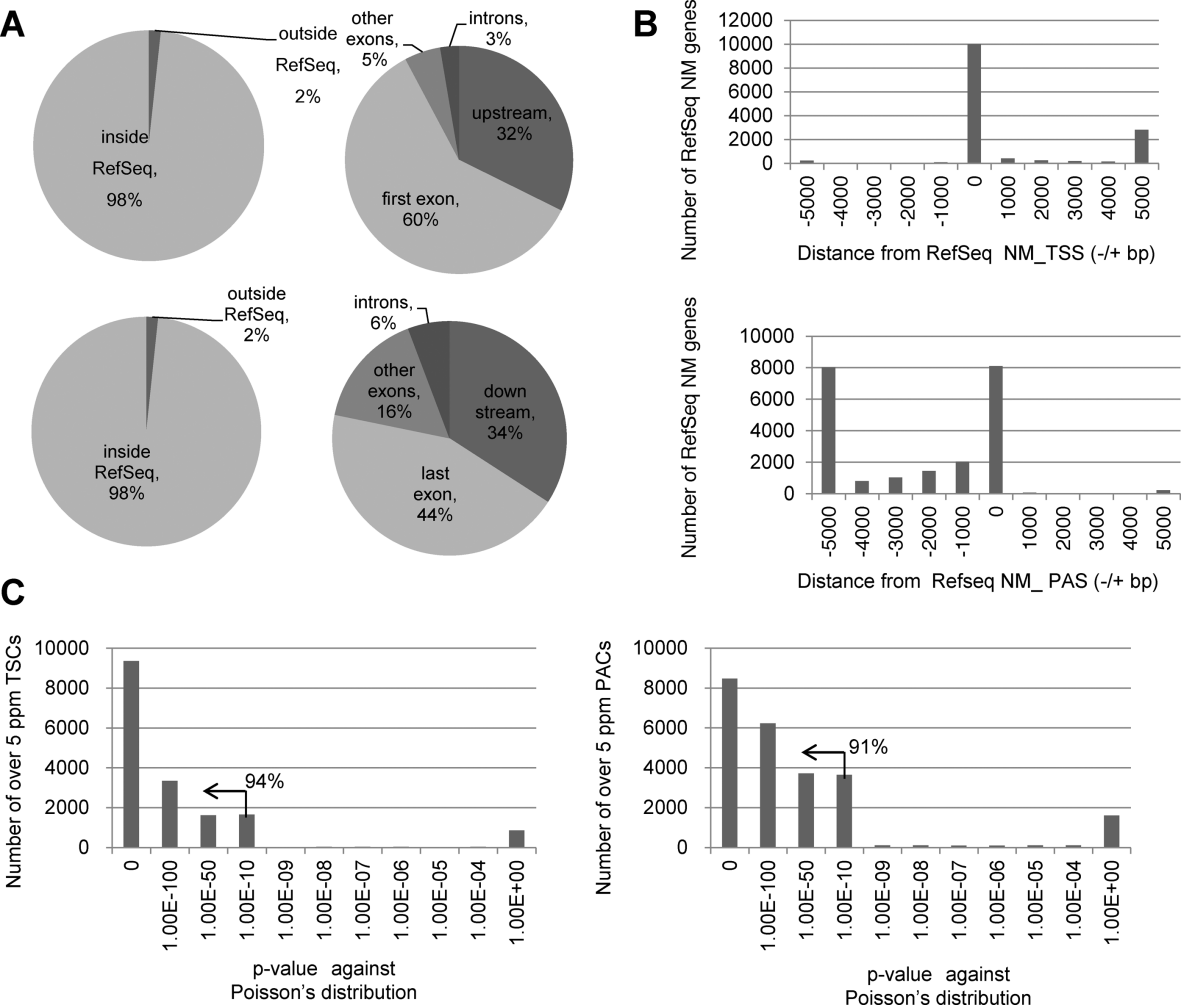
Evaluation of the TSS/PAS libraries. (**A**) Positions of the TSS/PAS mate pair tags relative to RefSeq genes. The frequencies of the TSS/PAS tags were calculated depending on their location within or outside RefSeq gene regions (left panels). Among the tags associated with RefSeq genes, their distributions were further separated depending on the internal positions of the RefSeq transcript models (right panels). The top panels show the TSS tags and the bottom panels show the PAS tags. The right panels represent the breakdowns of the population ‘inside RefSeq’ in the left panels. (**B**) The distribution of the locations of TSS tags and PAS tags relative to the RefSeq NM transcript model. The top panel shows the TSS tags and the bottom panel shows the PAS tags (see Supplementary Figure S2J for further breakdowns of the longer populations). Additionally, note that in many cases, the RefSeq model included a long transcript with a distal 5′-exon. At the same time, RefSeq annotates another 5′-exon, which overlaps with our TSC, downstream from that distal 5′-exon (right margin; Supplementary Figure S2J). For further details on the overlap between the TSCs or our data and the RefSeq data, see Supplementary Figure S2A–C. (**C**) Statistical significance of the biased distribution of the TSS tags and PAS tags to the TSC or PAC (left and right panels, respectively) calculated against the random distribution on the mRNA assuming a Poisson distribution. The numbers of TSCs or PACs giving the indicated *P* values (x-axis) are shown. The percentages in the plots show the proportions of the indicated populations (*P* < 1e−10).

**Figure 3.**
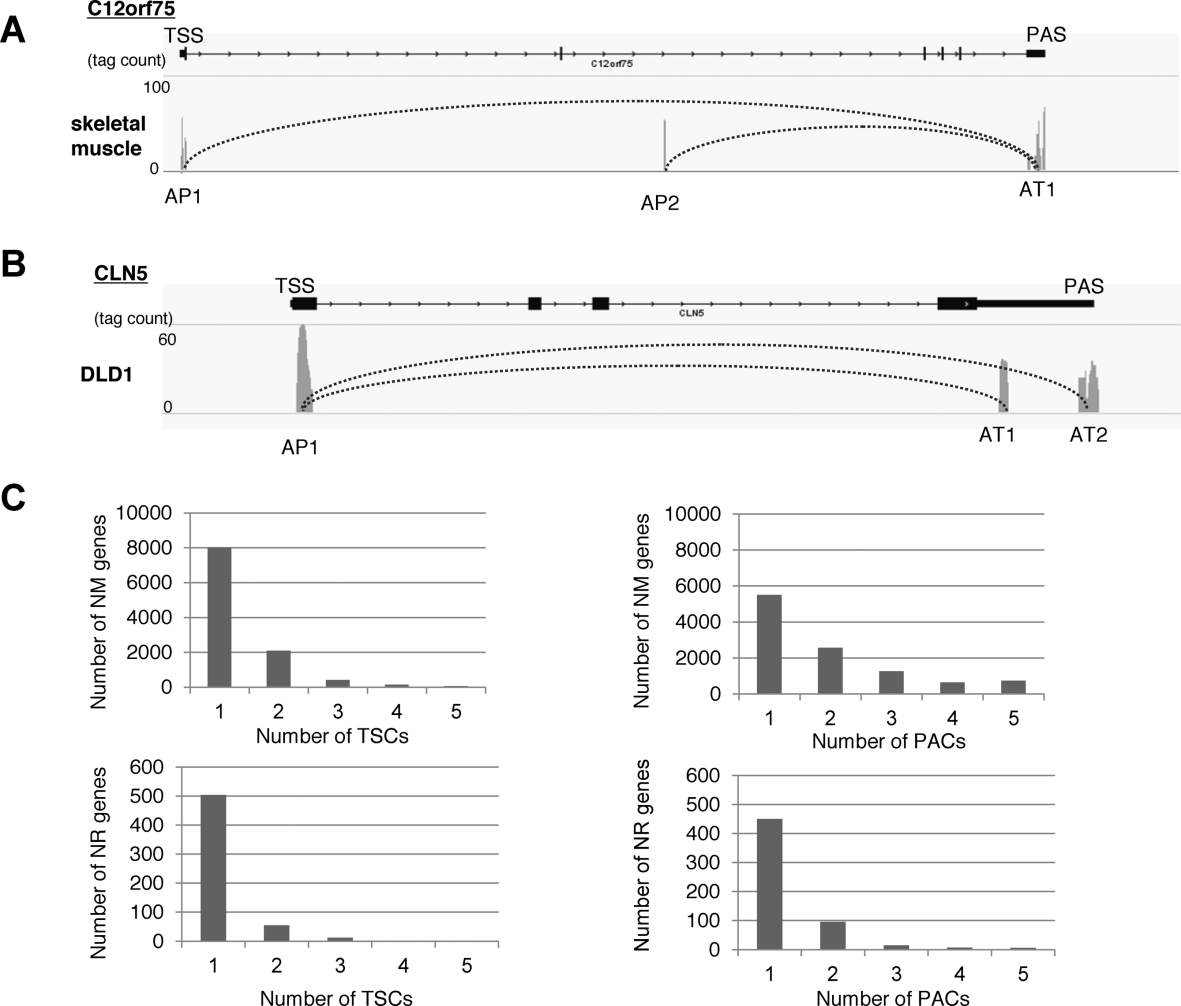
Characterization of multiple TSCs and PACs within a single gene. (**A, B**) Examples of genes in which multiple TSCs and PACs were observed. The peaks indicate the tag counts from the TSS/PAS mate-pair library (MPL), and the curve indicates the TSC-PAC pair. Each peak represents the cluster of TSCs or PACs. AP: alternative promoter region; AT: alternative termination site. (**C**) The number of RefSeq NM (protein-coding genes; top panels) and NR (putative lncRNAs; bottom panels) genes in which multiple TSCs (left panel) and PACs (right panel) were identified.

**Figure 4.**
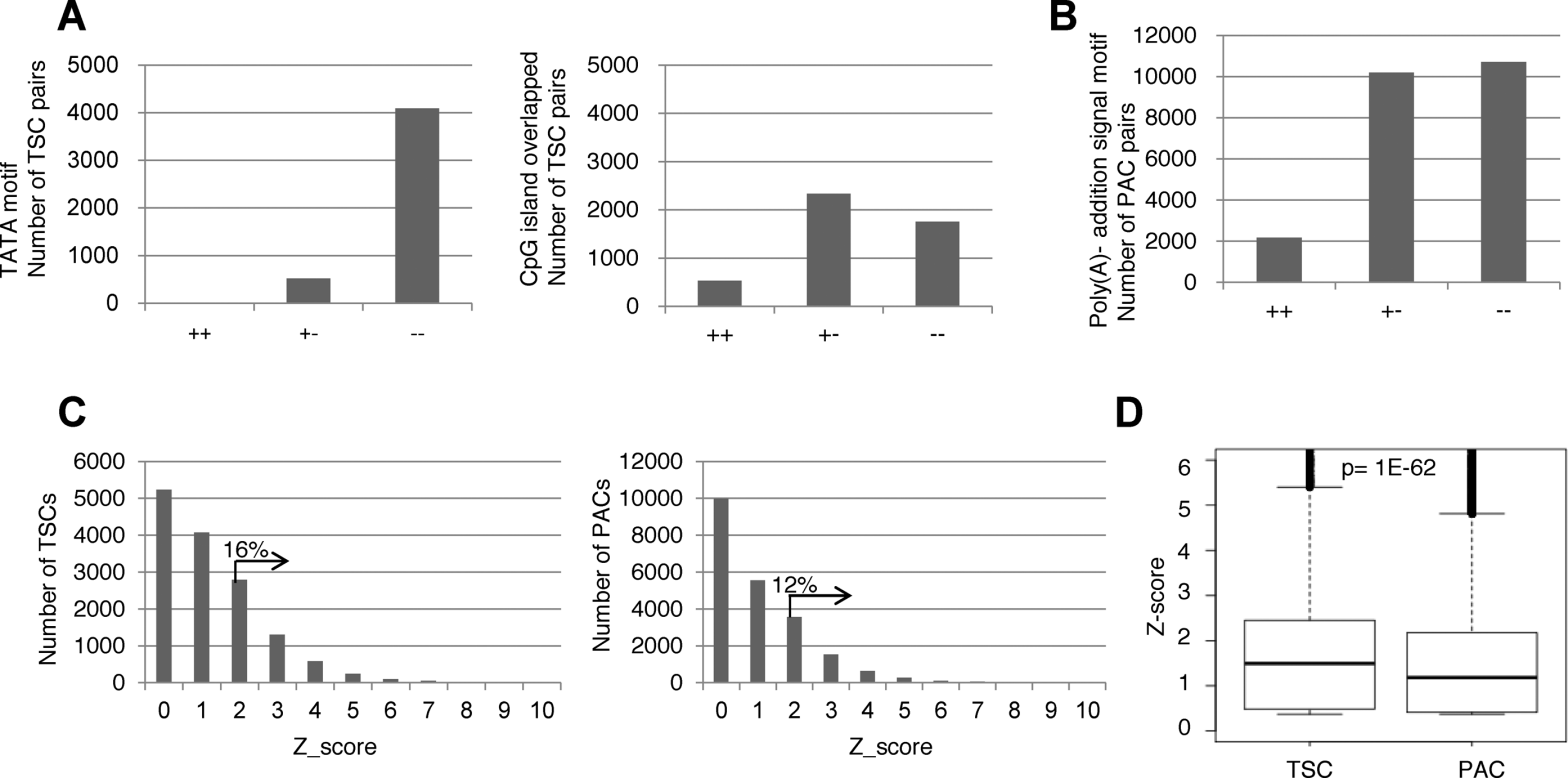
Characterization of multiple TSCs and PACs. (**A**) The presence of potential *cis*-regulatory motifs between multiple TSCs and PACs. The number of alternative TSCs for which TATA boxes (left panel) or CpG islands (right panel) were detected for both (+ +), either (+ −) or neither (− −) of the TSCs. (**B**) Results of a similar analysis as in (A) for the alternative PACs and the presence of the canonical poly(A)-addition sequence. For the definitions of each *cis*-motif, see the Materials and Methods section. (**C**) Tissue specificities of the detected TSCs (left panel) and PACs (right panel). The Z-scores in the plots were calculated for each TSC or PAC according to the procedure described in the Materials and Methods section. The percentages represent the proportions belonging to the indicated populations. (**D**) Boxplots showing the distributions of the Z-scores for the TSCs and PACs. The statistical significance of the difference, as evaluated by Wilcoxon's signed rank test, is shown at the top.

**Figure 5.**
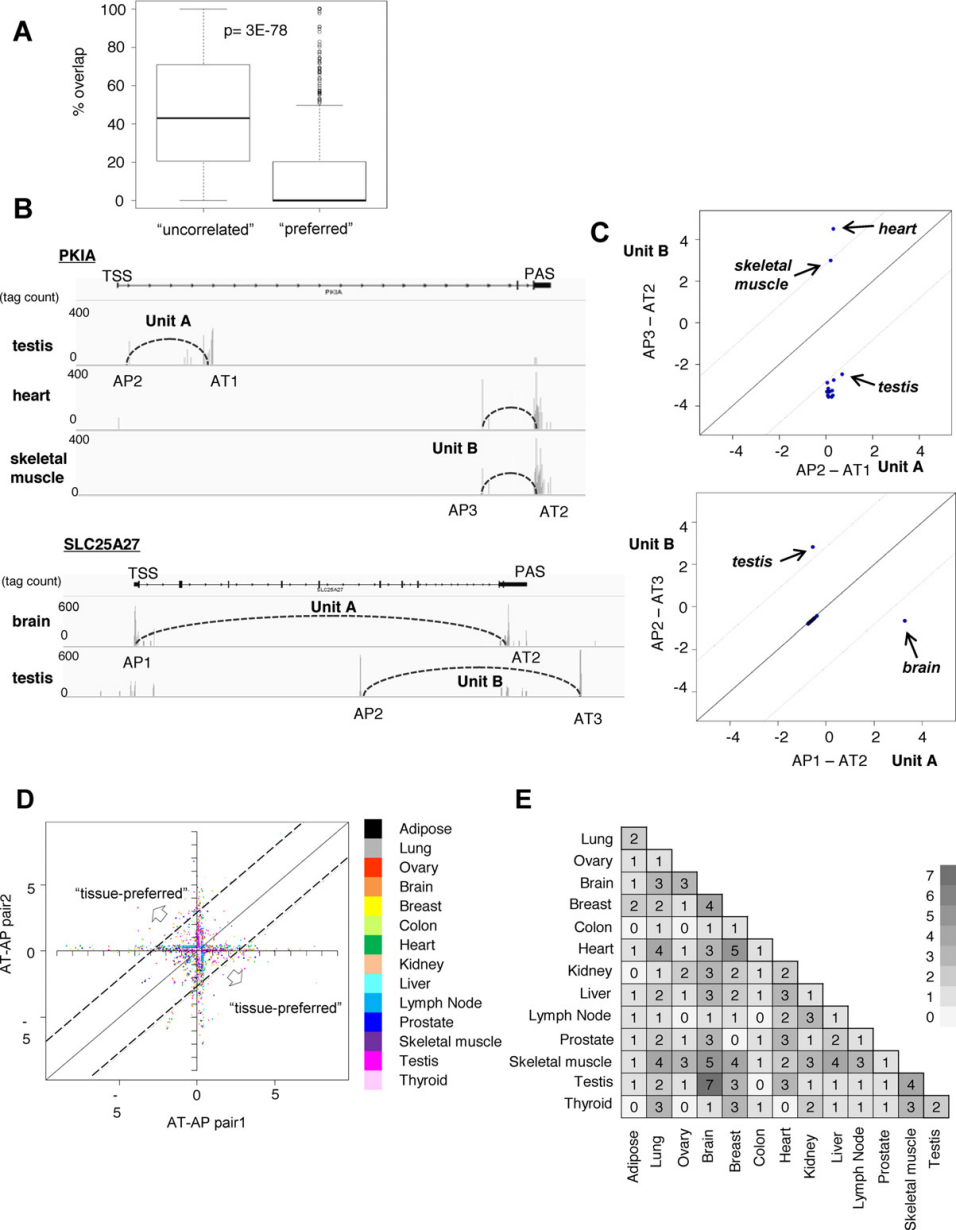
Characterization of mutually preferred TSC-PAC pairs. (**A**) Overlap of the protein-coding regions of two pairs of TSC-PACs within a single gene. A boxplot chart of the proportion of the genic regions that overlapped between pairs of TSC-PACs is shown. The left entry represents the total population and the right entry represents the ‘preferred’ TSC-PACs. The statistical significance of the difference was evaluated by Wilcoxon's signed rank test. (**B**) Examples of multiple TSCs and PACs for which mutual selections were preferred over other pairs. The cases of the *PKIA* and *SLC25A27* genes are shown. Expression profiles of the indicated TSC–PAC relationships are also shown in the indicated tissues. Vertical lines indicate the respective tag counts. (**C**) Expression patterns of the ‘preferred’ TSC–PAC relationships. For each ‘preferred’ TSC-PAC pair (Units A and B), a Z-score of the expression pattern was calculated for each unit (Unit A on the x-axis and Unit B on the y-axis) in the indicated tissues. (**D**) Overlay of a plot similar to the one in (C) with the plot of the total ‘preferred’ TSC-PACs. Color code for the tissues is indicated in the inset. (**E**) The number of ‘preferred’ TSC–PAC relationships for which the expression patterns were switched between the indicated tissues with Z-scores of >2.

**Figure 6.**
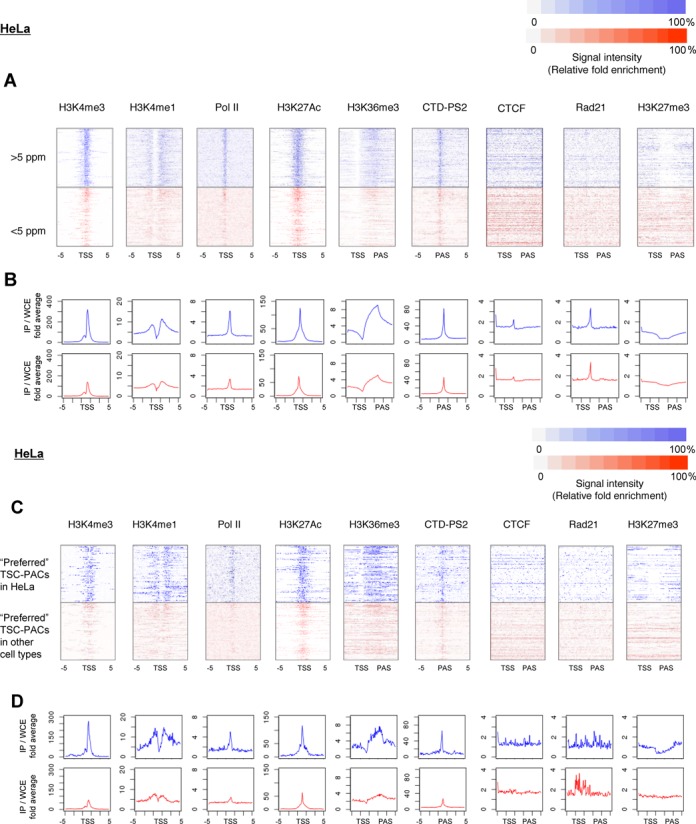
Relationships between transcript structure and chromatin signature. (**A**) Densities of ChIP Seq signals in HeLa cells for the indicated chromatin signatures. Color code of the ChIP Seq tag density is shown in the margin. Cases where the transcript levels are >5 ppm (upper) and <5 ppm (lower) are shown in blue and red, respectively. For Pol II, H3K4me1, H3K4me3 and H3K27Ac, peaks within 5 kb from the 5′-end of the RefSeq genes were used for the centralization. For H3K36me3, H3K27me3, Rad21 and CTCF, the transcript region defined as the region between TSC and PAC was normalized, and the ChIP Seq signals were plotted accordingly. For CTD-PS2, the peaks located within the range of −5 kb to 5 kb from the PACs were selected and centralized to the center of the peaks. (**B**) Average values of the ChIP Seq signal intensities at the indicated positions (x-axis). (**C, D**) Results of similar analysis to (A) and (B) for the preferred TSC-PAC regions.

**Figure 7.**
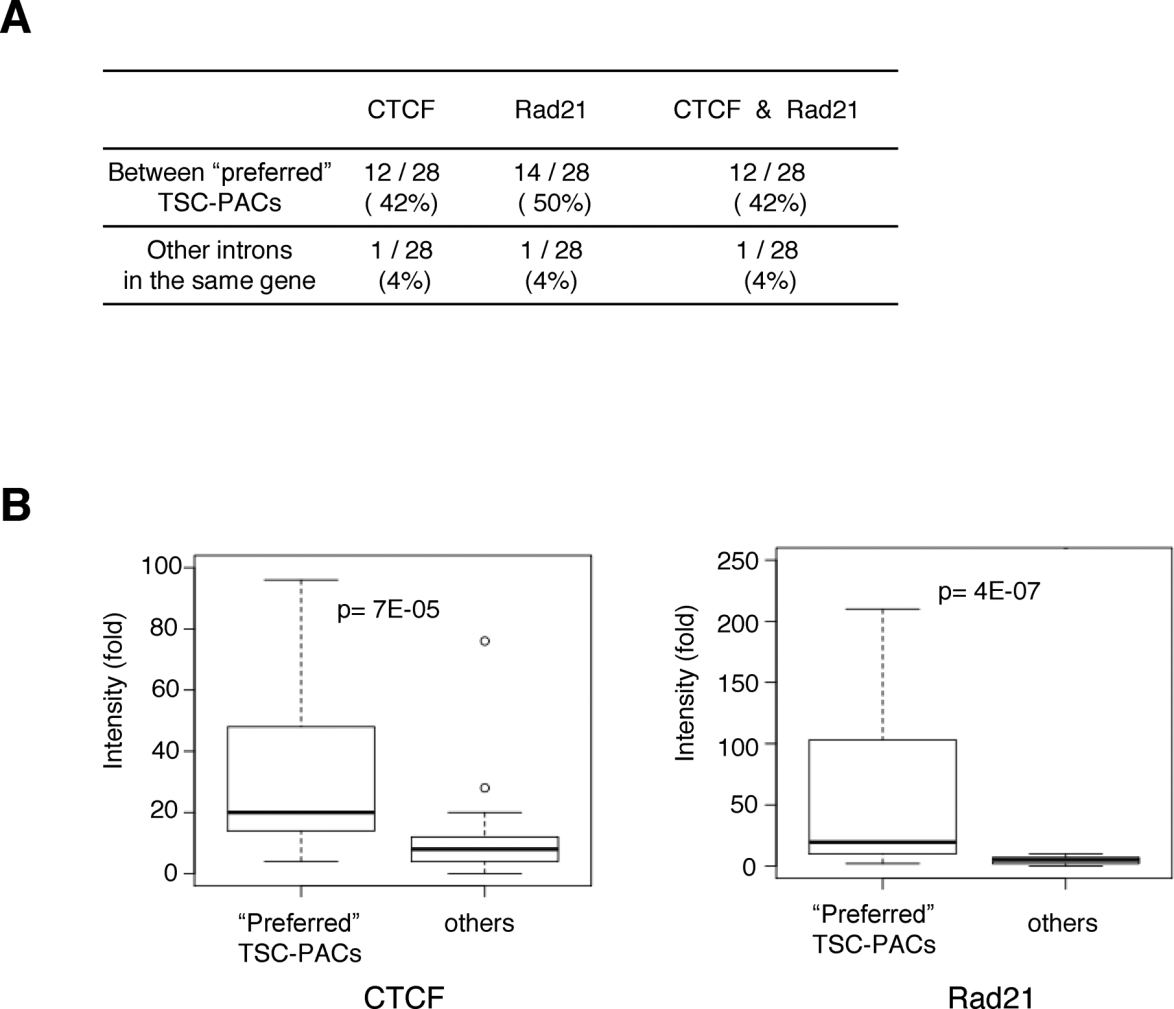
(**A**) Chromatin signatures between preferred TSC-PAC pairs. Frequencies of the ChIP Seq peaks for Rad21 and CTCF identified in the indicated regions. (**B**) Densities of the ChIP Seq signals for the indicated populations. The statistical significances of the differences, as evaluated by Wilcoxon's signed rank test, are shown at the top.

**Figure 8.**
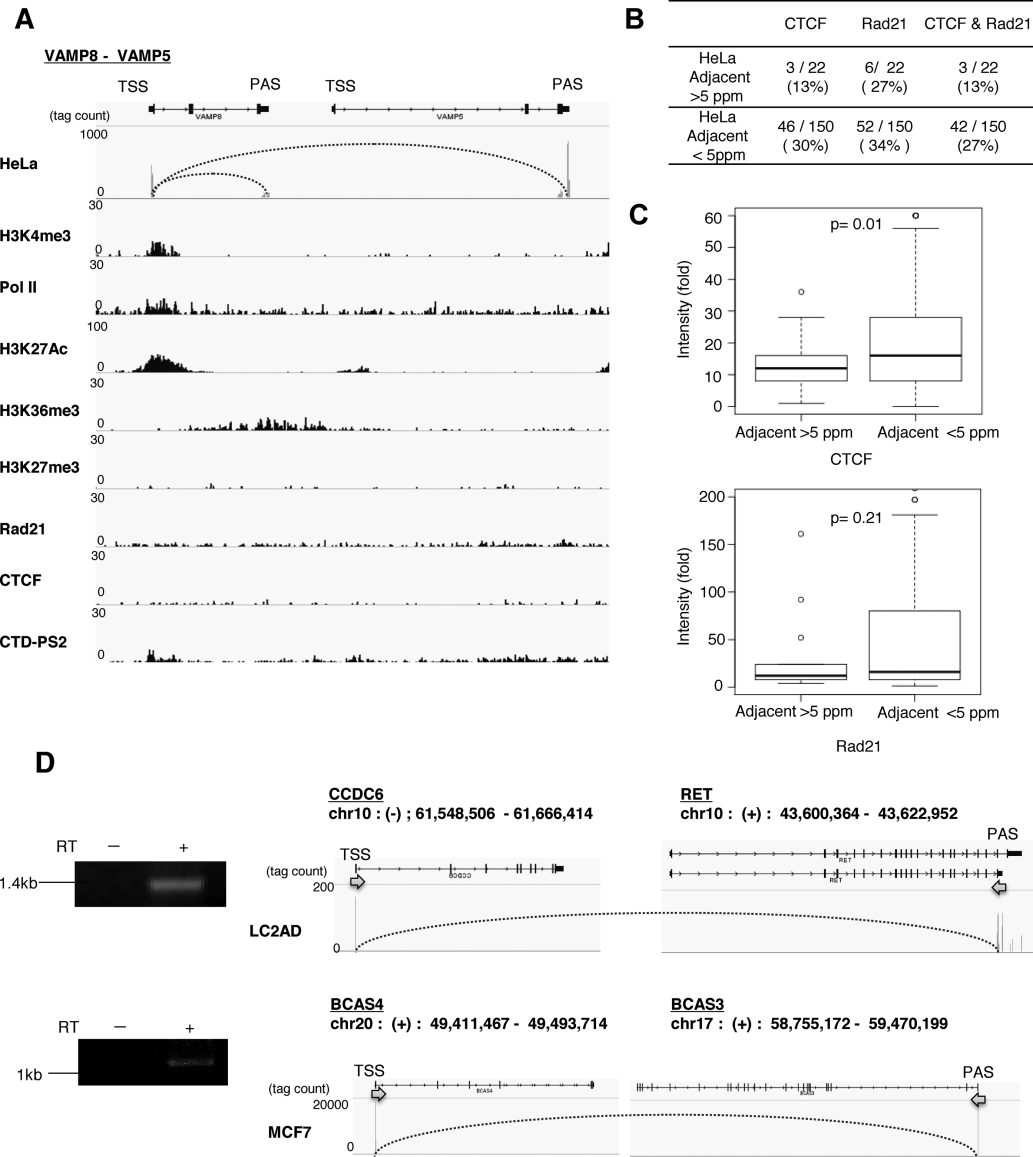
Use of the TSS/PAS library to analyze diverse transcript regions. (**A**) Examples of genomic regions for which the TSC-PAC pairs ‘connected’ adjacent RefSeq genes. Patterns of TSS-PAS tags (from the HeLa dT library) and ChIP Seq patterns are shown. Antibodies used for ChIP Seq are shown in the left margin. For ChIP Seq tracks, a unified scale was used for the y-axis. (**B**) Frequencies of ChIP Seq peaks (*P* < 1e−5 as of MACS) for Rad21 and CTCF among the ‘connected’ RefSeq genes. Cases where ChIP Seq peaks were identified in the regions between the corresponding RefSeq regions were counted among the total cases of ‘connected’ RefSeq genes. Populations were counted separately for cases having TSC-PAC pairs of >5 ppm (upper) and <5 ppm (lower) in HeLa cells. (**C**) Boxplot of the densities of the ChIP Seq signals (fold of normalized tag counts divided by normalized background input) for the indicated populations [‘connected’ genes having TSC-PACs of >5 ppm (left) and <5 ppm (right)]. The antibody used for the ChIP Seq analysis is shown at the bottom. The statistical significance of the difference, as evaluated by Wilcoxon's signed rank test, is shown at the top. (**D**) Fusion gene transcripts in cancerous cells identified in this study. Graphical views are shown for the cases of the *BCAS4*-*BCAS3* gene in MCF7 cells (top) and the *CCDC6*-*RET* gene in LC2AD cells (bottom). RT-PCR validations of the identified fusion gene transcripts are shown in the left margin.

We constructed and analyzed TSS-PAS/Random mate pair full-length cDNA libraries from 14 human tissues and four human cell lines. We demonstrated that this approach is useful for analyzing transcript regions for putative alternative promoter products, lncRNAs, and genomic regions from which diverse transcripts are transcribed. We also demonstrate that this method is useful for detecting fusion gene products in cancerous cells. Briefly, in this paper, we describe the methodology and the initial data descriptions in the first section (Figures 1–3 and 6; Tables 1 and2). Then, we describe how we applied the obtained data for the analyses of transcript structures (Figures 4, 5, 7 and 8). Details of the protocol are also shown in Supplementary Methods and on our web site (http://dbtss.hgc.jp/cgi-bin/protocol_matepairLibrary.cgi).

## MATERIALS AND METHODS

### Data set generated and used for the analysis

The sequence data appearing in the present study were deposited to the DDBJ under the accession numbers DRA001232. The graphical views for individual cases are also implemented in our database, DBTSS (http://dbtss.hgc.jp/). A part of the chromatin immunoprecipitation (ChIP) Seq data is also shared with our previous publication (24).

### RNA materials

Total RNAs from normal human tissues were purchased from companies as follows: adipose (Ambion AM7956), brain (Ambion AM7962), breast (Bio Chain R1234086-50), colon (Bio Chain R1234090-50), heart (Ambion AM7966), kidney (Ambion AM7976), liver (Ambion AM7960), lung (Ambion AM7968), lymph node (Ambion AM7894), ovary (Ambion AM6974), prostate (Ambion AM7988), skeletal muscle (Ambion AM7982), testis (Ambion AM7972) and thyroid (Ambion AM6872).

HeLa cells (ATCC #CCL-2) and HEK293 cells (ATCC #CRL-1573) were cultured in 15-cm dishes in Dulbecco's modified Eagle's medium (DMEM) supplemented with 10% fetal bovine serum and kanamycin. DLD1 cells (ATCC #CCL-221) were cultivated in 15-cm dishes in DMEM with 4.5-g/l D-glucose supplemented with 10% fetal bovine serum and kanamycin. MCF7 (ATCC #HTB-22) cells were cultivated in 15-cm dishes in MEM without phenol red supplemented with 10% fetal bovine serum and kanamycin. Total RNAs were extracted with RNeasy (QIAGEN). The quality and quantity of the total RNAs were inspected with BioAnalyzer (Agilent).

### Construction of mate pair libraries of full-length cDNAs

- *Adaptor ligation of cap-replacing oligo.* For each sample, 100-μg total RNA was used for the library construction. The cap-replacing and oligo-capping procedures were performed as previously described (3,4). Briefly, RNA was treated with 2.5-U bacterial alkaline phosphatase (BAP) (TaKaRa) at 37°C for 1 h and 40-U tobacco acid pyrophosphatase (TAP) (Ambion) at 37°C for 1 h. The treated RNA was then cap-replaced using the 5′-oligoribonucleotide: 5′-AGCAUCGAGUCGGCCUUGUUGGCCUACUGGCAGCAG-3′ (100 ng/μl; custom order, TaKaRa) for the RNA ligation, with 250-U T4 RNA ligase (TaKaRa) at 20°C for 3 h. The adaptor-ligated RNA was treated with 10-U DNaseI (TaKaRa) at 37°C for 10 min. Poly(A)-containing RNA was selected with oligo-dT powder (Cosmo Bio). We synthesized first strand DNA using 400-U Super ScriptII (Invitrogen) at 12°C for 1 h and at 42°C overnight with the oligo-dT adaptor primer: 5′-GCGGCTGAAGACGGCCTATGTGGCC(T)17-3′ (10 pmol/μl; custom order, Invitrogen) for the TSS/PAS libraries and with the random hexamer adaptor primer: 5′-GCGGCTGAAGACGGCCTATGTGGCCCAGCAGNNNNNNC-3′ (10 pmol/μl; custom order, Invitrogen) for the TSS/Random libraries. The first strand cDNAs were used for polymerase chain reaction (PCR) amplification using GeneAmp (PerkinElmer). PCR was performed according to the following protocol: 5 min at 94°C; 20 cycles at 94°C for 1 min, 58°C for 1 min and 72°C for 10 min; and a final extension for 10 min at 72°C. For the PCR primers, we used the 5′-primer: 5′- AGC ATC GAG TCG GCC TTG TTG-3′ with biotin labels on the 10th, 16th and 17th thymidines (10 pmol/μl; custom order, Invitrogen) and the 3′-primer 5′-GCG GCT GAA GAC GGC CTA TGT-3′ (10 pmol/μl; custom order, Invitrogen). The PCR products were electrophoresed on 1% agarose gels and recovered in the size range of 0.5–5 kb for the TSS/PAS libraries and in the three size ranges 0.5–1 kb, 1–2 kb and 2–5 kb for the TSS/Random libraries.
- *Circularization and Illumina adaptor ligation.* To construct mate pair libraries from 600 ng of prepared full-length cDNA fragments, we used the Mate Pair Library kit v2 (Illumina). End repair of the biotinylated PCR products was performed by incubating them with 10-μl End Repair buffer (Illumina), dH2O, deoxy nucleic acid triphospate (dNTP) (Illumina), 5-μl T4 DNA Polymerase (Illumina), 5-μl T4 Polynucleotide Kinase (Illumina) and 1-μl Klenow DNA Polymerase (Illumina) for 30 min at 20°C. The end-repaired DNA was circularized with 206.6-μl dH2O, 30-μl 10X Circularization Buffer (Illumina) and 13.4-μl Circularization Ligase (Illumina) for 16 h at 30°C. The linear DNA was digested by incubating with 3 μl of DNA exonuclease (Illumina) for 20 min at 37°C followed by reaction at 70°C for 30 min. The reaction was then stopped by adding 12 μl of ethylenediaminetetraacetic acid (EDTA) (Illumina). Following phenol/chloroform extraction and ethanol precipitation, the circularized DNA was digested with 20-U EcoP15I (NEW ENGLAND BioLabs) for 1 h at 37°C. EcoP15I was then inactivated by heating the reaction at 65°C for 20 min. For the TSS/PAS libraries, digested DNA fragments were additionally fragmented by nebulization at 32 psi for 6 min. After fragmentation, the DNA fragments containing biotin groups were attached to streptavidin beads (Dynabeads M-280, Invitrogen) for the A-tailing and adaptor ligation end-repair reactions. For the adaptors, we used Adaptor Oligo Mix for paired-end sequencing (Illumina). Final round PCR was performed as follows: 30 s at 98°C; 18 cycles at 98°C for 10 s, 65°C for 30 s, 72°C for 30 s and 72°C for 5 min using PCR primer 2.0 (Illumina). The PCR products were electrophoresed on 8% polyacrylamide gels, and the size fractions of 280–300 bp were recovered for sequencing. The quality and quantity (yield) of the intermediate products were inspected using BioAnalyzer (Agilent) after each step.

### Sequencing

Sequencing was conducted on the Illumina HiSeq2000/GAIIx platform following the manufacturer's instructions. Paired-end reads of 101 bases were generated for at least 10 million tags for each library.

### ChIP Seq

DLD-1, HeLa, HEK293 and MCF7 cells (1 × 10^8^ cells) were fixed in 1% formaldehyde at room temperature for 10 min and then quenched in 208-mM glycine for 5 min at room temperature. The cells were washed twice with phosphate buffered saline, harvested and then lysed in 5 ml of Lysis Buffer 1 (50-mM HEPES–KOH, pH 7.5, 140-mM NaCl, 1-mM EDTA, 10% glycerol, 0.5% NP-40, 0.25% Triton X-100). The lysates were incubated at 4°C for 10 min and centrifuged at 250 ×*g* for 5 min at 4°C. The pellets were then resuspended in 5 ml of Lysis Buffer 2 (10-mM Tris–HCl, pH 8.0, 200-mM NaCl, 1-mM EDTA, 0.5-mM ethylene glycol tetraacetic acid (EGTA)), incubated at room temperature for 10 min and centrifuged at 250 ×*g* for 5 min at 4°C. These pellets were resuspended in 1 ml of Lysis Buffer 3 (10-mM Tris–HCl, pH 8.0, 100-mM NaCl, 1-mM EDTA, 0.5-mM EGTA, 0.1% Na-deoxycholate, 0.5% N-lauroylsarcosine) and sonicated (TOMY SEIKO) for 16 cycles of 30 s each on ice. Then, 100 μl of 10% Triton-X 100 was added to the samples, and the cell lysates were centrifuged at 17 800 ×*g* for 10 min. A 50-μl sample of the supernatant was saved for the controls (whole cell extract, WCE DNA). Washed magnetic beads bound to 10 μg of rabbit monoclonal anti-RNA polymerase II antibody (Abcam, ab817), monoclonal anti-H3K4me3 antibody (Abcam, ab1012), monoclonal anti-H3K4me1 antibody (Abcam, ab8895), monoclonal anti-H3K27me3 antibody (Abcam, ab6002), polyclonal anti-H3K27Ac antibody (Abcam, ab4729), polyclonal anti-H3K36me3 antibody (Abcam, ab9050), polyclonal anti-polII pS2 antibody (Abcam, ab5095), polyclonal anti-CTCF antibody (Millipore 07-729) or polyclonal anti-Rad21 antibody (Abcam, ab992) were added to the supernatant. The samples were rotated at 4°C overnight and washed eight times with 1 ml of wash buffer (50-mM HEPES–KOH, pH 7.5, 500-mM LiCl, 1-mM EDTA, 1% NP-40, 0.7% Na-deoxycholate) and once with Tris-EDTA (TE) buffer containing 50-mM NaCl. The sample (IP) was then eluted with 200 μl of elution buffer (1-M Tris–HCl, pH 8.0, 0.5-M EDTA, pH 8.0, 1% sodium dodecyl sulphate) at 65°C for 15 min. The eluates were transferred to new tubes and incubated at 65°C overnight. Concurrently, 150 μl of elution buffer was added to the saved WCE-DNA, and the samples were incubated at 65°C overnight. The next morning, 200 μl of TE buffer and 8 μl of 10 mg/ml RNase A (Funakoshi) were added to the IP and WCE-DNA samples, which were then incubated at 37°C for 2 h. Subsequently, 4 μl of 20-mg/ml proteinase K (TaKaRa) was added to the samples, which were then incubated at 55°C for 2 h. The DNA samples were purified using phenol/chloroform extraction and ethanol precipitation. The samples for ChIP Seq by Illumina HiSeq2000/GAIIx were prepared according to the manufacturer's instructions.

### Computational procedures

We used the sequence CTGCTGCC to determine the TSS for all of the libraries. For the internal 3′-end of cDNA, we used the sequence CTGCTGGG, which is the sequence of the adaptor following the cDNA. The statistics of the tags used for the following process are shown in Supplementary Figure S1. We independently clustered the TSS and PAS tags using 500-bp bins. We associated the clusters with RefSeq genes (UCSC Genome Browser; hg19; http://genome.ucsc.edu/; NM as protein-coding genes and NR as putative non-coding genes) when the cluster was located from 50 kb upstream of the 5′-end of the transcript model in the case of TSCs and 50 kb downstream from the 3′-end in the case of PACs. We did not consider TSCs and PACs located within the internal exons of the RefSeq transcripts. The expression levels of the TSCs and PACs were calculated based on their tag counts. Expression levels, as represented by parts per million tags (ppm), were used for the analyses. For further details, see (25) and our database.

To examine the tissue specificity of the expression patterns, Z-scores were calculated as follows:

\begin{equation*} z = (x - \mu )/\sigma ; \end{equation*}

where *x* is the tag counts in log2 (ppm), *μ* is the mean of *x* and σ is the standard deviation of *x*.

To analyze *cis*-elements in the upstream or downstream regions of TSCs and PACs, we used TRANSFAC (version 2011.1; http://www.gene-regulation.com/pub/databases.html) with the cutoff values of minFP to search for potential TATA boxes (V$TATA_01, V$TATA_C). For CpG islands, information from the UCSC Genome Browser was used. For the poly(A)-addition signals, we screened for perfect matches to AATAAA. The statistical significance of the difference was calculated using the methods indicated in the respective legends. Statistical analyses were conducted using ‘R’ (http://www.r-project.org/).

To select ‘preferred’ TSC–PAC relationships, we first selected TSC-PACs with >5 ppm tags and >10 tags. Among these TSC-PACs, we selected the relationships for which the statistical bias, evaluated by the deviations from random selection assuming Poisson's distribution, was *P* < 0.05. We further selected cases in which the mutual selection of the TSC-PAC pair was the most frequent among any other pairs belonging to the same gene. For a schematic representation of the selection, see Supplementary Figure S4A.

For ChIP Seq analysis, we used MACS (v.1.4) with the default parameters. Namely, peaks giving *P* < 1e−5 were regarded as positives. To further assess the ChIP Seq patterns, we calculated the fold densities of the tags from the immunoprecipitations and input fractions for each genomic coordinate. To draw the graph, we considered the indicated genomic range and selected the genomic coordinate giving the largest fold density as the center for each of the genes. Transcript regions were normalized down to the indicated scale.

To identify fusion gene transcripts, we selected the cases in which >5 ppm of the TSC-PAC tags spanned different genic regions on the same chromosome but were separated by >3 Mb or where they were on different chromosomes in the four cancerous cell lines. For this purpose, only the tags with a mapping quality score of >37 were considered.

To assemble the tags, we followed the assembly scheme shown in Supplementary Figure S8A. Briefly, we allocated the genomic coordinate as ‘transcript’ when it was covered by at least one tag. Genomic coordinates that were spanned by at least one split tag were regarded as ‘intron’. The integrity of the assembly was evaluated as the proportion of the coordinates allocated either to ‘transcript’ or ‘intron’ compared to the total number of coordinates ranging from TSCs to PACs.

## RESULTS

### Construction of TSS/PAS libraries

According to the scheme shown in Figure 1A, we constructed TSS/PAS libraries from 14 human tissues and four cell lines. Details of the library construction procedure are described in the Supplementary Method with representative images. For further details, visit our web site at http://dbtss.hgc.jp/cgi-bin/protocol_matepairLibrary.cgi (open to anonymous visitors). Quality controls for the intermediate products are also described there. The qualities of the RNA materials are shown in Supplementary Figure S1A. We generated at least 10 million 101-base-paired-end sequence tags from each library on the Illumina platform. We assumed that each of the tags represented a TSS and a PAS at either end (see Supplementary Figure S1B for sequencing statistics). Using the TSS/PAS libraries, we first clustered the TSS tags to identify TSSs clusters (TSCs), as in our previous study (25). Similarly, we used PAS tags to identify PACs.

To remove noise and erroneous identification of the TSC and PAC relationships, we tentatively selected TSCs/PACs that were represented by >5 ppm TSS/PAS tags. We particularly expected that erroneous identification of TSSs or PASs derived from truncated cDNAs due to the inaccurate replacement of the cap structure or the internal priming of the oligo-dT primers would be removed by this filter. To examine to what extent this filter should work, we calculated the statistical significance in the occurrence of the clusters of >5 ppm, assuming Poisson's distribution for the randomly distributed tags. As shown in Figure 2C, we found that such events were *P* < 1e−10 in >94% and >91% of the TSCs and PACs of >5 ppm, respectively. Particularly for the PACs, we calculated the frequencies of the polyA stretch, which can serve as erroneous internal priming sites for oligo dT primers during first strand synthesis. We found that there were no such sequences for >74% of cases (right panel; Supplementary Figure S2G). We also examined the presence of the poly(A)-addition signals in the proximal regions. We found canonical poly(A)-addition signals in the 72% of the cases, which was consistent with a previous paper (left panel; Supplementary Figure S2G). We further assumed that the erroneous tags were distributed randomly throughout the transcripts and that chimeric transcripts, which inevitably form at the ligation steps during library construction, occurred between random pairs of TSSs and PACs. Thus, those products would not yield TSS/PAS tags of >5 ppm for a given pair of genes. Indeed, we found that the tags spanning different genes with a tag concentration of >5 ppm represented true transcripts that were derived from chromosomal rearrangements in cancerous cells (this issue will be discussed below; Figure 8D; also see Supplementary Figure S6). Finally, we evaluated the ligation bias of the synthetic oligo to the first base of the RNA [proposed as cause for caution by a previous paper, (26)] and found that the bias induced at this step should be reasonably small (Supplementary Figure S2H).

We identified a total of 44 902 TSS-PAS unique pair clusters from 18 libraries, with an average of 8890 TSC/PAC pairs per library, which collectively represented 10 038 genes (25 600 TSCs/PACs) out of 18 808 total RefSeq genes (Table 1). We associated the TSCs with the RefSeq gene when the clusters were located within 50 kb upstream of the 5′-end of the transcript model. This criterion was adopted because the distances between the first and the second exons were less than 50 kb in 92% of the known cases (Supplementary Figure S2I). In addition, 574 known lncRNAs (818 TSCs/PACs) and lncRNAs of 5709 TSCs/PACs from unknown transcripts were identified. We verified the identification of the TSCs and PACs by comparing their positions with the positions of RefSeq transcripts. As shown in Figures 1B and 2A and B, 92% of the TSC tags and 78% of the PAC tags were located within or upstream/downstream from regions of the 5′- or 3′-terminal exons of the RefSeq transcript model, respectively. We also attempted to validate the represented transcripts by independent reverse transcriptase-PCR (RT-PCR). We were able to validate the correct identification of the putative full-length transcript in representative cases (Supplementary Figures S2 and S3; also see Supplementary Figure S9).

**Table 1.** Statistics on the TSS and PAS tags generated and characterized in the present study

	Number of library	Number of tags generated	Number of tags mapped to upstream of RefSeq gene or to first exon (%)	Number of tags mapped to downstream of RefSeq gene or to last exon (%)	Number of NM genes with >5-ppm tags	Number of NR genes with >5-ppm tags	Number of genes containing multiple TSCs	Number of genes containing multiple PACs
Average	-	4 016 453	92	79	5983	107	281	1033
Total	18	72 296 154	91	76	10 038	574	2488	5096

NM: RefSeq NM genes; NR: RefSeq NR genes; TSC: transcription start site cluster; PAC: poly(A)-addition site clusters.

Finally, we compared the locations of the TSCs and PACs identified in the current study with the ones identified in previous studies. We first used the ENCODE data, which were produced from HeLa cells by the RNA-PET method. In this method, the ends of the full-length cDNAs cloned in the vector are excised and adjoined to generate a tag, which are subjected to next-generation sequencing (23). We found 85% of the TSCs and 82% of the PACs in the ENCODE data to be covered by our data set, respectively, suggesting that both methods should be useful to analyze the transcript structures (Supplementary Figure S2D). Conversely, only 20% of the TSCs and 15% of the PACs of our data set were covered by the ENCODE HeLa data, perhaps because we analyzed a wider variety of tissues and cell types. We also used another data set for comparison in which poly(A)-addition sites were analyzed as ‘PAS’ tags for 13 types of tissues (11). In this case, we found that 21% of their PACs overlapped ours (Supplementary Figure S2F). Conversely, 29% of their PACs were represented in our data set. We were uncertain of the cause of these non-overlapping populations, but, again, the explanation might be that different target tissues were used for the respective studies. Notably, we compared and found that the features of polyA addition signals in the areas surrounding PACs were similar between these two studies, suggesting that both methods correctly capture the poly(A) sites (Supplementary Figure S2G).

Taken together, based on these results, we concluded that most of the TSS/PACs identified by this method should generally represent the true termini of the transcripts.

### Characterization of alternative TSCs and PACs

As previously reported (17–19,25), we detected multiple TSCs in individual RefSeq genes in a number of cases, which may represent the results of alternative promoters (Figure 3A). Similarly, we sometimes detected multiple PACs in a given gene, which may represent the results of alternative transcriptional termination sites (Figure 3B). For the putative protein-coding NM genes, we detected a total of 6944 multiple TSCs (in 2488 genes) and a total of 16 755 multiple PACs (in 5096 genes) (upper panels; Figure 3C). Similarly, we detected multiple TSCs and PACs for a number of cases for putative non-protein coding NR genes (lower panels; Figure 3C). Unexpectedly, we found that the total number of multiple PAC-containing genes was as large as the total number of TSCs, suggesting that regulations mediated by alternative PACs may be as diverse as the regulations mediated by alternative TSCs. We further examined the presence of canonical TATA boxes (27,28) and CpG islands (29,30) around the TSCs and the presence of poly(A)-addition signals around the PACs (Figure 4A and B). We found that putative alternative TSCs preferentially contained TATA (−)/(−) combinations, while CpG (+)/(−) combinations were the most frequent. These observations were consistent with the results expected from previous analyses (17,25). However, pairs of PACs that both contained poly(A)-addition signals (+)/(+) were rare, which may indicate that multiple PACs receive distinct controls at the poly(A)-addition step.

We also examined the tissue specificity of the occurrences of TSCs and PACs. We calculated the Z-scores of the TSS/PAS tag counts (see the Materials and Methods section). When we tentatively selected TSCs-PACs with Z-scores >2, we found a 16% and 12% tissue-preferred presence of TSCs and PACs, respectively (Figure 4C). When we compared the distributions of the Z-scores of TSCs and PACs, we found that the tissue biases in the occurrences were more significant for TSCs (*P* = 1E−62; Figure 4D). Taken together, these results may indicate that distinct regulations are exerted at the transcriptional initiation step and the termination step.

### Identification and characterization of preferred TSC-PAC pairs

Particularly when there were multiple pairs of TSCs and PACs within a single gene, we examined whether there were any correlations between the TSCs and PACs. We calculated the representation frequencies of the tags for TSCs and PACs in the TSS/PAS libraries, assuming a Poisson distribution for random selection. In the majority of cases (24 833 of the 25 600 cases; 97%), there was no significant correlation between the TSCs and PACs, but rather their selection appeared to be independent. Nevertheless, we found statistically significant correlations (*P* < 0.05) in 767 cases in 372 genes, where TSCs and PACs were associated and where more than 50% of the total tags associated with either the TSC or the PAC corresponded to that particular pair (we call these cases ‘preferred’ TSC-PACs hereafter; see the Materials and Methods section; examples for the detected cases are shown in Figure 5). We analyzed and found that the frequencies and combinations of the TATA boxes and CpG islands around the TSCs and the poly(A)-addition signals were similar to the values in the total population (Supplementary Figure S4B and C).

We also analyzed to what extent potential protein-coding regions were mutually different between the pairs of ‘preferred’ TSCs and PACs. We found that the ‘preferred’ pairs shared only small parts of the potential coding regions. Occasionally, they did not share any coding regions, as if they consisted of two distinct genes. Indeed, the proportion of the shared coding DNA sequences was significantly smaller than the proportion of ‘uncorrelated’ TSC-PAC pairs (*P* = 3E−78; Figure 5A). As exemplified in the case of the *PKIA* gene in Figure 5B (upper panel), TSC-PAC Unit A was completely separated from TSC-PAC Unit B. We further examined the expression patterns of the two units by calculating their Z-scores. For the case of the *PKIA* gene, two units, which did not overlap, also showed distinct tissue expression preferences (upper panel; Figure 5C). Unit B showed the highest Z-scores in the heart (*Z* = 4.5) and skeletal muscle (*Z* = 3.0), while Unit A was expressed almost unbiased with the highest Z-score of *Z* = 0.7 in the testis. In the case of the *SLC25A27* gene (lower panels; Figure 5B and C), Unit B was selectively expressed in the testis (*Z* = 2.8), while Unit A had expression biases in the brain (*Z* = 3.3).

We then overlaid the Z-scores from each of the TSC-PAC pairs for all 767 cases (Figure 5D). When we counted the pairs located outside the lines indicated in Figure 5D, we found that the most frequently observed tissue-specific differential use of the TSC-PAC pairs was the brain and testis (Figure 5E). We further determined which functional categories of genes were enriched in those genes harboring ‘preferred’ TSC-PACs by Gene Ontology (GO) term enrichment analysis. We found that ‘GTPase activity-related genes’ were particularly enriched (*P* = 8E−06; Table 3). Note that we selected GO terms having *P*-values of *P* < 1e−5, though there is no solid rationale for this selection. See (31) for a detailed discussion on selecting cutoffs for the GO term enrichment analysis in general. Also, even at these *P* values, statistical enrichment seemed not very drastic. Nevertheless, it is still possible that, for these genes, different units of the same gene may be utilized by different tissues via distinct regulatory mechanisms.

**Table 2.** Statistics on the TSS and Random tags generated and characterized in the present study

	Number of library	Number of tags generated	Number of tags mapped to upstream of RefSeq gene or to the first exon (%)	Number of NM genes covered by >90%	Number of NM genes with all exons covered by the tag	Number of NR genes covered by >90%	Number of NR genes with all exons covered by the tag
Average	-	6 458 097	89	647	1807	66	332
Total	18 (54)	348 737 226	93	9254	12 168	497	1596

The total number of libraries in parentheses reflects the three different size fractions from each of the 18 tissues or cell lines. NM: RefSeq NM genes; NR: RefSeq NR genes.

**Table 3.** Genes with preferred TSC-PAC pairs

GO ID	GO term	Number of genes with preferred TSC-PAC	Number of genes in total population	*P*-value	False detection rate
0005096	GTPase activator activity	15	195	8E−06	0.0016
0043547	Positive regulation of GTPase activity	12	134	1E−05	0.0016

Results of the GO term enrichment analysis. Enrichment of the terms was evaluated by calculating hypergeometric distributions; statistical significances are shown in the last column. The numbers of genes associated with the indicated GO terms in the genes with ‘preferred’ TSC-PACs and in the total population are shown in the third and fourth columns, respectively. To remove too loose or too tight GO terms, only GO terms having 100–500 genes were considered. The *P*-values are from the GO term enrichment analysis. The *P*-values in the main text are calculated based on the deviations in tag counts regarding expression patterns and connectivity of the transcripts. Hits giving *P*-values of *P* < 1e−5 are shown. Note, in general, the *P*-values in the tables are supported large numbers of the data points (tag counts) than the former case (number of genes), thus may have given more significant *P*-values.

### Relation of transcript structure and chromatin structure

To examine the relationship between transcript structure and chromatin status, we generated ChIP Seq data of representative histone modifications (H3K4me1, H3K4me3, H3K27Ac, H3K27me3 and H3K36me3), polymerase II in its initiation (Pol II) and elongation (CTD-PS2) forms, and representative components of the chromatin insulator complex (Rad21 and CTCF) in four cultured cell lines. An average of 34 million sequence tags were generated for each data set and used for the analysis. The statistics of the tags are shown in Supplementary Figure S1D. ‘Peaks’ were called using MACS (http://liulab.dfci.harvard.edu/MACS/) by default parameters. For further details on the procedure, and real-time PCR validations for selected independent cases, see Supplementary Figure S5.

As shown in Figure 6, we analyzed the ChIP Seq patterns surrounding the transcript regions between TSCs and PACs. Consistent with previous reports, we frequently detected peaks of the enhancer mark H3K27Ac in the upstream regions of the TSCs for the active transcript regions (32,33). The peaks of H3K4me3 and Pol II overlapped with the TSCs (34–38). The signals of H3K36me3 were significant in the transcribed regions (39), while the signals of H3K27me3 were absent from these regions (37,40). Peaks of Pol SII often accumulated at PACs (41,42). Generally, peak patterns were more significant for transcripts with high expression levels (Figure 6A and B). Interestingly, although signal intensities were different between highly and lowly expressed transcripts, the shapes of the peaks were generally similar (Figure 6A and B). Similar results were obtained from all cell lines (Figure S5B). Collectively, these results support the concept that our TSC-PAC data were generally well correlated with chromatin signatures and can thus be used for transcript annotations in addition to or instead of ChIP Seq data. It should also be noted that the TSC-PAC information was directly obtained from the transcriptome analysis, which has a base-level resolution, and should thereby have unique advantages for the precise identification of the transcript regions in a given cell type.

We also analyzed the association between the above-mentioned ‘preferred’ TSCs-PACs and the chromatin signatures. In these cases, we also observed chromatin features similar to the features in the total RefSeq transcripts (Figure 6C and D), indicating that the ‘preferred’ TSC-PACs should also represent transcripts that are actually transcribed in the respective cell types (Supplementary Figure S5C). We further examined whether there were any factors separating genomic regions of the TSC-PAC pairs that might explain their ‘preferred’ usage within a single gene. An examination of the ChIP Seq peaks of Rad21 (43,44) and CTCF (45,46) showed that they were enriched in the regions separating the TSC-PAC pairs compared to other intronic regions of the same gene (Figure 7A and B). We could not distinguish whether this observation was a cause of the TSC-PAC associations or a consequence of them; however, we believe that the transcriptions of the ‘preferred’ TSC-PAC associations are regulated in a deterministic manner rather than being biological noise or experimental artifacts.

### Utilization of the TSC-PAC information for identifying diversely transcribed regions and fusion gene transcripts

During the course of the analysis, we also detected that TSCs and PACs sometimes connected two adjacent but distinct RefSeq genes (for examples and their RT-PCR validations, see Supplementary Figure S7). These cases may be regarded as if ‘preferred’ TSC-PACs occurred between the RefSeq genes. We searched for and identified a total of 172 such cases and examined their chromatin signatures. Similar to the ‘preferred’ TSC–PAC relationships, we detected characteristic chromatin signatures for each of the two adjacent RefSeq genes. Moreover, the levels of H3K36me3 were higher in the genomic regions between the two RefSeq genes (Figure 8A). We also analyzed the frequencies of the Rad21 and CTCF binding sites. We found that their peaks were less enriched than other intergenic regions of the same length, when transcripts adjoining two RefSeq genes were actively transcribed (Figure 8B and C). Based on these results, we believe that similar transcriptional diversification of gene functions also takes place for these cases. Diverse transcription may occur not only in an intragenic but also in an intergenic manner.

We also utilized the TSC-PAC information to identify fusion gene transcripts, which result from the pathological conjoining of two different gene units in cancerous cell lines. Recent studies have demonstrated that such transcripts are formed by chromosomal aberrations in a wide variety of cancers (47,48), occasionally encoding proteins of mal-functions, and thus serve as the so-called drivers of carcinogenesis (49,50). We searched for such fusion transcripts in the data sets of the cancerous cell lines LC2AD, MCF7 and DLD1 and identified two candidates (for details of the detection pipeline, see the Materials and Methods section; also note that with this filter essentially no other candidates remained except these two; see Supplementary Figure S6A for more details). As shown in Figure 8D, in the LC2AD lung cancer cell line, the TSC of the *CCDC6* gene was physically connected to the PAS of the *RET* gene (51); the two genes were separated by more than 3 Mb and located on opposite strands of chromosome 10. This fusion transcript was recently identified in ∼3% of lung adenocarcinoma patients (52) and is generating interest as a target for anti-cancer drugs. We also identified *BCAS4*/*BCAS3* fusion transcripts in MCF7 cells (53–55). The correct identification of these fusion transcripts was verified by independent RT-PCR by setting RT-PCR primers in the TSC and PAC regions and successfully amplifying the entire transcript (Figure 8 and Supplementary Figure S6). Based on these results, we concluded that the TSC-PAC information should also be useful for identifying and characterizing fusion gene transcripts.

### Use of the TSCs-PACs/Random tags for determining transcript structures

**Figure 9.**
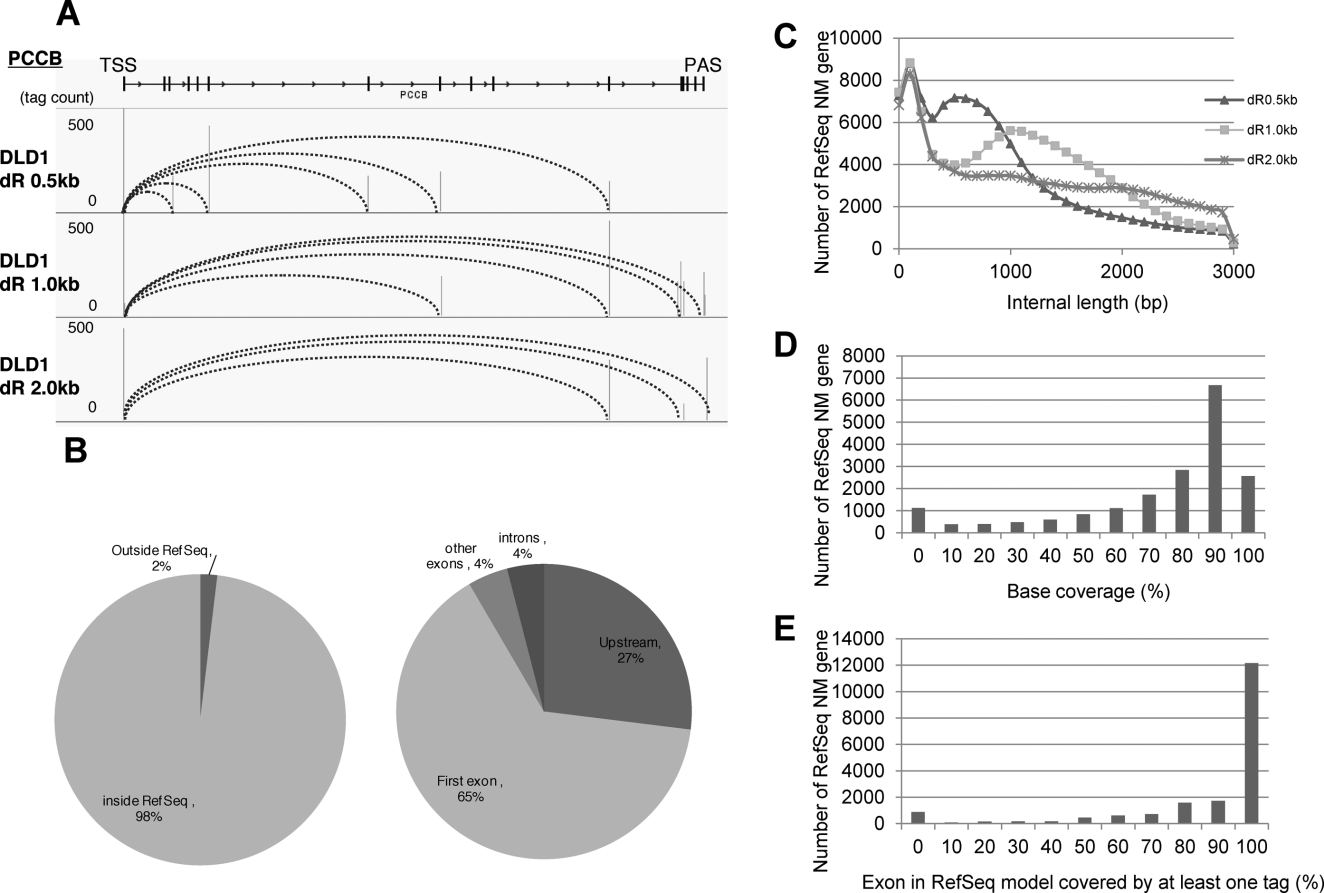
Construction and characterization of the TSS/Random cDNA libraries. (**A**) Examples of TSS-Random tags identified from the TSS/Random libraries. (**B**) Distribution of TSSs and PACs relative to the RefSeq transcript models in TSS/PAS libraries. (**C**) Distributions of size fractions (dR0.5, dR1.0 and dR2.0) of TSSs and internal tags in TSS/Random libraries. (**D, E**) Coverage of the exons in the RefSeq transcript model by 0.5-kb (left), 1-kb (middle) and 2-kb (right) TSS/Random library tags.

We also wished to ascertain the internal structure of the TSCs/PACs to precisely define transcript models. Similar to the TSS/PAS libraries, we constructed TSS/Random libraries using the same RNA materials (Figure 1A, with modifications to the procedure indicated by asterisks; Table 2). Random primer-primed and double-stranded cDNAs were size-fractionated at 0.5–1.0 kb (dR0.5), 1.0–2.0 kb (dR1.0) and >2.0 kb (dR2.0) and used to construct the library. Details of the protocol and a typical example of the intermediate products are shown in Supplementary Method and on our web site (http://dbtss.hgc.jp/cgi-bin/protocol_matepairLibrary.cgi). Examples of genes in these fractions are shown in Figure 9A. The statistical analysis of the mapped genomic coordinates of the tags showed that their 5′-ends accurately represented the TSSs; that is, 93% of the TSS tags were mapped to regions within 500 bases of the 5′-exons of the RefSeq transcript model (Table 2; Figure 9B; Supplementary Figure S1C for sequencing statistics). The distances between the TSS and Random tags on the RefSeq transcript models depended on the length of the size-fractionated cDNAs, as expected (Figure 9C). As a result, when all the tags from the 18 tissue types were merged, 49% of the RefSeq transcript regions were covered by more than 90% at the base level (Figure 9D), while for 74% of the RefSeq genes, all exons in the transcript model were covered by at least one tag (Figure 9E).

**Figure 10.**
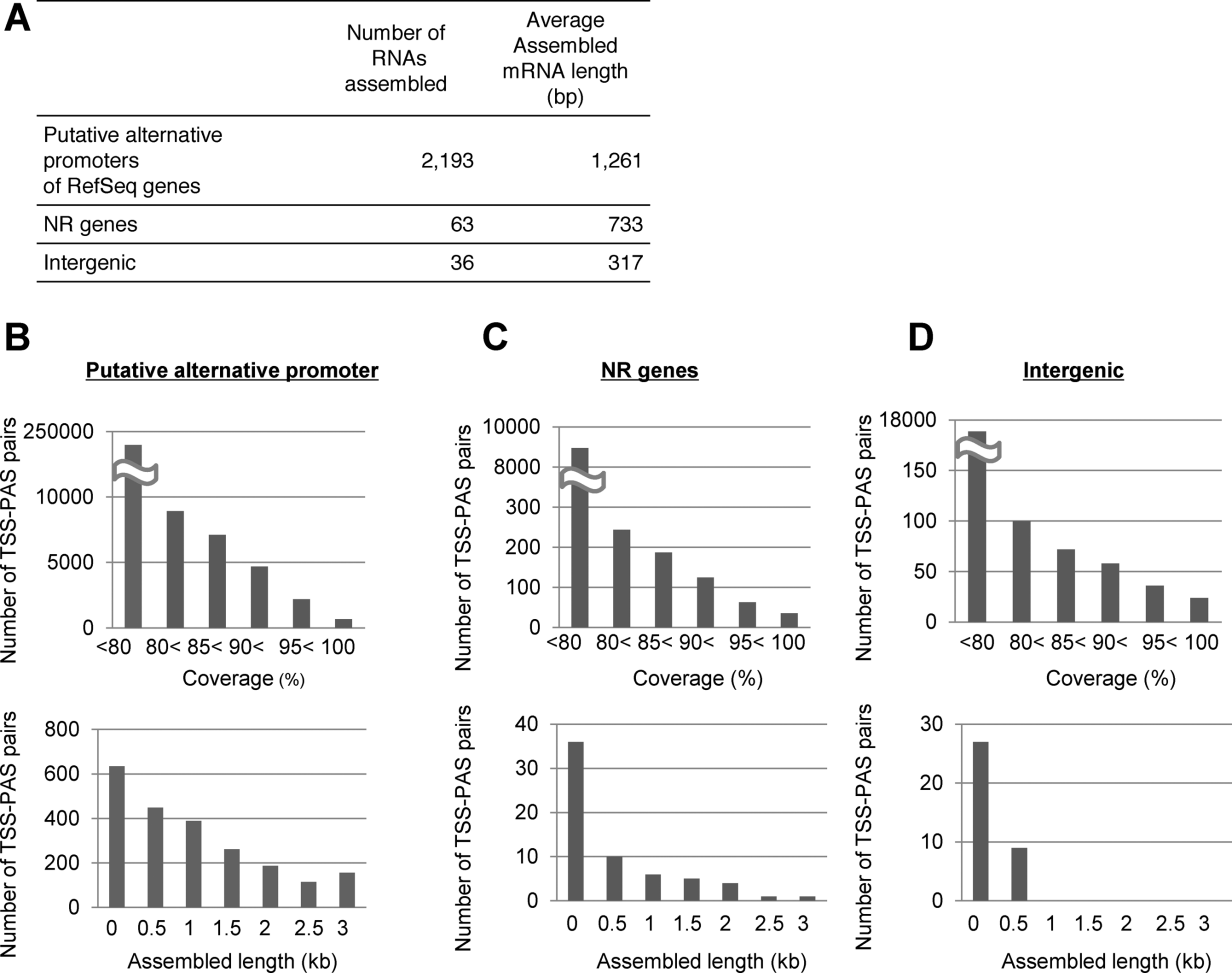
Mapped assembly of the transcripts of putative alternative promoter products and intervening lncRNAs. (**A**) Statistics of RNA assembly using TSS-PAS/Random tags. (**B**) Distributions of the numbers of successfully assembled transcripts at the indicated coverage for putative alternative promoter products (upper panel) and length distribution of the assembled transcripts (lower panel). (**C, D**) Results of the same analysis as in (B) for the NR genes and for the putative intervening lncRNAs.

We then used the recovered TSS-PAS/Random tags to assemble the sequence tags. In particular, we attempted to reconstruct transcript structures separately for the transcription products of potential alternative promoters. We used TSS tags to separate the different TSC groups and their paired PAS/Random tags to determine the transcript structures. Because we were concerned that the coverage of the tags might not be as high as in the usual RNA Seq, we used a genome-based approach for this assembly as previously described (56,57), rather than the *de novo* assembly approach (58–60) (see the Materials and Methods section; also see Supplementary Figure S8). As a result, the downstream products of 2292 TSCs for putative alternative promoters that did not overlap with RefSeq 5′-ends were successfully assembled with a coverage of >95% in the genomic regions between TSCs and PACs (Figure 10). On average, the assembled transcript length was 1261 bp, suggesting that the transcripts had sufficient coding potential (Figure 10A and B).

We also adopted a similar strategy to assemble the intervening lncRNAs (Supplementary Figure S9) and thereby determined the downstream structure of the lncRNAs in the RefSeq NR database and of previously unreported intergenic TSC-PAC pairs. For the intergenic TSC-PACs that did not overlap with the RefSeq NR transcripts, the overall success rates were similar. The overall success rate of these latter assemblies was somewhat worse than for the RefSeq genes, perhaps due to their low expression levels. Nevertheless, we successfully assembled 63 of the RefSeq NR transcripts and 36 other intervening putative lncRNAs (Figure 10A, C and D). These sequences should be an indispensable foundation to further infer the biological roles of alternative promoter products and lncRNAs, for which complete sequence structures have not been precisely determined in all cases.

## DISCUSSION

In this paper, we described the construction and characterization of TSS/PAS and TSS/Random libraries. Although a similar method was partly described in a previous paper (22,23), this report is the first to make this approach practically applicable to human transcriptome analysis. By constructing a series of mate pair full-length cDNA libraries, we were able to investigate the relationships between TSCs and PACs. The correlation analyses between TSCs and PACs showed that the transcriptional units sometimes separate units of single genes or connecting units of distinct genes. Such diverse transcription has been reported based on full-length cDNA sequencing analyses; however, recent RNA Seq-based approaches representing only fragmented transcripts have not yielded sufficient information.

There are several drawbacks to the developed methodology. First, this method currently requires >10 µg of the starting total RNA material, perhaps due to an insufficient conversion rate of the cap structure with the synthetic oligoribonucleotide and the circularization of the full-length cDNAs. Thus, this approach cannot be applied to the cells of minor populations or cells from which only a small amount of total RNA can be extracted. Second, the extent to which bias was introduced at the step of RNA ligation and PCR amplification still remains elusive (for details on this issue, see Supplementary Figure S2H) (26). Finally, even finer size fractionation of the TSS–Random libraries with greater sequence depth would be necessary for the assembly to distinguish internal splicing patterns as well. Indeed, we have observed that there were a number of tags representing distinct splice variants that were not represented in the assembled sequences due to the limited coverage of such ‘split tags’ (for details on this issue, see Supplementary Figure S8).

In spite of several drawbacks, it is significant that our unique transcriptome resource has enabled us to refine transcript models. Without this method, it would be practically impossible to determine separate transcript structures for putative alternative promoter products. Additionally, it would be difficult to define transcript structures for lncRNAs, as the gene expression levels for these transcripts are generally low, and both TSSs and PACs are so widely distributed that saturation level sequencing imposes a large cost for RNA Seq. This method should be helpful for genome annotations not only for humans but also for newly sequenced genomes. We believe that by complementing current transcriptome analysis using mainly RNA Seq, this TSS-PAS/Random full-length cDNA library approach will offer new insights into the complex regulation of transcriptomes.

## ACCESSION NUMBERS

DRA001232.

## SUPPLEMENTARY DATA

Supplementary Data are available at NAR Online.

SUPPLEMENTARY DATA
