# Supplementary material for: Construction of mate pair full-length cDNAs libraries and characterization of transcriptional start sites and termination sites
Source: Nucleic Acids Res. 2014 Jul 17;42(16):e125. doi: 10.1093/nar/gku600 (PMC4176323; doi:10.1093/nar/gku600)
Supplement: SUPPLEMENTARY DATA [file supp_42_16_e125__index.html]

Construction of mate pair full-length cDNAs libraries and characterization of transcriptional start sites and termination sites — SUPPLEMENTARY DATA 

# Construction of mate pair full-length cDNAs libraries and characterization of transcriptional start sites and termination sites

## SUPPLEMENTARY DATA

**Files in this Data Supplement:**

- SUPPLEMENTARY DATA
- SUPPLEMENTARY DATA
- SUPPLEMENTARY DATA
