# Supplementary material for: Construction of mate pair full-length cDNAs libraries and characterization of transcriptional start sites and termination sites
Source: Nucleic Acids Res. 2014 Jul 17;42(16):e125. doi: 10.1093/nar/gku600 (PMC4176323; doi:10.1093/nar/gku600)
Supplement: SUPPLEMENTARY DATA [file supp_gku600_nar-00343-met-g-2014-File003.pptx]

## Slide 1
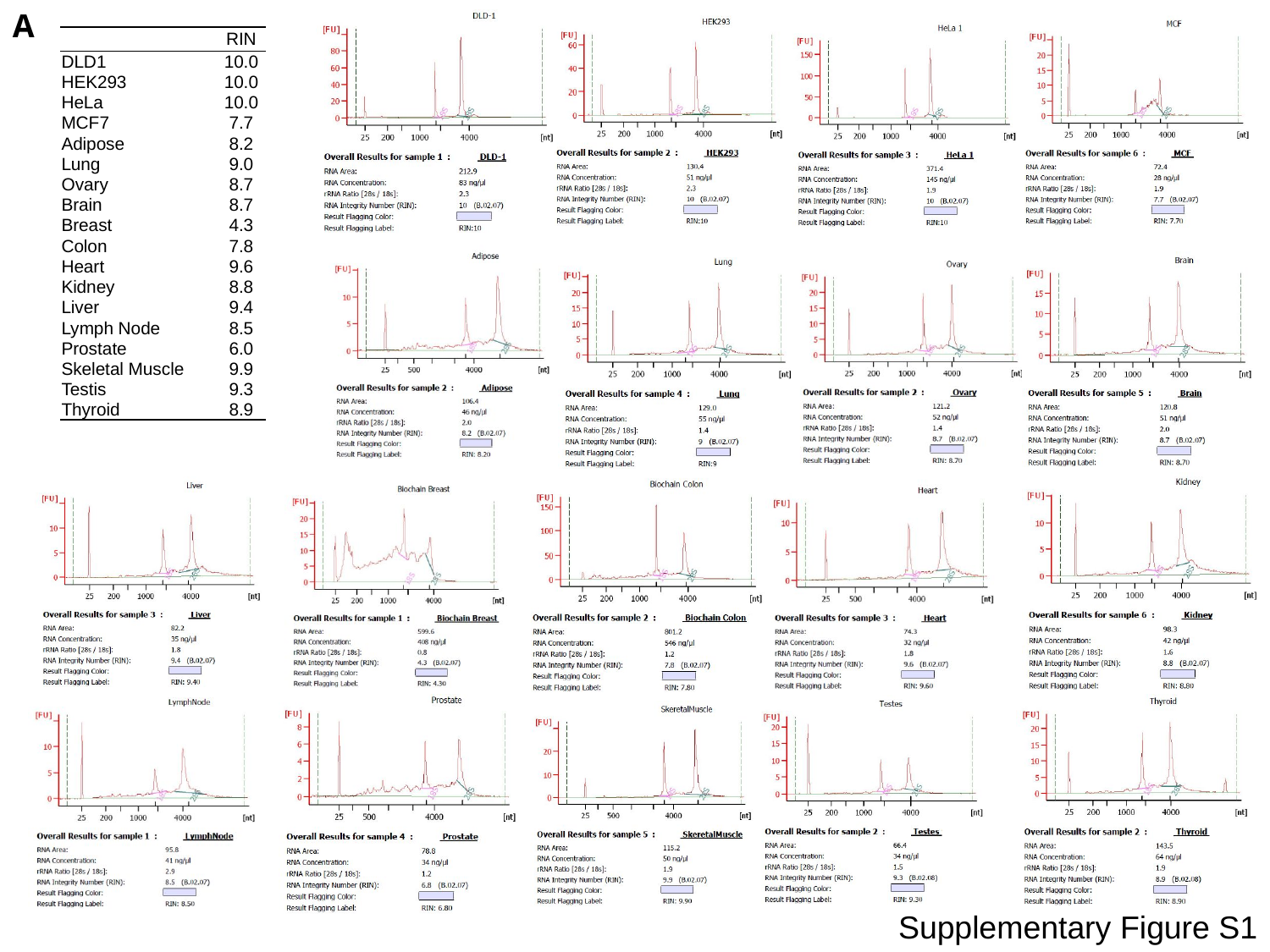

A
| | RIN |
| --- | --- |
| DLD1 | 10.0 |
| HEK293 | 10.0 |
| HeLa | 10.0 |
| MCF7 | 7.7 |
| Adipose | 8.2 |
| Lung | 9.0 |
| Ovary | 8.7 |
| Brain | 8.7 |
| Breast | 4.3 |
| Colon | 7.8 |
| Heart | 9.6 |
| Kidney | 8.8 |
| Liver | 9.4 |
| Lymph Node | 8.5 |
| Prostate | 6.0 |
| Skeletal Muscle | 9.9 |
| Testis | 9.3 |
| Thyroid | 8.9 |
Supplementary Figure S1

## Slide 2
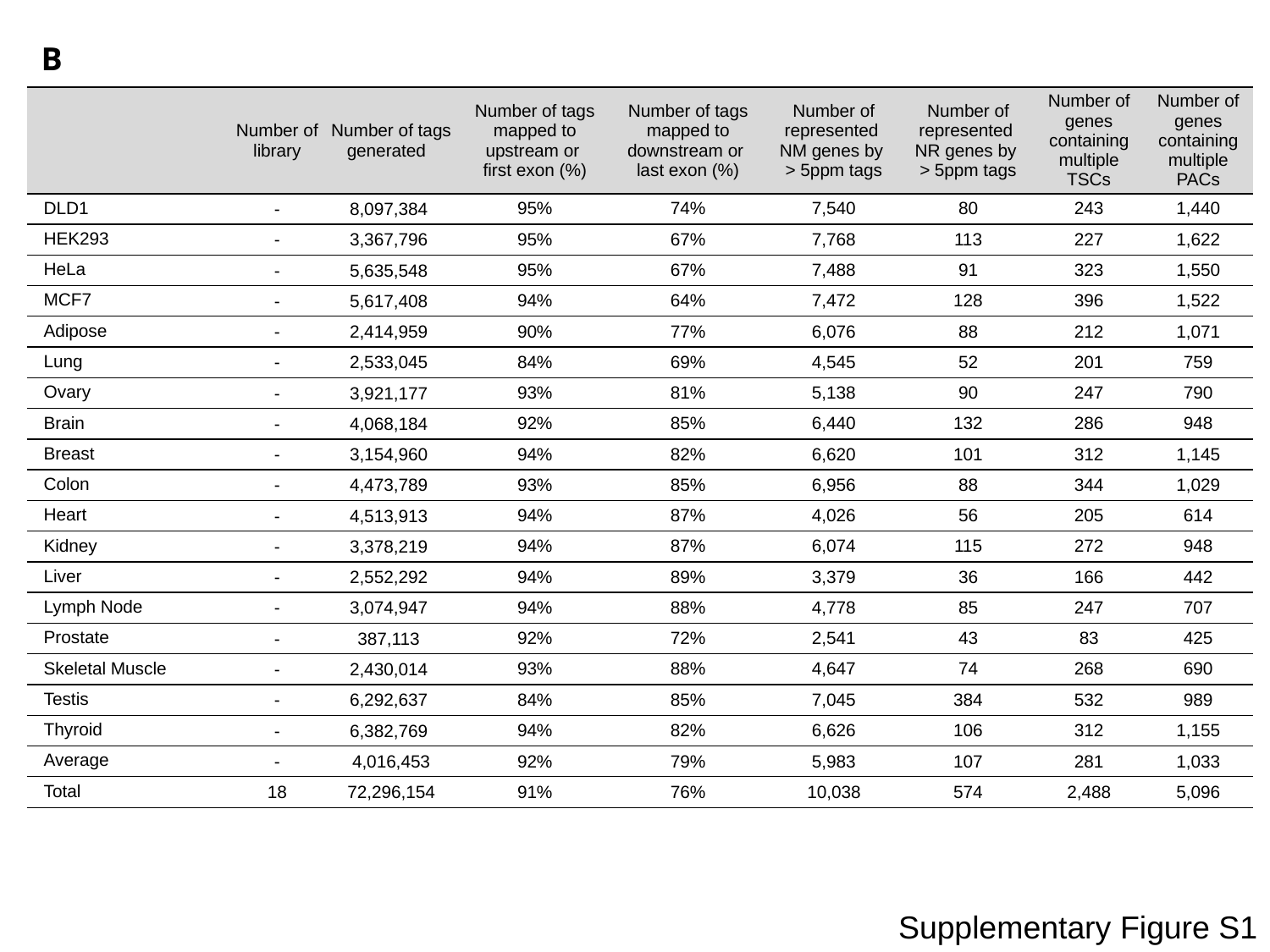

B
| | Number of library | Number of tags generated | Number of tags mapped to upstream or first exon (%) | Number of tags mapped to downstream or last exon (%) | Number of represented NM genes by > 5ppm tags | Number of represented NR genes by > 5ppm tags | Number of genes containing multiple TSCs | Number of genes containing multiple PACs |
| --- | --- | --- | --- | --- | --- | --- | --- | --- |
| DLD1 | - | 8,097,384 | 95% | 74% | 7,540 | 80 | 243 | 1,440 |
| HEK293 | - | 3,367,796 | 95% | 67% | 7,768 | 113 | 227 | 1,622 |
| HeLa | - | 5,635,548 | 95% | 67% | 7,488 | 91 | 323 | 1,550 |
| MCF7 | - | 5,617,408 | 94% | 64% | 7,472 | 128 | 396 | 1,522 |
| Adipose | - | 2,414,959 | 90% | 77% | 6,076 | 88 | 212 | 1,071 |
| Lung | - | 2,533,045 | 84% | 69% | 4,545 | 52 | 201 | 759 |
| Ovary | - | 3,921,177 | 93% | 81% | 5,138 | 90 | 247 | 790 |
| Brain | - | 4,068,184 | 92% | 85% | 6,440 | 132 | 286 | 948 |
| Breast | - | 3,154,960 | 94% | 82% | 6,620 | 101 | 312 | 1,145 |
| Colon | - | 4,473,789 | 93% | 85% | 6,956 | 88 | 344 | 1,029 |
| Heart | - | 4,513,913 | 94% | 87% | 4,026 | 56 | 205 | 614 |
| Kidney | - | 3,378,219 | 94% | 87% | 6,074 | 115 | 272 | 948 |
| Liver | - | 2,552,292 | 94% | 89% | 3,379 | 36 | 166 | 442 |
| Lymph Node | - | 3,074,947 | 94% | 88% | 4,778 | 85 | 247 | 707 |
| Prostate | - | 387,113 | 92% | 72% | 2,541 | 43 | 83 | 425 |
| Skeletal Muscle | - | 2,430,014 | 93% | 88% | 4,647 | 74 | 268 | 690 |
| Testis | - | 6,292,637 | 84% | 85% | 7,045 | 384 | 532 | 989 |
| Thyroid | - | 6,382,769 | 94% | 82% | 6,626 | 106 | 312 | 1,155 |
| Average | - | 4,016,453 | 92% | 79% | 5,983 | 107 | 281 | 1,033 |
| Total | 18 | 72,296,154 | 91% | 76% | 10,038 | 574 | 2,488 | 5,096 |
Supplementary Figure S1

## Slide 3
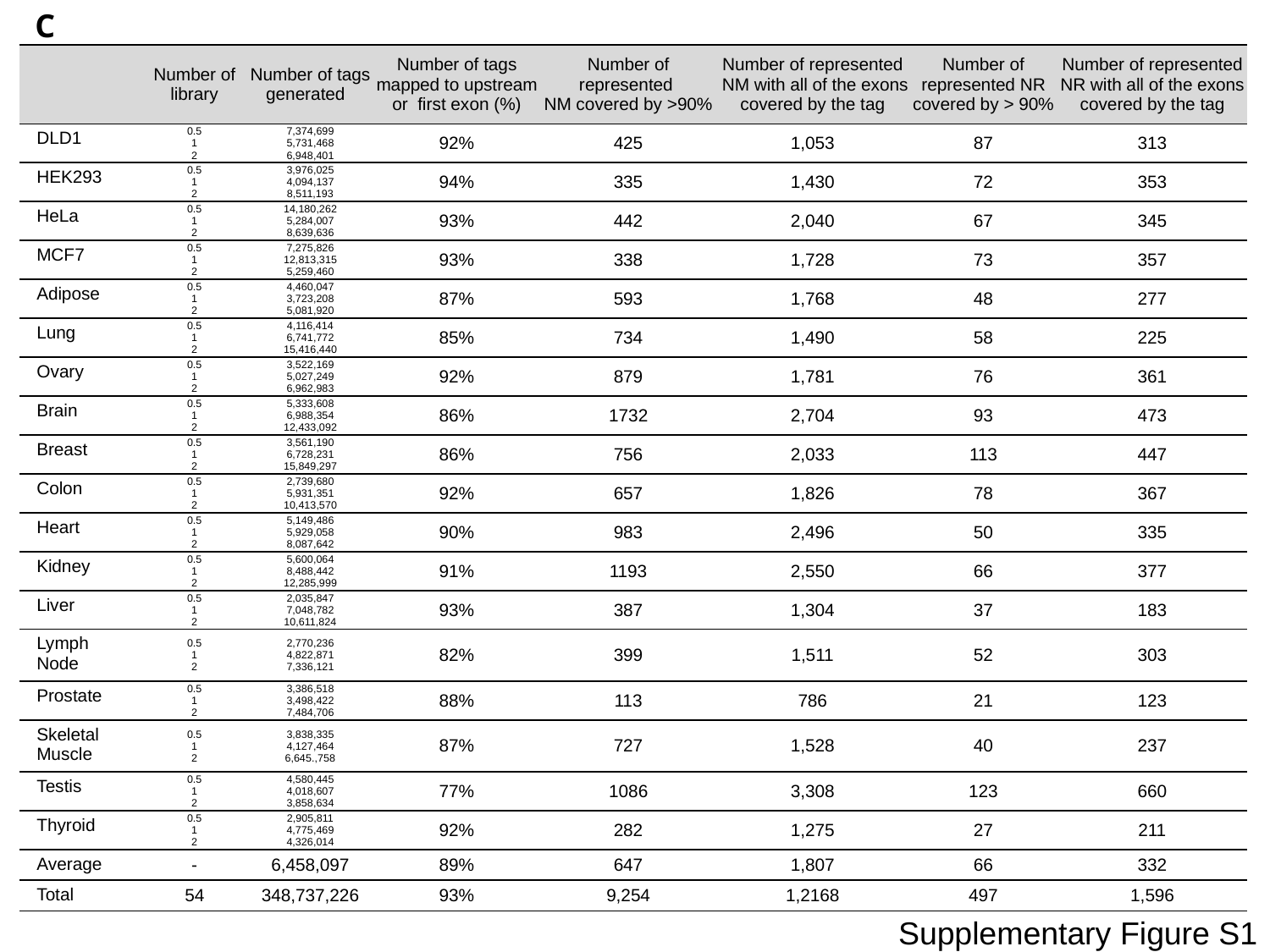

C
| | Number of library | Number of tags generated | Number of tags mapped to upstream or first exon (%) | Number of represented NM covered by >90% | Number of represented NM with all of the exons covered by the tag | Number of represented NR covered by > 90% | Number of represented NR with all of the exons covered by the tag |
| --- | --- | --- | --- | --- | --- | --- | --- |
| DLD1 | 0.5 1 2 | 7,374,699 5,731,468 6,948,401 | 92% | 425 | 1,053 | 87 | 313 |
| HEK293 | 0.5 1 2 | 3,976,025 4,094,137 8,511,193 | 94% | 335 | 1,430 | 72 | 353 |
| HeLa | 0.5 1 2 | 14,180,262 5,284,007 8,639,636 | 93% | 442 | 2,040 | 67 | 345 |
| MCF7 | 0.5 1 2 | 7,275,826 12,813,315 5,259,460 | 93% | 338 | 1,728 | 73 | 357 |
| Adipose | 0.5 1 2 | 4,460,047 3,723,208 5,081,920 | 87% | 593 | 1,768 | 48 | 277 |
| Lung | 0.5 1 2 | 4,116,414 6,741,772 15,416,440 | 85% | 734 | 1,490 | 58 | 225 |
| Ovary | 0.5 1 2 | 3,522,169 5,027,249 6,962,983 | 92% | 879 | 1,781 | 76 | 361 |
| Brain | 0.5 1 2 | 5,333,608 6,988,354 12,433,092 | 86% | 1732 | 2,704 | 93 | 473 |
| Breast | 0.5 1 2 | 3,561,190 6,728,231 15,849,297 | 86% | 756 | 2,033 | 113 | 447 |
| Colon | 0.5 1 2 | 2,739,680 5,931,351 10,413,570 | 92% | 657 | 1,826 | 78 | 367 |
| Heart | 0.5 1 2 | 5,149,486 5,929,058 8,087,642 | 90% | 983 | 2,496 | 50 | 335 |
| Kidney | 0.5 1 2 | 5,600,064 8,488,442 12,285,999 | 91% | 1193 | 2,550 | 66 | 377 |
| Liver | 0.5 1 2 | 2,035,847 7,048,782 10,611,824 | 93% | 387 | 1,304 | 37 | 183 |
| Lymph Node | 0.5 1 2 | 2,770,236 4,822,871 7,336,121 | 82% | 399 | 1,511 | 52 | 303 |
| Prostate | 0.5 1 2 | 3,386,518 3,498,422 7,484,706 | 88% | 113 | 786 | 21 | 123 |
| Skeletal Muscle | 0.5 1 2 | 3,838,335 4,127,464 6,645.,758 | 87% | 727 | 1,528 | 40 | 237 |
| Testis | 0.5 1 2 | 4,580,445 4,018,607 3,858,634 | 77% | 1086 | 3,308 | 123 | 660 |
| Thyroid | 0.5 1 2 | 2,905,811 4,775,469 4,326,014 | 92% | 282 | 1,275 | 27 | 211 |
| Average | - | 6,458,097 | 89% | 647 | 1,807 | 66 | 332 |
| Total | 54 | 348,737,226 | 93% | 9,254 | 1,2168 | 497 | 1,596 |
Supplementary Figure S1

## Slide 4
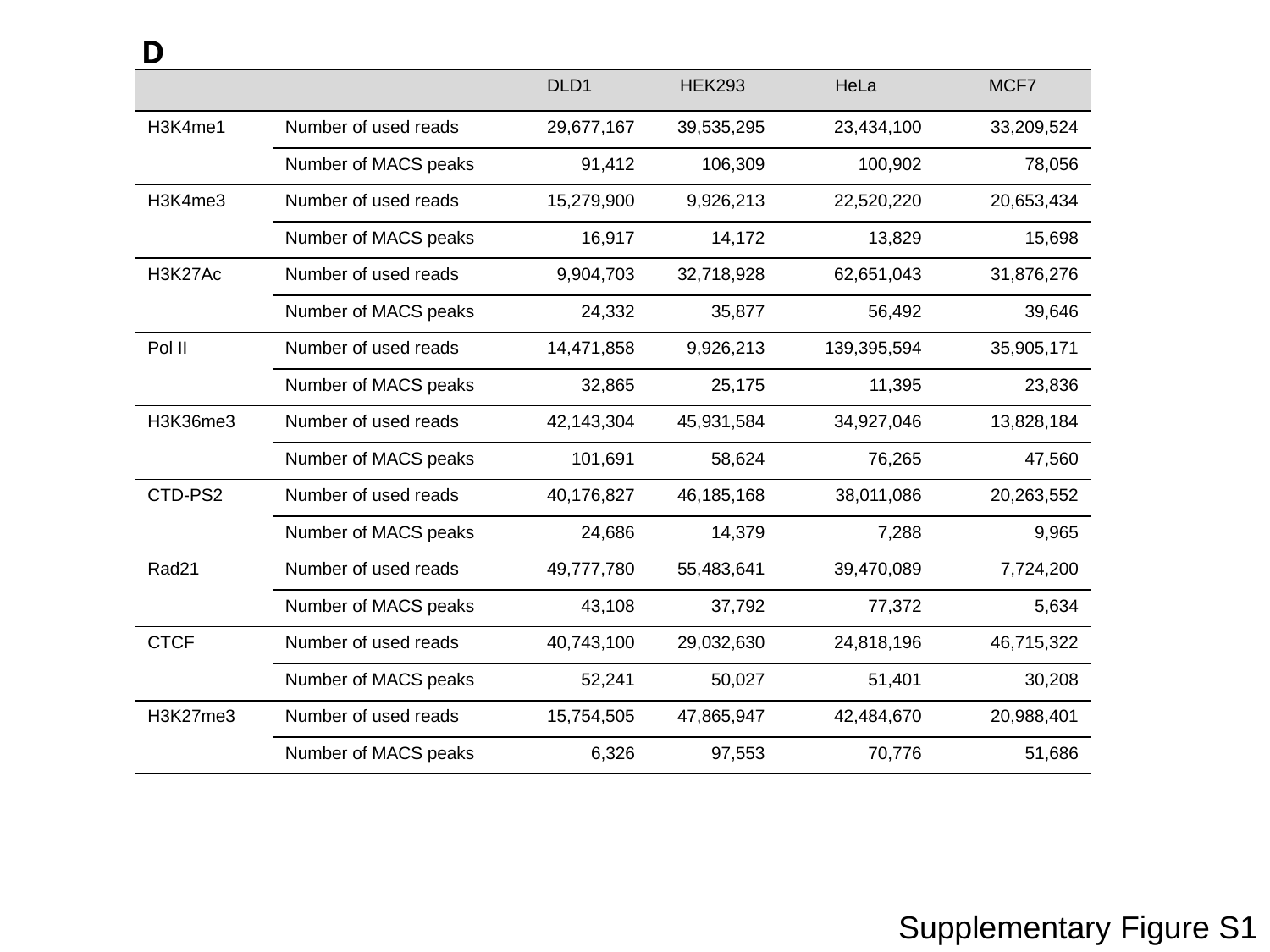

D
| | | DLD1 | HEK293 | HeLa | MCF7 |
| --- | --- | --- | --- | --- | --- |
| H3K4me1 | Number of used reads | 29,677,167 | 39,535,295 | 23,434,100 | 33,209,524 |
| | Number of MACS peaks | 91,412 | 106,309 | 100,902 | 78,056 |
| H3K4me3 | Number of used reads | 15,279,900 | 9,926,213 | 22,520,220 | 20,653,434 |
| | Number of MACS peaks | 16,917 | 14,172 | 13,829 | 15,698 |
| H3K27Ac | Number of used reads | 9,904,703 | 32,718,928 | 62,651,043 | 31,876,276 |
| | Number of MACS peaks | 24,332 | 35,877 | 56,492 | 39,646 |
| Pol II | Number of used reads | 14,471,858 | 9,926,213 | 139,395,594 | 35,905,171 |
| | Number of MACS peaks | 32,865 | 25,175 | 11,395 | 23,836 |
| H3K36me3 | Number of used reads | 42,143,304 | 45,931,584 | 34,927,046 | 13,828,184 |
| | Number of MACS peaks | 101,691 | 58,624 | 76,265 | 47,560 |
| CTD-PS2 | Number of used reads | 40,176,827 | 46,185,168 | 38,011,086 | 20,263,552 |
| | Number of MACS peaks | 24,686 | 14,379 | 7,288 | 9,965 |
| Rad21 | Number of used reads | 49,777,780 | 55,483,641 | 39,470,089 | 7,724,200 |
| | Number of MACS peaks | 43,108 | 37,792 | 77,372 | 5,634 |
| CTCF | Number of used reads | 40,743,100 | 29,032,630 | 24,818,196 | 46,715,322 |
| | Number of MACS peaks | 52,241 | 50,027 | 51,401 | 30,208 |
| H3K27me3 | Number of used reads | 15,754,505 | 47,865,947 | 42,484,670 | 20,988,401 |
| | Number of MACS peaks | 6,326 | 97,553 | 70,776 | 51,686 |
Supplementary Figure S1

## Slide 5
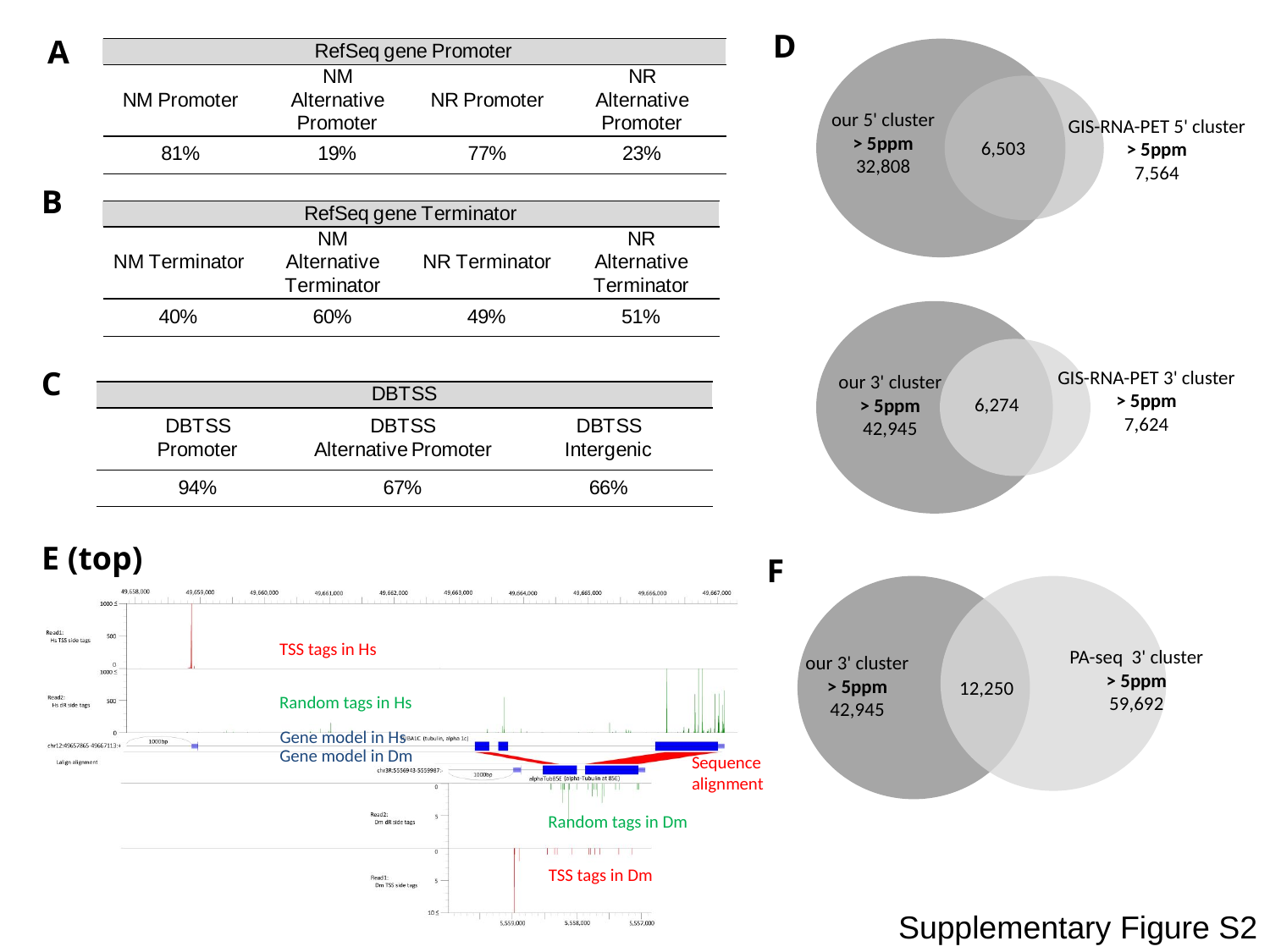

D
A
our 5' cluster
> 5ppm
32,808
GIS-RNA-PET 5' cluster
> 5ppm
7,564
6,503
B
C
GIS-RNA-PET 3' cluster
> 5ppm
7,624
our 3' cluster
> 5ppm
42,945
6,274
E (top)
F
TSS tags in Hs
PA-seq 3' cluster
> 5ppm
59,692
our 3' cluster
> 5ppm
42,945
12,250
Random tags in Hs
Gene model in Hs
Gene model in Dm
Sequence
alignment
Random tags in Dm
TSS tags in Dm
Supplementary Figure S2

## Slide 6
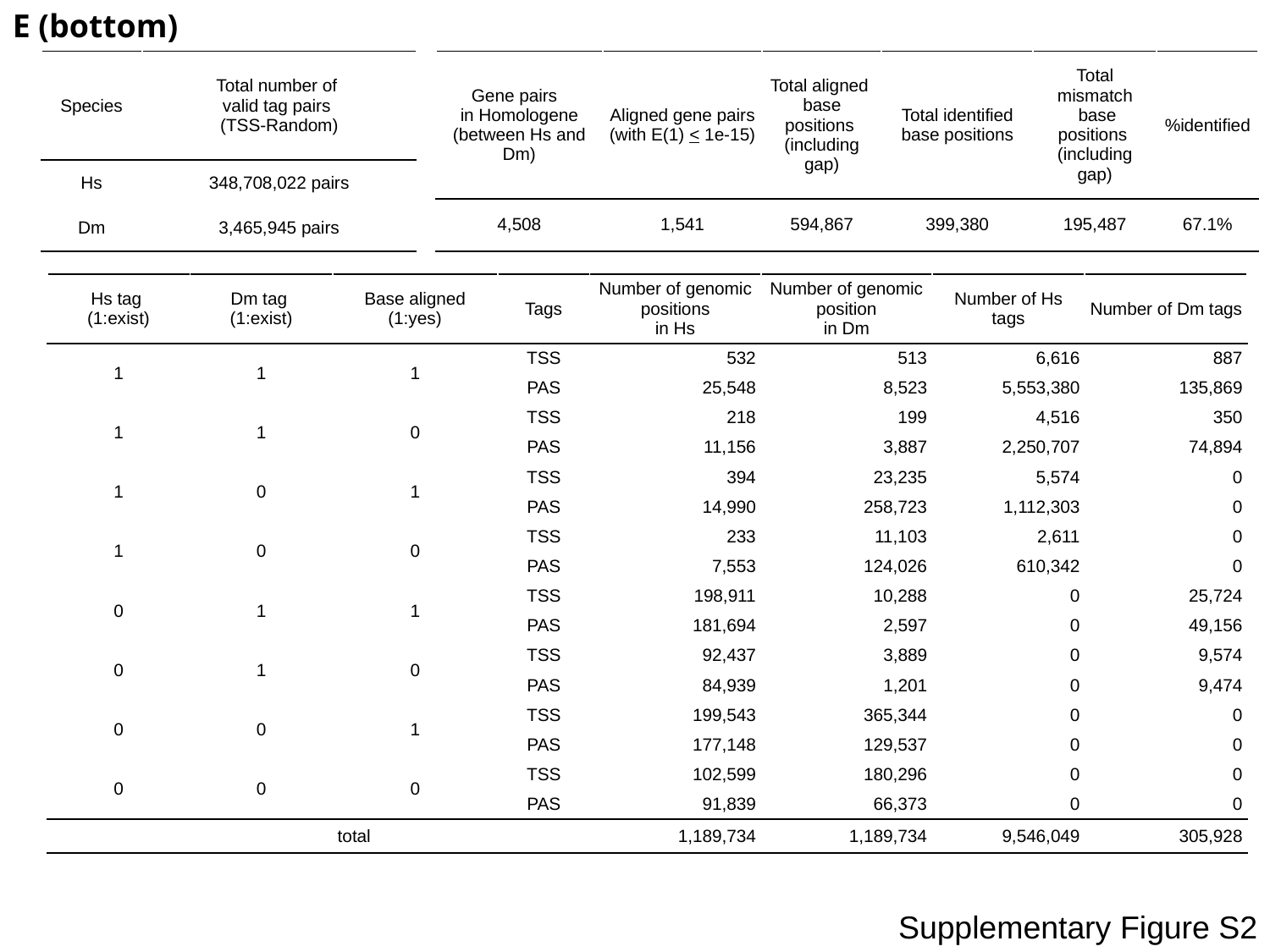

E (bottom)
| Species | Total number of valid tag pairs (TSS-Random) |
| --- | --- |
| Hs | 348,708,022 pairs |
| Dm | 3,465,945 pairs |
| Gene pairs in Homologene (between Hs and Dm) | Aligned gene pairs (with E(1) < 1e-15) | Total aligned base positions (including gap) | Total identified base positions | Total mismatch base positions (including gap) | %identified |
| --- | --- | --- | --- | --- | --- |
| 4,508 | 1,541 | 594,867 | 399,380 | 195,487 | 67.1% |
| Hs tag (1:exist) | Dm tag (1:exist) | Base aligned (1:yes) | Tags | Number of genomic positions in Hs | Number of genomic position in Dm | Number of Hs tags | Number of Dm tags |
| --- | --- | --- | --- | --- | --- | --- | --- |
| 1 | 1 | 1 | TSS | 532 | 513 | 6,616 | 887 |
| | | | PAS | 25,548 | 8,523 | 5,553,380 | 135,869 |
| 1 | 1 | 0 | TSS | 218 | 199 | 4,516 | 350 |
| | | | PAS | 11,156 | 3,887 | 2,250,707 | 74,894 |
| 1 | 0 | 1 | TSS | 394 | 23,235 | 5,574 | 0 |
| | | | PAS | 14,990 | 258,723 | 1,112,303 | 0 |
| 1 | 0 | 0 | TSS | 233 | 11,103 | 2,611 | 0 |
| | | | PAS | 7,553 | 124,026 | 610,342 | 0 |
| 0 | 1 | 1 | TSS | 198,911 | 10,288 | 0 | 25,724 |
| | | | PAS | 181,694 | 2,597 | 0 | 49,156 |
| 0 | 1 | 0 | TSS | 92,437 | 3,889 | 0 | 9,574 |
| | | | PAS | 84,939 | 1,201 | 0 | 9,474 |
| 0 | 0 | 1 | TSS | 199,543 | 365,344 | 0 | 0 |
| | | | PAS | 177,148 | 129,537 | 0 | 0 |
| 0 | 0 | 0 | TSS | 102,599 | 180,296 | 0 | 0 |
| | | | PAS | 91,839 | 66,373 | 0 | 0 |
| | | total | | 1,189,734 | 1,189,734 | 9,546,049 | 305,928 |
Supplementary Figure S2

## Slide 7
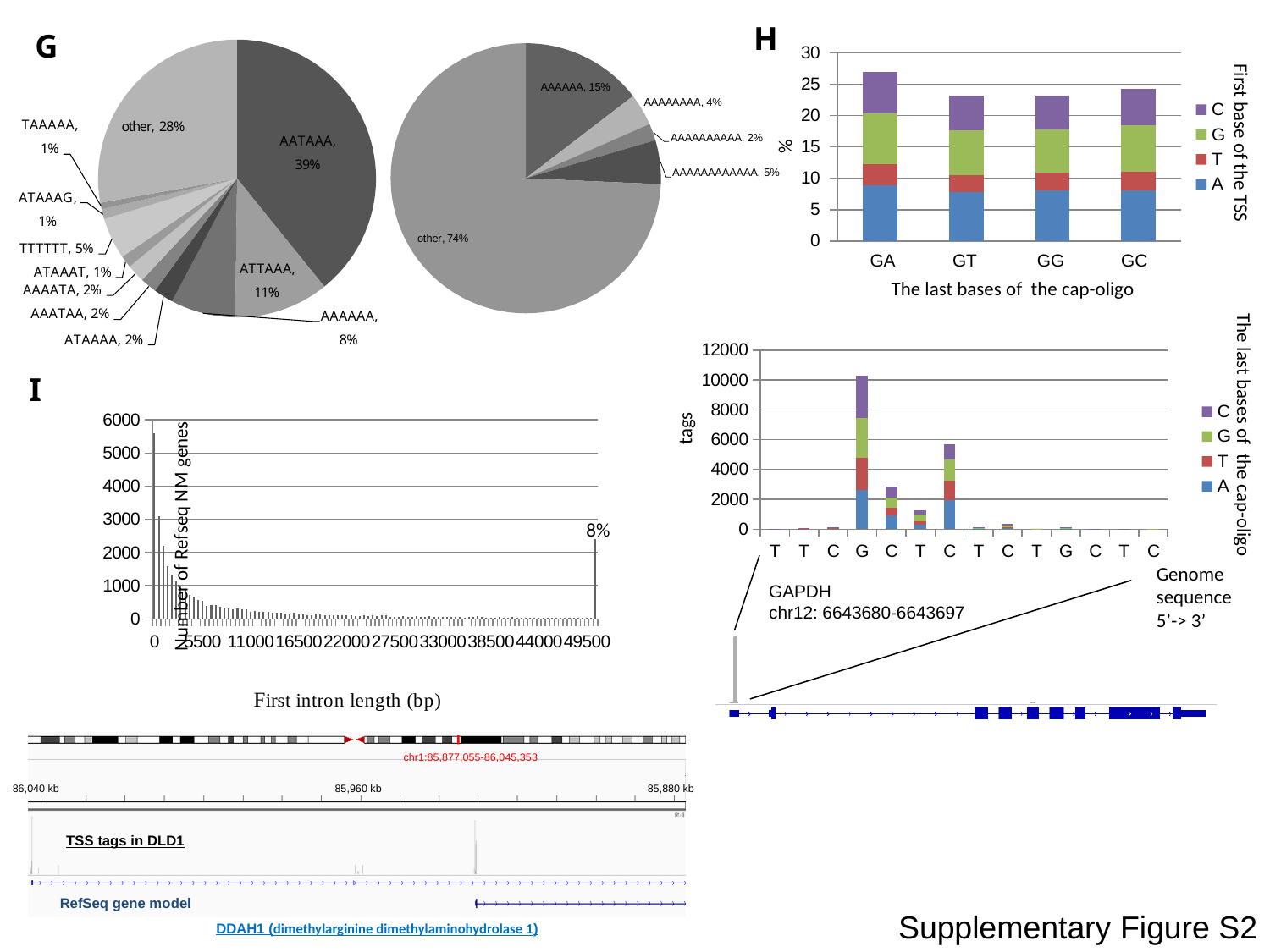

H
G
### Chart
| Category | | | | |
|---|---|---|---|---|GA
GT
GG
GC
%
First base of the TSS
The last bases of the cap-oligo
### Chart
| Category | A | T | G | C |
|---|---|---|---|---|
| T | 11.0 | 0.0 | 0.0 | 15.0 |
| T | 23.0 | 7.0 | 48.0 | 4.0 |
| C | 52.0 | 13.0 | 49.0 | 35.0 |
| G | 2614.0 | 2142.0 | 2714.0 | 2813.0 |
| C | 946.0 | 479.0 | 721.0 | 716.0 |
| T | 283.0 | 253.0 | 422.0 | 318.0 |
| C | 1959.0 | 1293.0 | 1436.0 | 1035.0 |
| T | 15.0 | 42.0 | 44.0 | 33.0 |
| C | 76.0 | 44.0 | 98.0 | 150.0 |
| T | 8.0 | 14.0 | 3.0 | 1.0 |
| G | 38.0 | 40.0 | 16.0 | 26.0 |
| C | 0.0 | 0.0 | 0.0 | 5.0 |
| T | 0.0 | 0.0 | 0.0 | 4.0 |
| C | 0.0 | 0.0 | 4.0 | 1.0 |GAPDH
chr12: 6643680-6643697
### Chart
| Category | |
|---|---|
| 0 | 5583.0 |
| 500 | 3092.0 |
| 1000 | 2199.0 |
| 1500 | 1599.0 |
| 2000 | 1331.0 |
| 2500 | 1122.0 |
| 3000 | 1013.0 |
| 3500 | 868.0 |
| 4000 | 734.0 |
| 4500 | 670.0 |
| 5000 | 567.0 |
| 5500 | 536.0 |
| 6000 | 406.0 |
| 6500 | 427.0 |
| 7000 | 426.0 |
| 7500 | 372.0 |
| 8000 | 322.0 |
| 8500 | 323.0 |
| 9000 | 288.0 |
| 9500 | 325.0 |
| 10000 | 291.0 |
| 10500 | 295.0 |
| 11000 | 222.0 |
| 11500 | 242.0 |
| 12000 | 227.0 |
| 12500 | 212.0 |
| 13000 | 209.0 |
| 13500 | 194.0 |
| 14000 | 186.0 |
| 14500 | 182.0 |
| 15000 | 175.0 |
| 15500 | 150.0 |
| 16000 | 180.0 |
| 16500 | 141.0 |
| 17000 | 136.0 |
| 17500 | 114.0 |
| 18000 | 119.0 |
| 18500 | 163.0 |
| 19000 | 131.0 |
| 19500 | 103.0 |
| 20000 | 113.0 |
| 20500 | 109.0 |
| 21000 | 109.0 |
| 21500 | 108.0 |
| 22000 | 106.0 |
| 22500 | 103.0 |
| 23000 | 96.0 |
| 23500 | 95.0 |
| 24000 | 105.0 |
| 24500 | 87.0 |
| 25000 | 105.0 |
| 25500 | 88.0 |
| 26000 | 113.0 |
| 26500 | 101.0 |
| 27000 | 73.0 |
| 27500 | 66.0 |
| 28000 | 73.0 |
| 28500 | 84.0 |
| 29000 | 56.0 |
| 29500 | 57.0 |
| 30000 | 82.0 |
| 30500 | 54.0 |
| 31000 | 50.0 |
| 31500 | 94.0 |
| 32000 | 59.0 |
| 32500 | 70.0 |
| 33000 | 51.0 |
| 33500 | 67.0 |
| 34000 | 54.0 |
| 34500 | 55.0 |
| 35000 | 57.0 |
| 35500 | 49.0 |
| 36000 | 58.0 |
| 36500 | 58.0 |
| 37000 | 77.0 |
| 37500 | 54.0 |
| 38000 | 42.0 |
| 38500 | 43.0 |
| 39000 | 47.0 |
| 39500 | 71.0 |
| 40000 | 44.0 |
| 40500 | 48.0 |
| 41000 | 59.0 |
| 41500 | 46.0 |
| 42000 | 39.0 |
| 42500 | 39.0 |
| 43000 | 31.0 |
| 43500 | 33.0 |
| 44000 | 29.0 |
| 44500 | 45.0 |
| 45000 | 40.0 |
| 45500 | 43.0 |
| 46000 | 29.0 |
| 46500 | 35.0 |
| 47000 | 41.0 |
| 47500 | 25.0 |
| 48000 | 29.0 |
| 48500 | 28.0 |
| 49000 | 29.0 |
| 49500 | 36.0 |
| 50000 | 37.0 |
| 50500 | 2416.0 |8%
I
tags
The last bases of the cap-oligo
Genome sequence
5’-> 3’
chr1:85,877,055-86,045,353
86,040 kb
85,960 kb
85,880 kb
TSS tags in DLD1
RefSeq gene model
Supplementary Figure S2
DDAH1 (dimethylarginine dimethylaminohydrolase 1)

## Slide 8
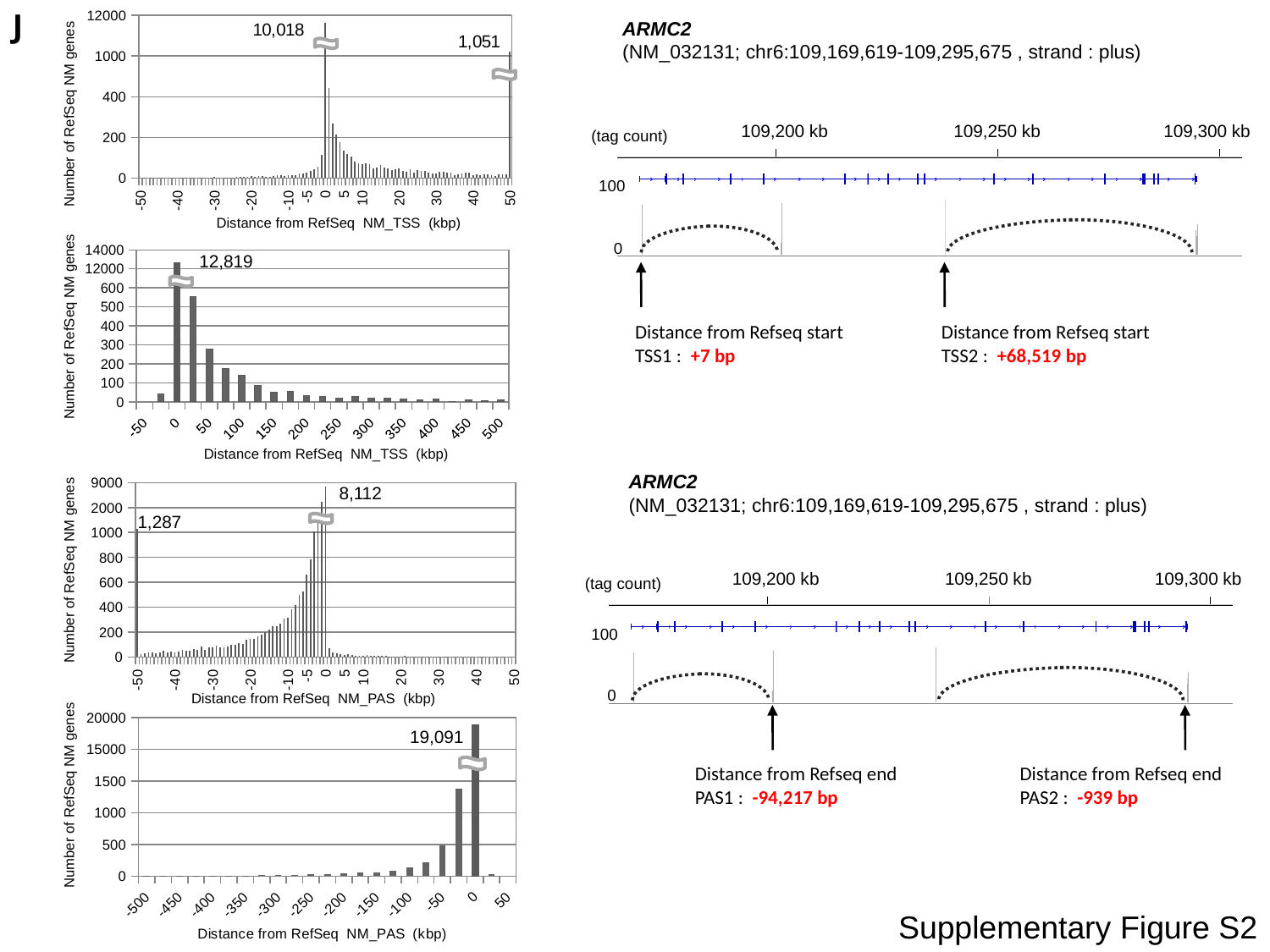

J
### Chart
| Category | | |
|---|---|---|
| -50 | None | None |
| | 3.0 | None |
| | None | None |
| | 1.0 | None |
| | 2.0 | None |
| | 2.0 | None |
| | 2.0 | None |
| | None | None |
| | 2.0 | None |
| | 1.0 | None |
| -40 | None | None |
| | 2.0 | None |
| | 3.0 | None |
| | None | None |
| | 1.0 | None |
| | None | None |
| | 2.0 | None |
| | 1.0 | None |
| | 2.0 | None |
| | 2.0 | None |
| -30 | 4.0 | None |
| | 3.0 | None |
| | None | None |
| | 3.0 | None |
| | 3.0 | None |
| | 2.0 | None |
| | 4.0 | None |
| | 6.0 | None |
| | 4.0 | None |
| | 5.0 | None |
| -20 | 8.0 | None |
| | 7.0 | None |
| | 10.0 | None |
| | 9.0 | None |
| | 6.0 | None |
| | 5.0 | None |
| | 10.0 | None |
| | 12.0 | None |
| | 13.0 | None |
| | 10.0 | None |
| -10 | 13.0 | None |
| | 13.0 | None |
| | 13.0 | None |
| | 21.0 | None |
| | 24.0 | None |
| -5 | 26.0 | None |
| | 36.0 | None |
| | 43.0 | None |
| | 56.0 | None |
| | 114.0 | None |
| 0 | None | 15118.0 |
| | 442.0 | None |
| | 266.0 | None |
| | 212.0 | None |
| | 176.0 | None |
| 5 | 133.0 | None |
| | 116.0 | None |
| | 105.0 | None |
| | 80.0 | None |
| | 73.0 | None |
| 10 | 67.0 | None |
| | 71.0 | None |
| | 66.0 | None |
| | 49.0 | None |
| | 52.0 | None |
| | 63.0 | None |
| | 50.0 | None |
| | 49.0 | None |
| | 37.0 | None |
| | 43.0 | None |
| 20 | 47.0 | None |
| | 35.0 | None |
| | 31.0 | None |
| | 43.0 | None |
| | 27.0 | None |
| | 39.0 | None |
| | 35.0 | None |
| | 34.0 | None |
| | 27.0 | None |
| | 24.0 | None |
| 30 | 21.0 | None |
| | 32.0 | None |
| | 30.0 | None |
| | 25.0 | None |
| | 27.0 | None |
| | 13.0 | None |
| | 16.0 | None |
| | 23.0 | None |
| | 25.0 | None |
| | 28.0 | None |
| 40 | 14.0 | None |
| | 17.0 | None |
| | 13.0 | None |
| | 16.0 | None |
| | 20.0 | None |
| | 14.0 | None |
| | 10.0 | None |
| | 19.0 | None |
| | 17.0 | None |
| | 16.0 | None |
| 50 | None | 14800.0 |ARMC2
(NM_032131; chr6:109,169,619-109,295,675 , strand : plus)
Number of RefSeq NM genes
109,300 kb
109,200 kb
109,250 kb
(tag count)
100
Distance from RefSeq NM_TSS (kbp)
### Chart
| Category | | |
|---|---|---|
| -50 | None | None |
| -25 | 41.0 | None |
| 0 | None | 12819.0 |
| 25 | 555.0 | None |
| 50 | 279.0 | None |
| 75 | 176.0 | None |
| 100 | 138.0 | None |
| 125 | 85.0 | None |
| 150 | 50.0 | None |
| 175 | 55.0 | None |
| 200 | 31.0 | None |
| 225 | 26.0 | None |
| 250 | 17.0 | None |
| 275 | 27.0 | None |
| 300 | 19.0 | None |
| 325 | 20.0 | None |
| 350 | 13.0 | None |
| 375 | 9.0 | None |
| 400 | 15.0 | None |
| 425 | 3.0 | None |
| 450 | 9.0 | None |
| 475 | 5.0 | None |
| 500 | 10.0 | None |
12,819
0
Number of RefSeq NM genes
Distance from Refseq start
TSS1 : +7 bp
Distance from Refseq start
TSS2 : +68,519 bp
Distance from RefSeq NM_TSS (kbp)
### Chart
| Category | | |
|---|---|---|
| -50 | None | 6600.0 |
| | 26.0 | None |
| | 31.0 | None |
| | 36.0 | None |
| | 33.0 | None |
| | 31.0 | None |
| | 33.0 | None |
| | 48.0 | None |
| | 35.0 | None |
| | 40.0 | None |
| -40 | 36.0 | None |
| | 41.0 | None |
| | 55.0 | None |
| | 47.0 | None |
| | 50.0 | None |
| | 65.0 | None |
| | 54.0 | None |
| | 83.0 | None |
| | 58.0 | None |
| | 76.0 | None |
| -30 | 77.0 | None |
| | 94.0 | None |
| | 76.0 | None |
| | 77.0 | None |
| | 84.0 | None |
| | 98.0 | None |
| | 99.0 | None |
| | 110.0 | None |
| | 106.0 | None |
| | 135.0 | None |
| -20 | 146.0 | None |
| | 142.0 | None |
| | 166.0 | None |
| | 180.0 | None |
| | 202.0 | None |
| | 217.0 | None |
| | 247.0 | None |
| | 246.0 | None |
| | 270.0 | None |
| | 307.0 | None |
| -10 | 315.0 | None |
| | 383.0 | None |
| | 414.0 | None |
| | 498.0 | None |
| | 526.0 | None |
| -5 | 662.0 | None |
| | 781.0 | None |
| | None | 6500.0 |
| | None | 7000.0 |
| | None | 8000.0 |
| 0 | None | 8812.0 |
| | 71.0 | None |
| | 33.0 | None |
| | 30.0 | None |
| | 20.0 | None |
| 5 | 15.0 | None |
| | 25.0 | None |
| | 13.0 | None |
| | 12.0 | None |
| | 10.0 | None |
| 10 | 10.0 | None |
| | 14.0 | None |
| | 9.0 | None |
| | 11.0 | None |
| | 7.0 | None |
| | 7.0 | None |
| | 7.0 | None |
| | 5.0 | None |
| | 3.0 | None |
| | 5.0 | None |
| 20 | 2.0 | None |
| | 7.0 | None |
| | 5.0 | None |
| | 5.0 | None |
| | 2.0 | None |
| | 2.0 | None |
| | 4.0 | None |
| | 3.0 | None |
| | 2.0 | None |
| | 1.0 | None |
| 30 | 1.0 | None |
| | 2.0 | None |
| | 2.0 | None |
| | None | None |
| | 3.0 | None |
| | 1.0 | None |
| | 3.0 | None |
| | 3.0 | None |
| | 1.0 | None |
| | 1.0 | None |
| 40 | None | None |
| | 2.0 | None |
| | 3.0 | None |
| | None | None |
| | None | None |
| | 2.0 | None |
| | None | None |
| | 1.0 | None |
| | None | None |
| | 1.0 | None |
| 50 | None | None |8,112
1,287
Number of RefSeq NM genes
ARMC2
(NM_032131; chr6:109,169,619-109,295,675 , strand : plus)
109,300 kb
109,200 kb
109,250 kb
(tag count)
100
0
### Chart
| Category | | |
|---|---|---|
| -500 | 3.0 | None |
| -475 | 5.0 | None |
| -450 | 9.0 | None |
| -425 | 8.0 | None |
| -400 | 6.0 | None |
| -375 | 9.0 | None |
| -350 | 9.0 | None |
| -325 | 16.0 | None |
| -300 | 15.0 | None |
| -275 | 16.0 | None |
| -250 | 28.0 | None |
| -225 | 32.0 | None |
| -200 | 46.0 | None |
| -175 | 52.0 | None |
| -150 | 58.0 | None |
| -125 | 84.0 | None |
| -100 | 134.0 | None |
| -75 | 225.0 | None |
| -50 | 491.0 | None |
| -25 | 1384.0 | None |
| 0 | None | 19091.0 |
| 25 | 38.0 | None |
| 50 | None | None |
Number of RefSeq NM genes
Distance from RefSeq NM_PAS (kbp)
19,091
Distance from Refseq end
PAS1 : -94,217 bp
Distance from Refseq end
PAS2 : -939 bp
Supplementary Figure S2

## Slide 9
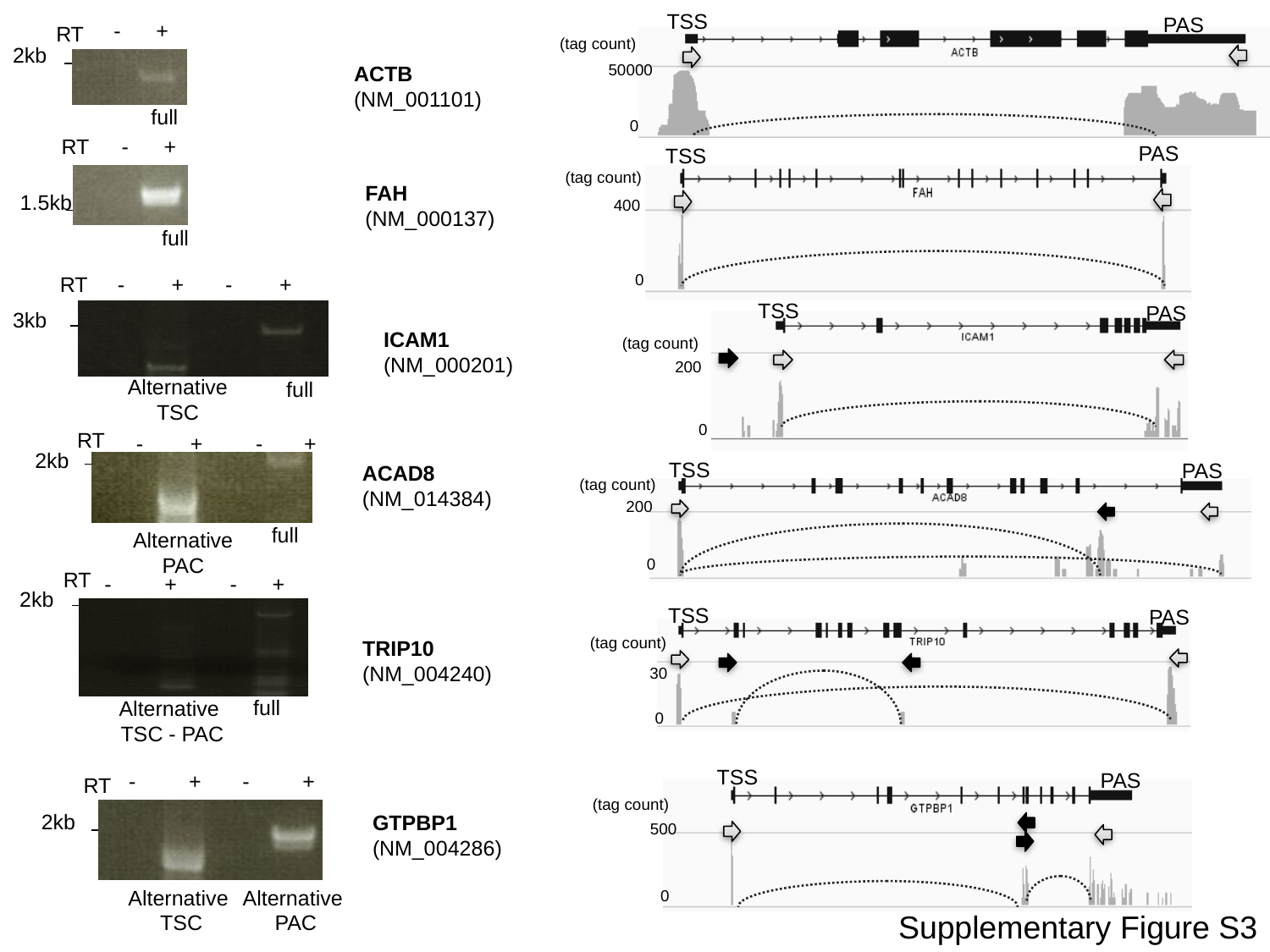

TSS
PAS
- +
RT
(tag count)
2kb
50000
ACTB
(NM_001101)
full
0
RT
- +
PAS
TSS
(tag count)
FAH
(NM_000137)
1.5kb
400
full
0
RT
- + - +
TSS
PAS
3kb
ICAM1
(NM_000201)
(tag count)
200
Alternative
TSC
full
0
RT
- + - +
2kb
TSS
PAS
ACAD8
(NM_014384)
(tag count)
200
full
Alternative
PAC
0
RT
- + - +
2kb
TSS
PAS
(tag count)
TRIP10
(NM_004240)
30
full
Alternative
TSC - PAC
0
TSS
PAS
- + - +
RT
(tag count)
2kb
GTPBP1
(NM_004286)
500
Alternative
TSC
Alternative
 PAC
0
Supplementary Figure S3

## Slide 10
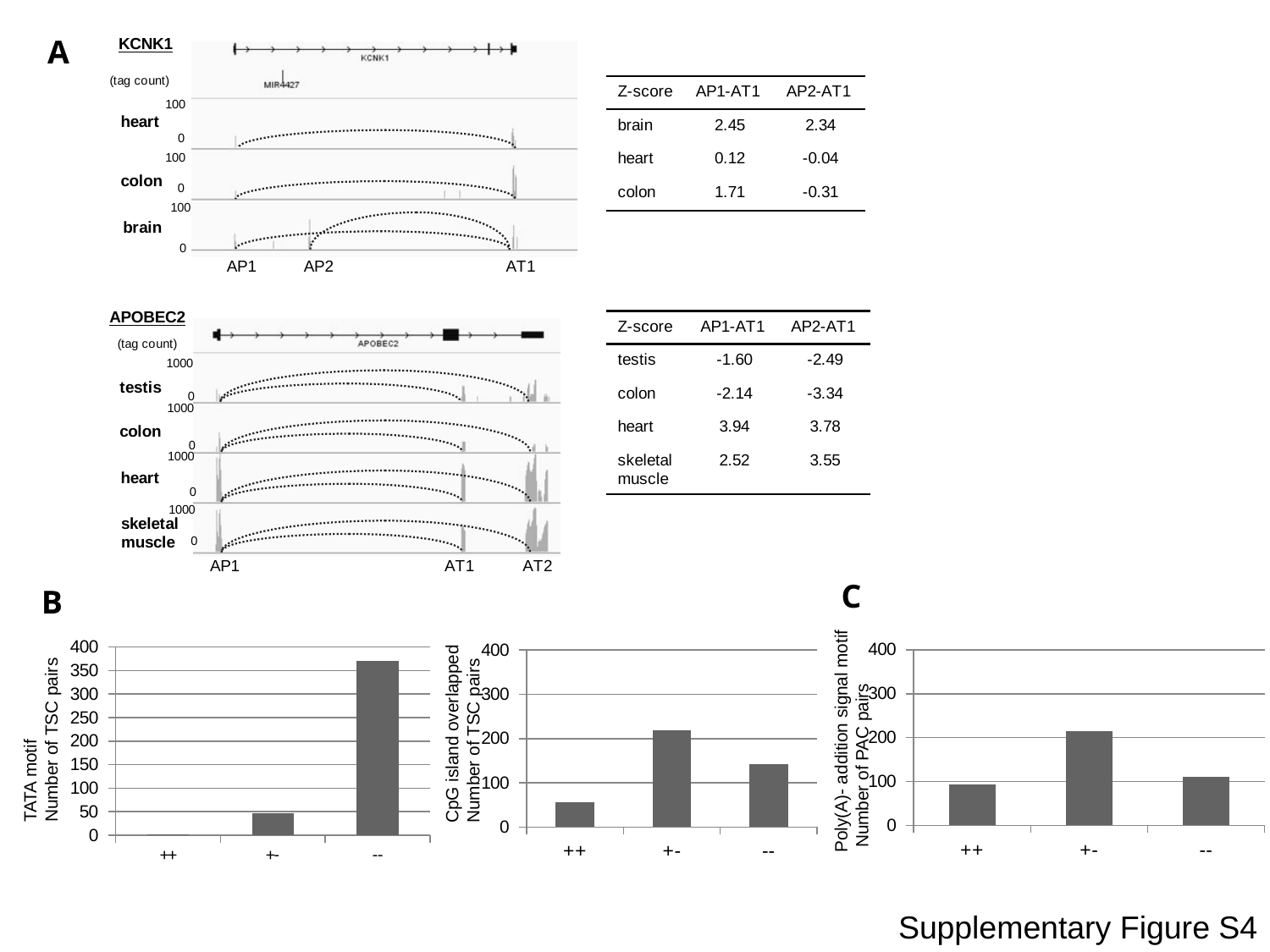

A
C
B
### Chart
| Category | |
|---|---|
| ++ | 1.0 |
| +- | 47.0 |
| -- | 371.0 |
### Chart
| Category | |
|---|---|
| ++ | 94.0 |
| +- | 215.0 |
| -- | 110.0 |
### Chart
| Category | |
|---|---|
| ++ | 57.0 |
| +- | 219.0 |
| -- | 143.0 |CpG island overlapped
Number of TSC pairs
TATA motif
Number of TSC pairs
Poly(A)- addition signal motif
 Number of PAC pairs
Supplementary Figure S4

## Slide 11
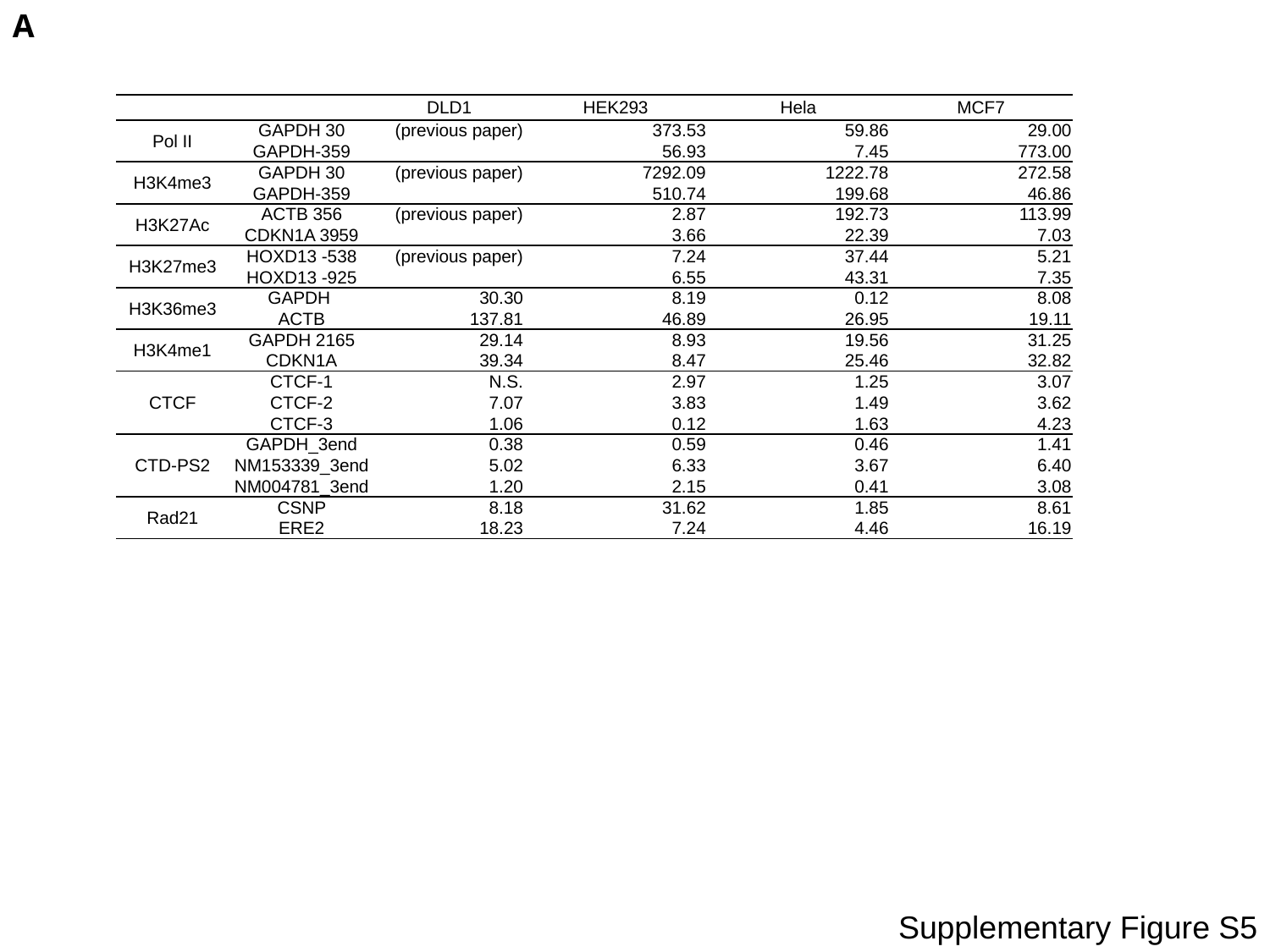

A
| | | DLD1 | HEK293 | Hela | MCF7 |
| --- | --- | --- | --- | --- | --- |
| Pol II | GAPDH 30 | (previous paper) | 373.53 | 59.86 | 29.00 |
| | GAPDH-359 | | 56.93 | 7.45 | 773.00 |
| H3K4me3 | GAPDH 30 | (previous paper) | 7292.09 | 1222.78 | 272.58 |
| | GAPDH-359 | | 510.74 | 199.68 | 46.86 |
| H3K27Ac | ACTB 356 | (previous paper) | 2.87 | 192.73 | 113.99 |
| | CDKN1A 3959 | | 3.66 | 22.39 | 7.03 |
| H3K27me3 | HOXD13 -538 | (previous paper) | 7.24 | 37.44 | 5.21 |
| | HOXD13 -925 | | 6.55 | 43.31 | 7.35 |
| H3K36me3 | GAPDH | 30.30 | 8.19 | 0.12 | 8.08 |
| | ACTB | 137.81 | 46.89 | 26.95 | 19.11 |
| H3K4me1 | GAPDH 2165 | 29.14 | 8.93 | 19.56 | 31.25 |
| | CDKN1A | 39.34 | 8.47 | 25.46 | 32.82 |
| CTCF | CTCF-1 | N.S. | 2.97 | 1.25 | 3.07 |
| | CTCF-2 | 7.07 | 3.83 | 1.49 | 3.62 |
| | CTCF-3 | 1.06 | 0.12 | 1.63 | 4.23 |
| CTD-PS2 | GAPDH\_3end | 0.38 | 0.59 | 0.46 | 1.41 |
| | NM153339\_3end | 5.02 | 6.33 | 3.67 | 6.40 |
| | NM004781\_3end | 1.20 | 2.15 | 0.41 | 3.08 |
| Rad21 | CSNP | 8.18 | 31.62 | 1.85 | 8.61 |
| | ERE2 | 18.23 | 7.24 | 4.46 | 16.19 |
Supplementary Figure S5

## Slide 12
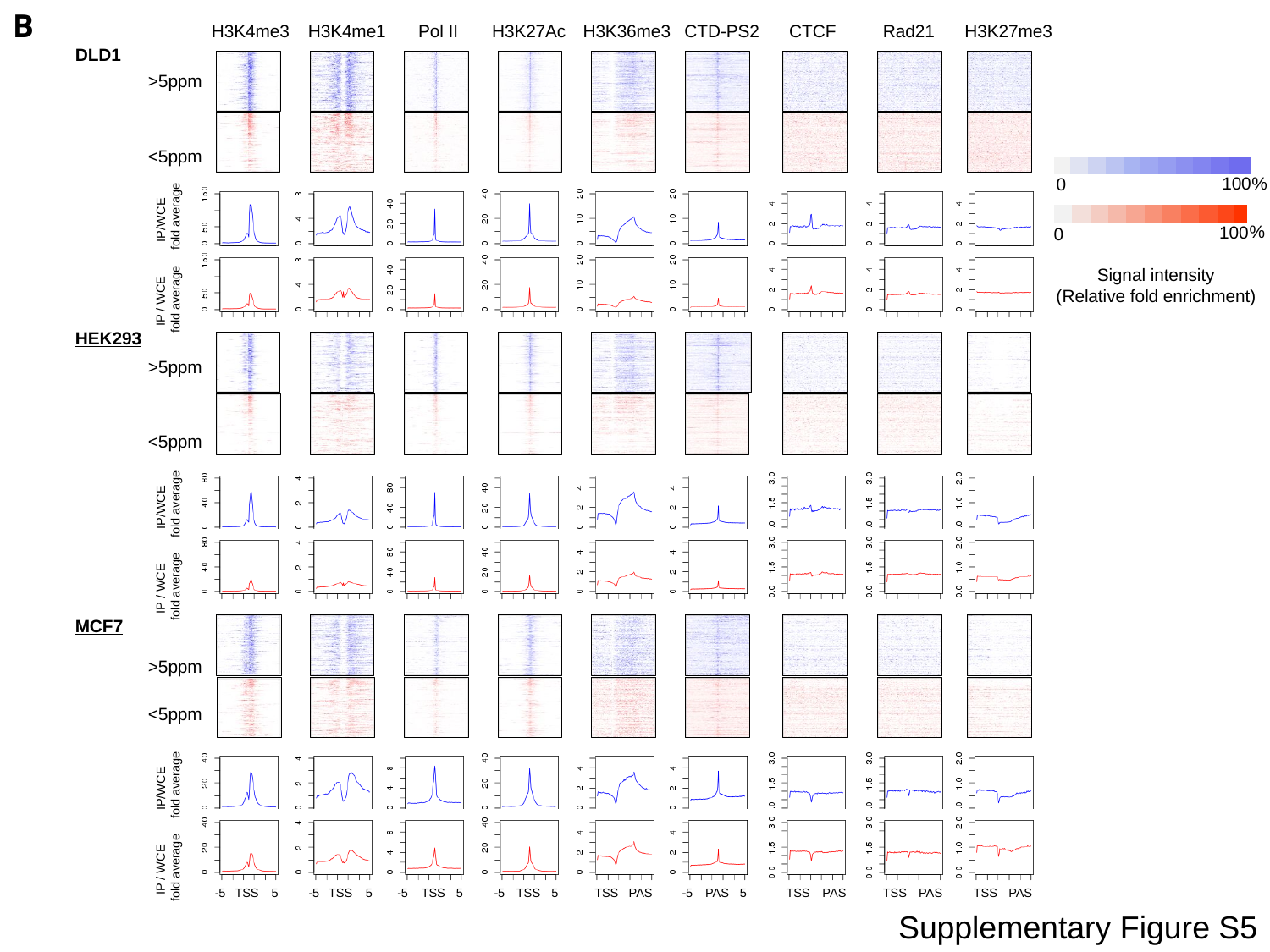

B
H3K4me3
H3K4me1
Pol II
H3K27Ac
H3K36me3
CTD-PS2
CTCF
Rad21
H3K27me3
DLD1
>5ppm
<5ppm
| | | | | | | | | | | |
| --- | --- | --- | --- | --- | --- | --- | --- | --- | --- | --- |
100
%
Signal intensity
(Relative fold enrichment)
0
IP/WCE
fold average
| | | | | | | | | | | |
| --- | --- | --- | --- | --- | --- | --- | --- | --- | --- | --- |
%
100
0
IP / WCE
 fold average
HEK293
>5ppm
<5ppm
IP/WCE
fold average
IP / WCE
 fold average
MCF7
>5ppm
<5ppm
IP/WCE
fold average
IP / WCE
 fold average
-5
TSS
5
-5
TSS
5
-5
TSS
5
-5
TSS
5
TSS
PAS
-5
PAS
5
TSS
PAS
TSS
PAS
TSS
PAS
Supplementary Figure S5

## Slide 13
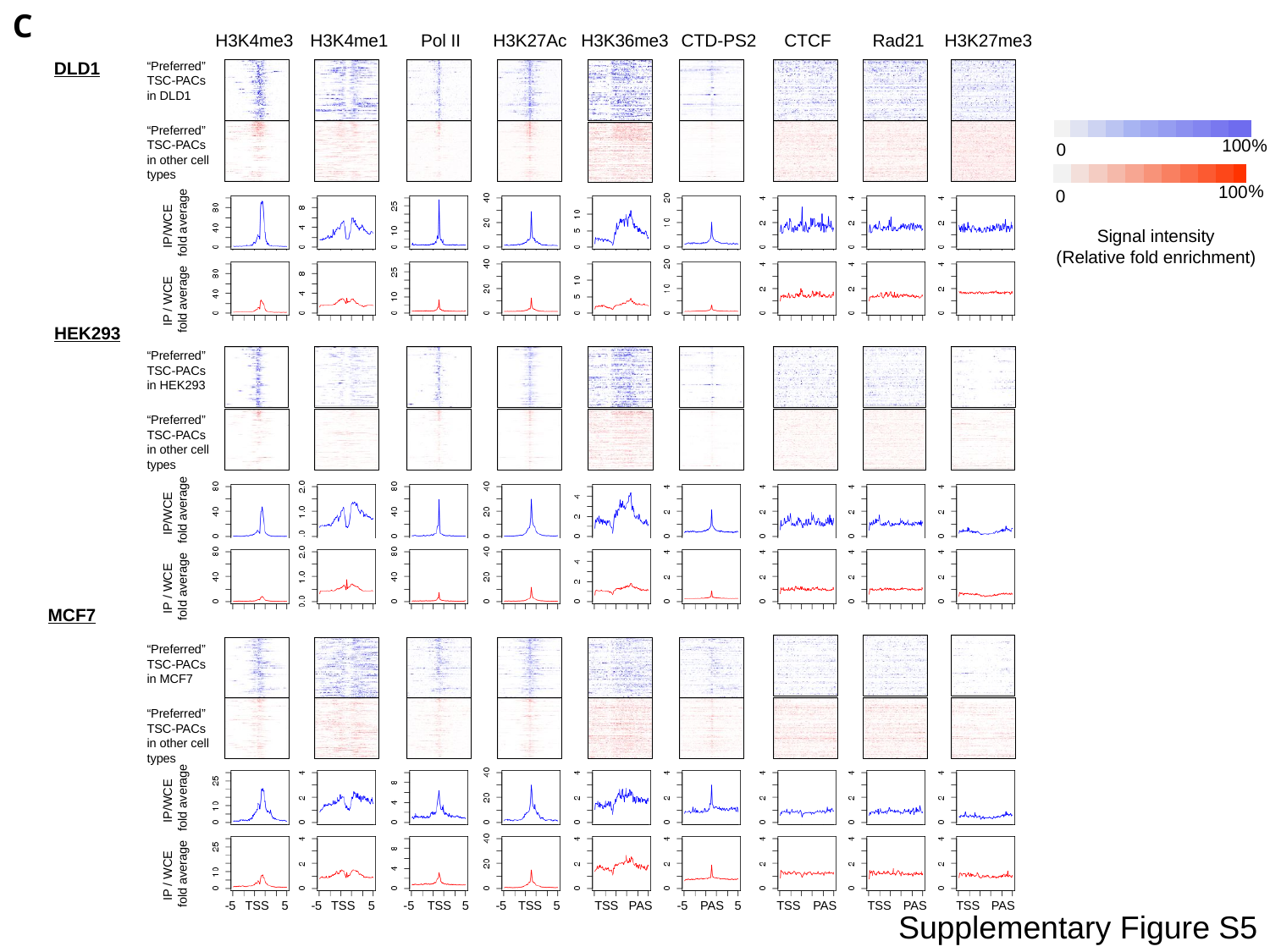

C
H3K4me3
H3K4me1
Pol II
H3K27Ac
H3K36me3
CTD-PS2
CTCF
Rad21
H3K27me3
DLD1
“Preferred” TSC-PACs
in DLD1
“Preferred” TSC-PACs
in other cell types
| | | | | | | | | | | |
| --- | --- | --- | --- | --- | --- | --- | --- | --- | --- | --- |
100
%
Signal intensity
(Relative fold enrichment)
0
| | | | | | | | | | | |
| --- | --- | --- | --- | --- | --- | --- | --- | --- | --- | --- |
%
100
0
IP/WCE
fold average
IP / WCE
 fold average
HEK293
“Preferred” TSC-PACs
in HEK293
“Preferred” TSC-PACs
in other cell types
IP/WCE
fold average
IP / WCE
 fold average
MCF7
“Preferred” TSC-PACs
in MCF7
“Preferred” TSC-PACs
in other cell types
IP/WCE
fold average
IP / WCE
 fold average
-5
TSS
5
-5
TSS
5
-5
TSS
5
-5
TSS
5
TSS
PAS
-5
PAS
5
TSS
PAS
TSS
PAS
TSS
PAS
Supplementary Figure S5

## Slide 14
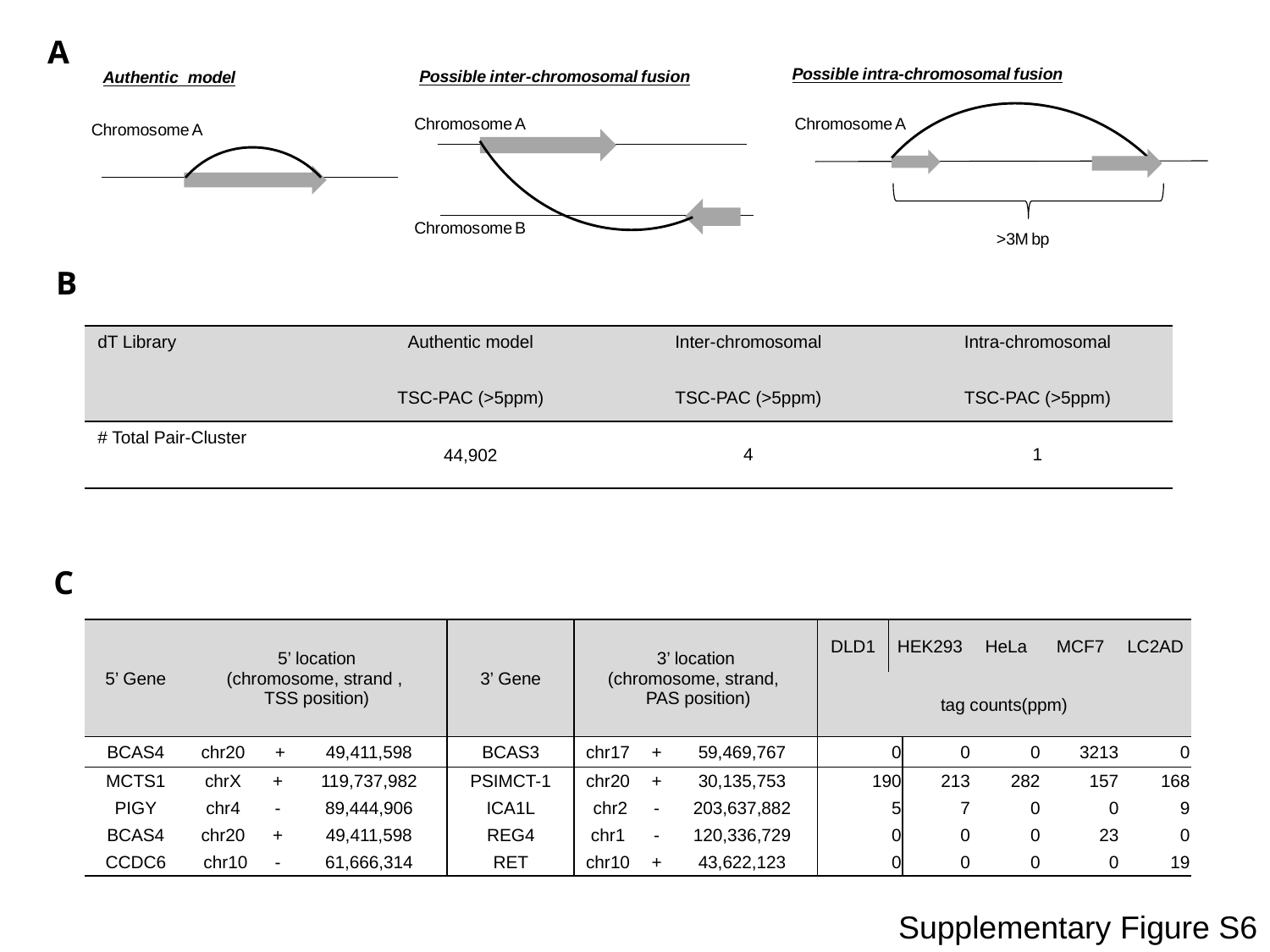

A
B
| dT Library | Authentic model | Inter-chromosomal | Intra-chromosomal |
| --- | --- | --- | --- |
| | TSC-PAC (>5ppm) | TSC-PAC (>5ppm) | TSC-PAC (>5ppm) |
| # Total Pair-Cluster | 44,902 | 4 | 1 |
C
| 5’ Gene | 5’ location (chromosome, strand , TSS position) | | | 3’ Gene | 3’ location (chromosome, strand, PAS position) | | | DLD1 | HEK293 | | HeLa | MCF7 | LC2AD |
| --- | --- | --- | --- | --- | --- | --- | --- | --- | --- | --- | --- | --- | --- |
| | | | | | | | | tag counts(ppm) | | | | | |
| BCAS4 | chr20 | + | 49,411,598 | BCAS3 | chr17 | + | 59,469,767 | 0 | | 0 | 0 | 3213 | 0 |
| MCTS1 | chrX | + | 119,737,982 | PSIMCT-1 | chr20 | + | 30,135,753 | 190 | | 213 | 282 | 157 | 168 |
| PIGY | chr4 | - | 89,444,906 | ICA1L | chr2 | - | 203,637,882 | 5 | | 7 | 0 | 0 | 9 |
| BCAS4 | chr20 | + | 49,411,598 | REG4 | chr1 | - | 120,336,729 | 0 | | 0 | 0 | 23 | 0 |
| CCDC6 | chr10 | - | 61,666,314 | RET | chr10 | + | 43,622,123 | 0 | | 0 | 0 | 0 | 19 |
Supplementary Figure S6

## Slide 15
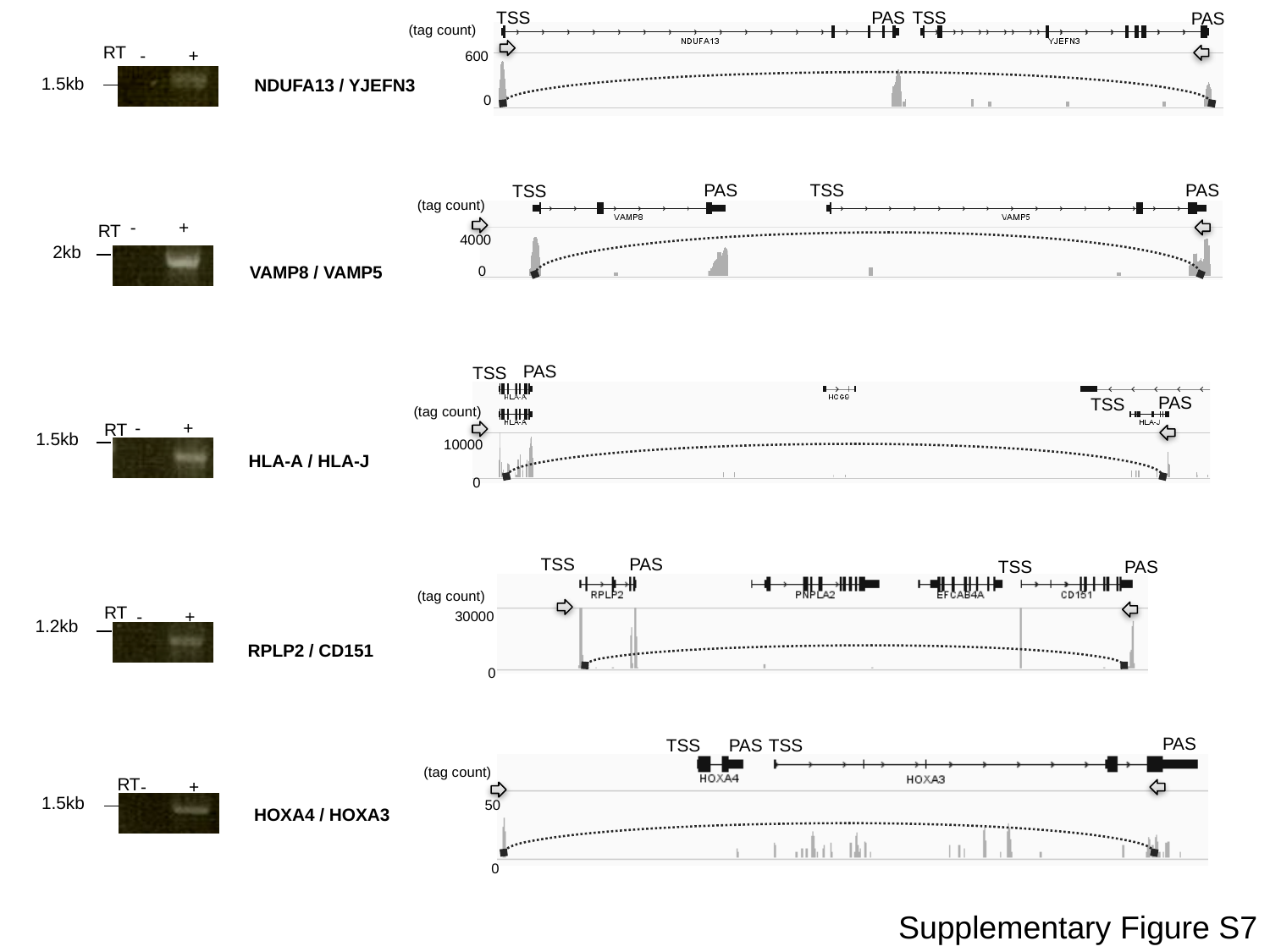

TSS
PAS
TSS
PAS
(tag count)
600
RT
-
+
1.5kb
NDUFA13 / YJEFN3
0
PAS
TSS
PAS
TSS
(tag count)
4000
-
+
2kb
VAMP8 / VAMP5
RT
0
PAS
TSS
10000
PAS
TSS
(tag count)
-
+
1.5kb
HLA-A / HLA-J
RT
0
TSS
PAS
TSS
PAS
30000
(tag count)
RT
-
+
1.2kb
RPLP2 / CD151
0
PAS
TSS
TSS
PAS
50
(tag count)
RT
-
+
1.5kb
HOXA4 / HOXA3
0
Supplementary Figure S7

## Slide 16
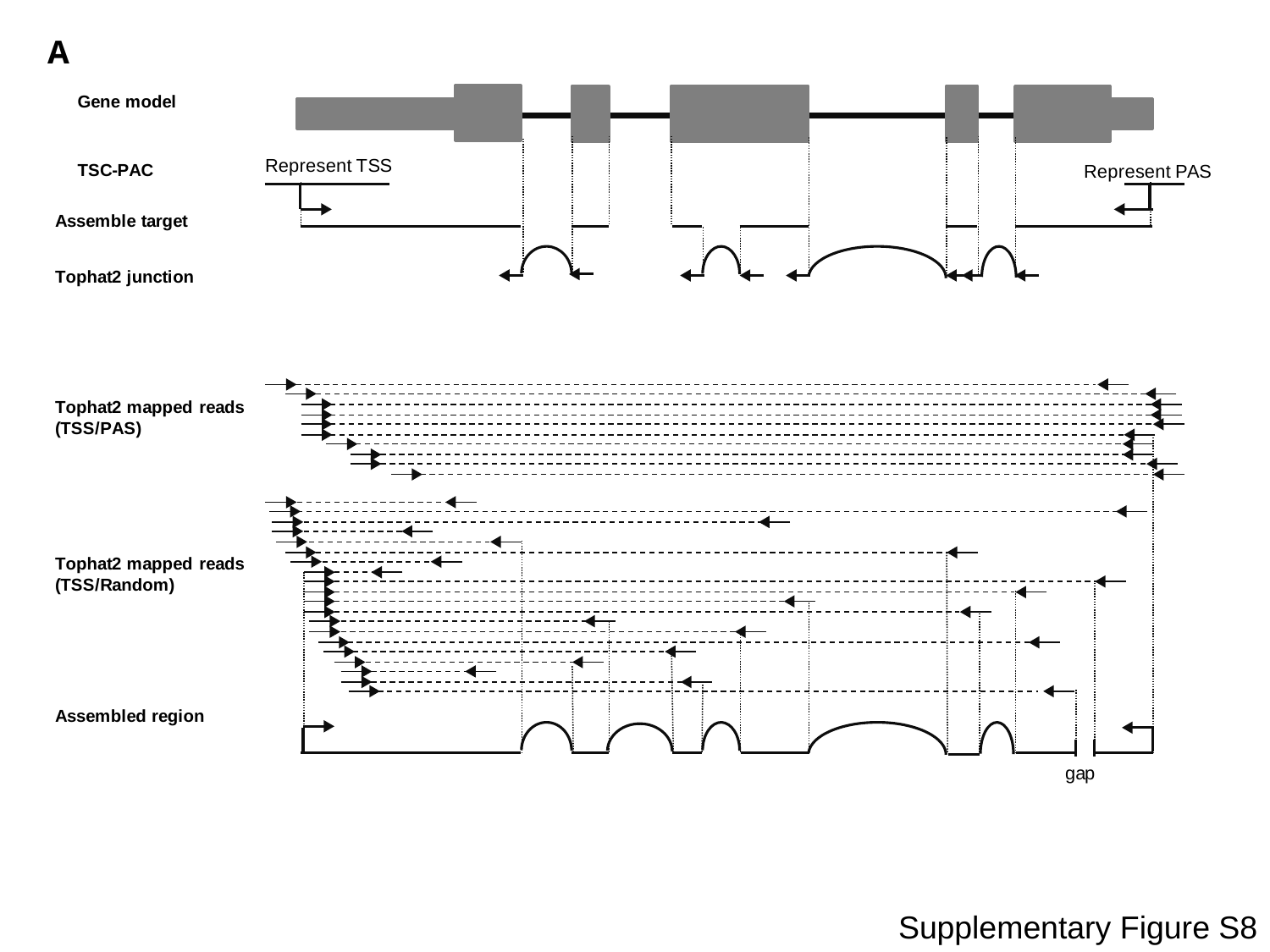

A
Supplementary Figure S8

## Slide 17
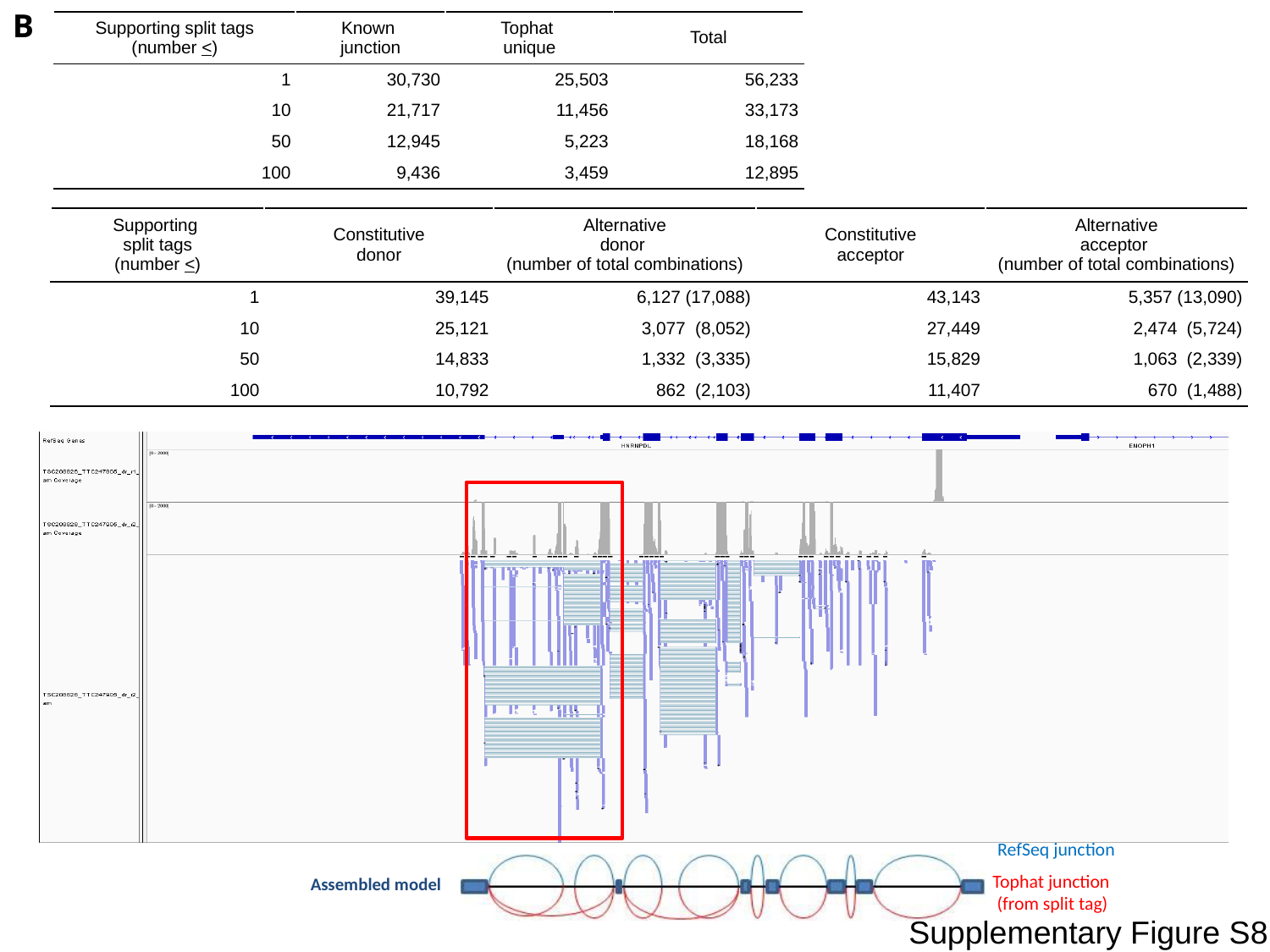

B
| Supporting split tags (number <) | Known junction | Tophat unique | Total |
| --- | --- | --- | --- |
| 1 | 30,730 | 25,503 | 56,233 |
| 10 | 21,717 | 11,456 | 33,173 |
| 50 | 12,945 | 5,223 | 18,168 |
| 100 | 9,436 | 3,459 | 12,895 |
| Supporting split tags (number <) | Constitutive donor | Alternative donor (number of total combinations) | Constitutive acceptor | Alternative acceptor (number of total combinations) |
| --- | --- | --- | --- | --- |
| 1 | 39,145 | 6,127 (17,088) | 43,143 | 5,357 (13,090) |
| 10 | 25,121 | 3,077 (8,052) | 27,449 | 2,474 (5,724) |
| 50 | 14,833 | 1,332 (3,335) | 15,829 | 1,063 (2,339) |
| 100 | 10,792 | 862 (2,103) | 11,407 | 670 (1,488) |
RefSeq junction
Tophat junction
 (from split tag)
Assembled model
Supplementary Figure S8

## Slide 18
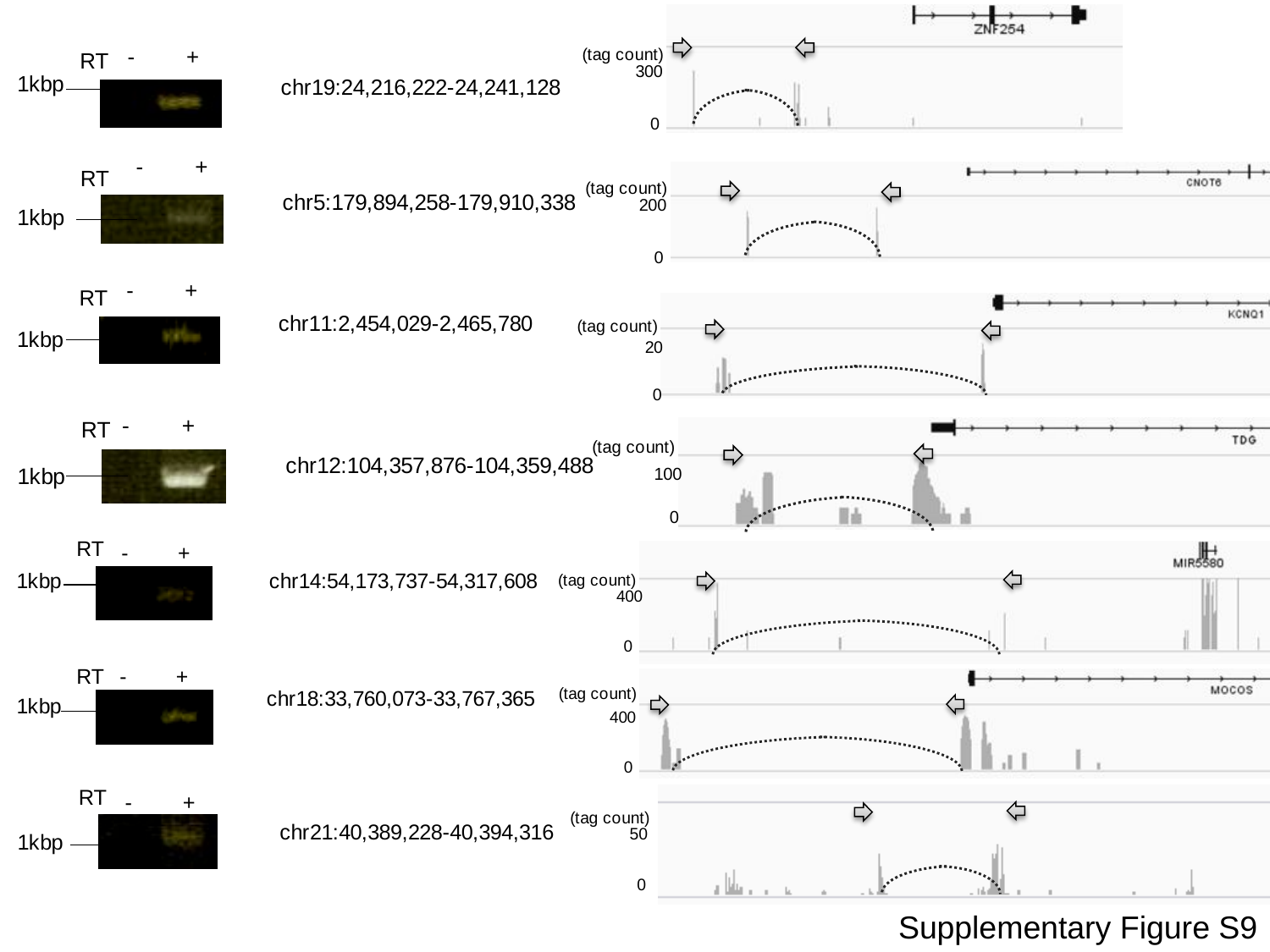

Supplementary Figure S9

## Slide 19
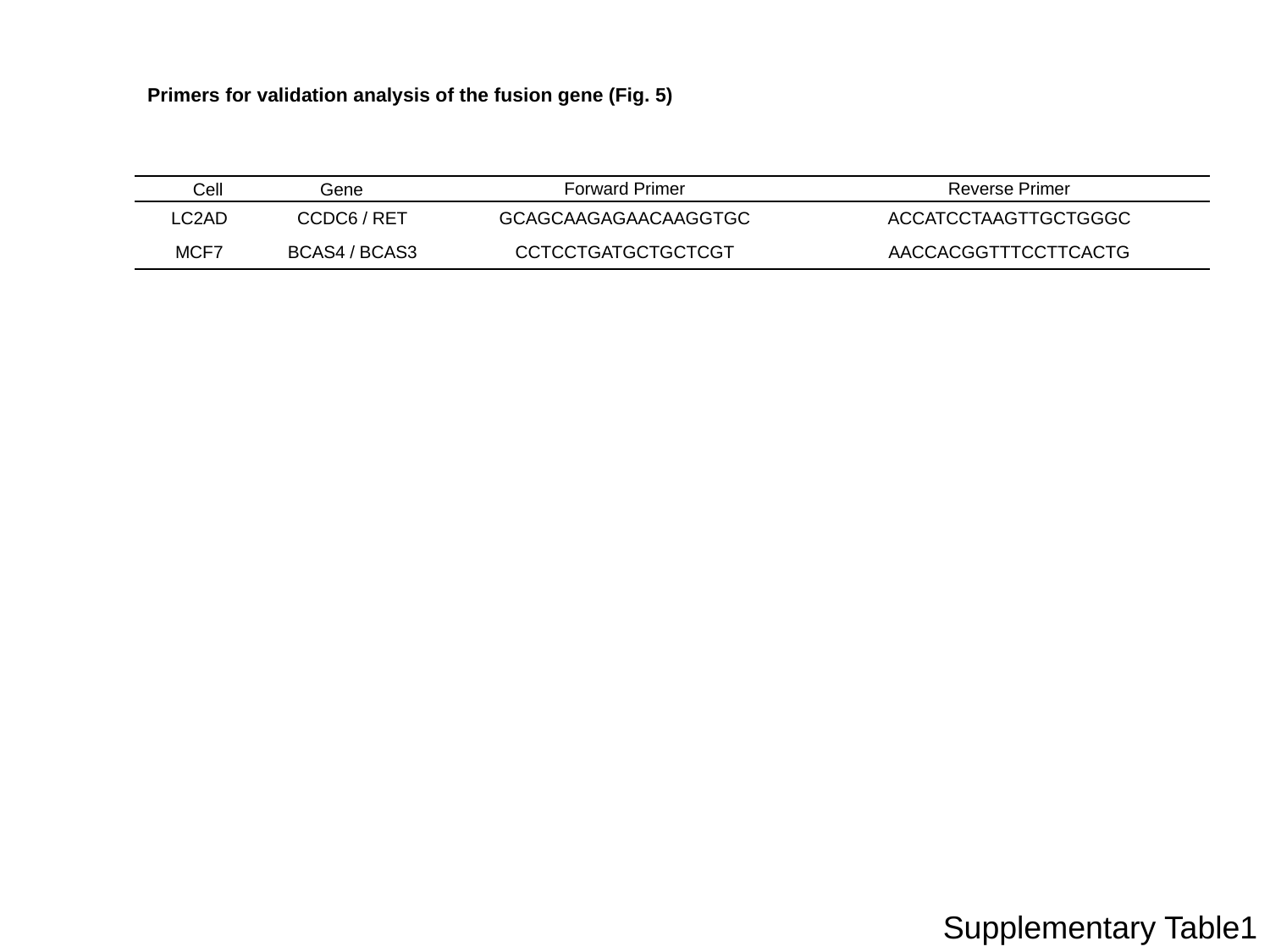

Primers for validation analysis of the fusion gene (Fig. 5)
| Cell | Gene | Forward Primer | Reverse Primer |
| --- | --- | --- | --- |
| LC2AD | CCDC6 / RET | GCAGCAAGAGAACAAGGTGC | ACCATCCTAAGTTGCTGGGC |
| MCF7 | BCAS4 / BCAS3 | CCTCCTGATGCTGCTCGT | AACCACGGTTTCCTTCACTG |
Supplementary Table1

## Slide 20
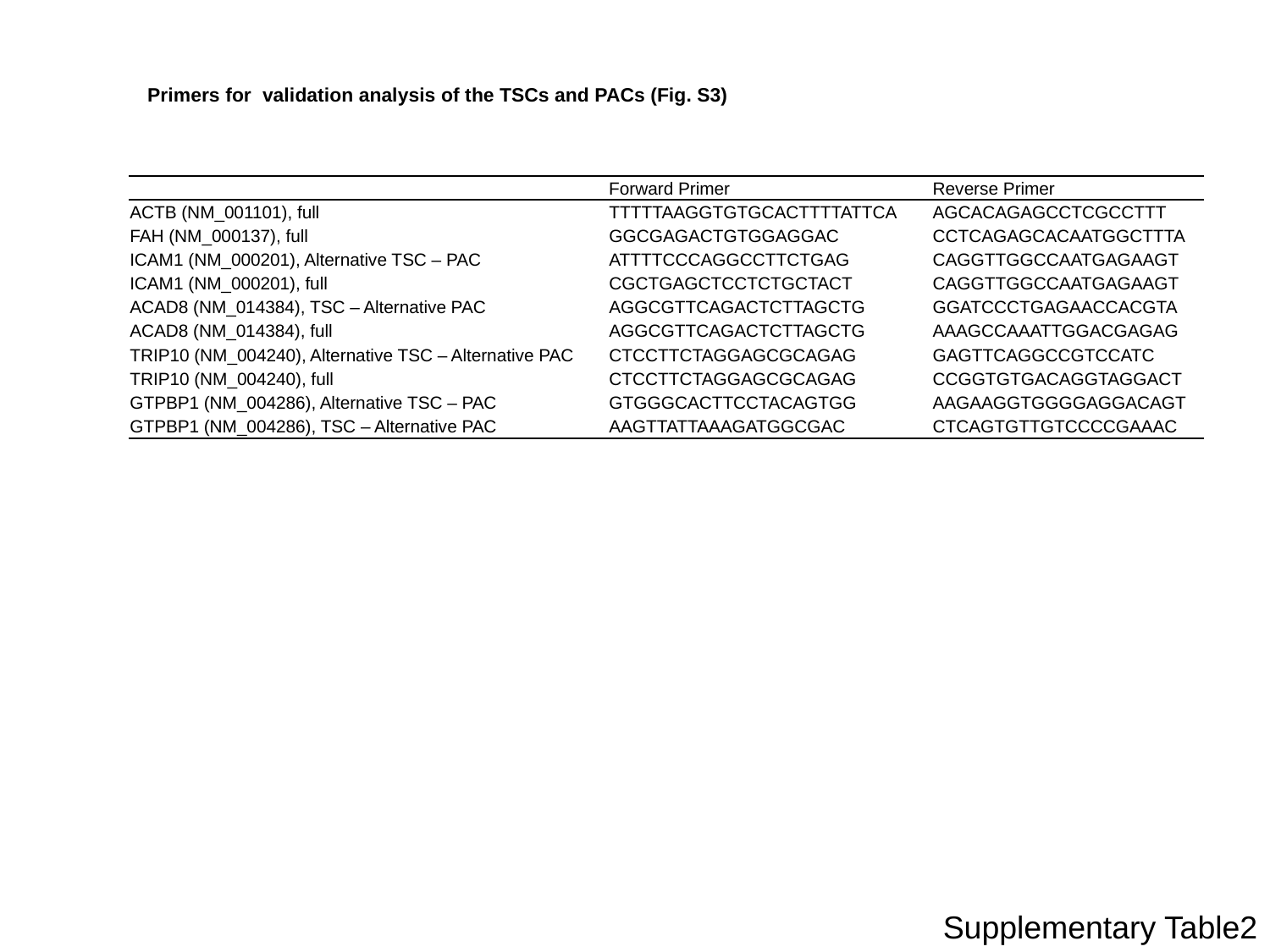

Primers for validation analysis of the TSCs and PACs (Fig. S3)
| | Forward Primer | Reverse Primer |
| --- | --- | --- |
| ACTB (NM\_001101), full | TTTTTAAGGTGTGCACTTTTATTCA | AGCACAGAGCCTCGCCTTT |
| FAH (NM\_000137), full | GGCGAGACTGTGGAGGAC | CCTCAGAGCACAATGGCTTTA |
| ICAM1 (NM\_000201), Alternative TSC – PAC | ATTTTCCCAGGCCTTCTGAG | CAGGTTGGCCAATGAGAAGT |
| ICAM1 (NM\_000201), full | CGCTGAGCTCCTCTGCTACT | CAGGTTGGCCAATGAGAAGT |
| ACAD8 (NM\_014384), TSC – Alternative PAC | AGGCGTTCAGACTCTTAGCTG | GGATCCCTGAGAACCACGTA |
| ACAD8 (NM\_014384), full | AGGCGTTCAGACTCTTAGCTG | AAAGCCAAATTGGACGAGAG |
| TRIP10 (NM\_004240), Alternative TSC – Alternative PAC | CTCCTTCTAGGAGCGCAGAG | GAGTTCAGGCCGTCCATC |
| TRIP10 (NM\_004240), full | CTCCTTCTAGGAGCGCAGAG | CCGGTGTGACAGGTAGGACT |
| GTPBP1 (NM\_004286), Alternative TSC – PAC | GTGGGCACTTCCTACAGTGG | AAGAAGGTGGGGAGGACAGT |
| GTPBP1 (NM\_004286), TSC – Alternative PAC | AAGTTATTAAAGATGGCGAC | CTCAGTGTTGTCCCCGAAAC |
Supplementary Table2

## Slide 21
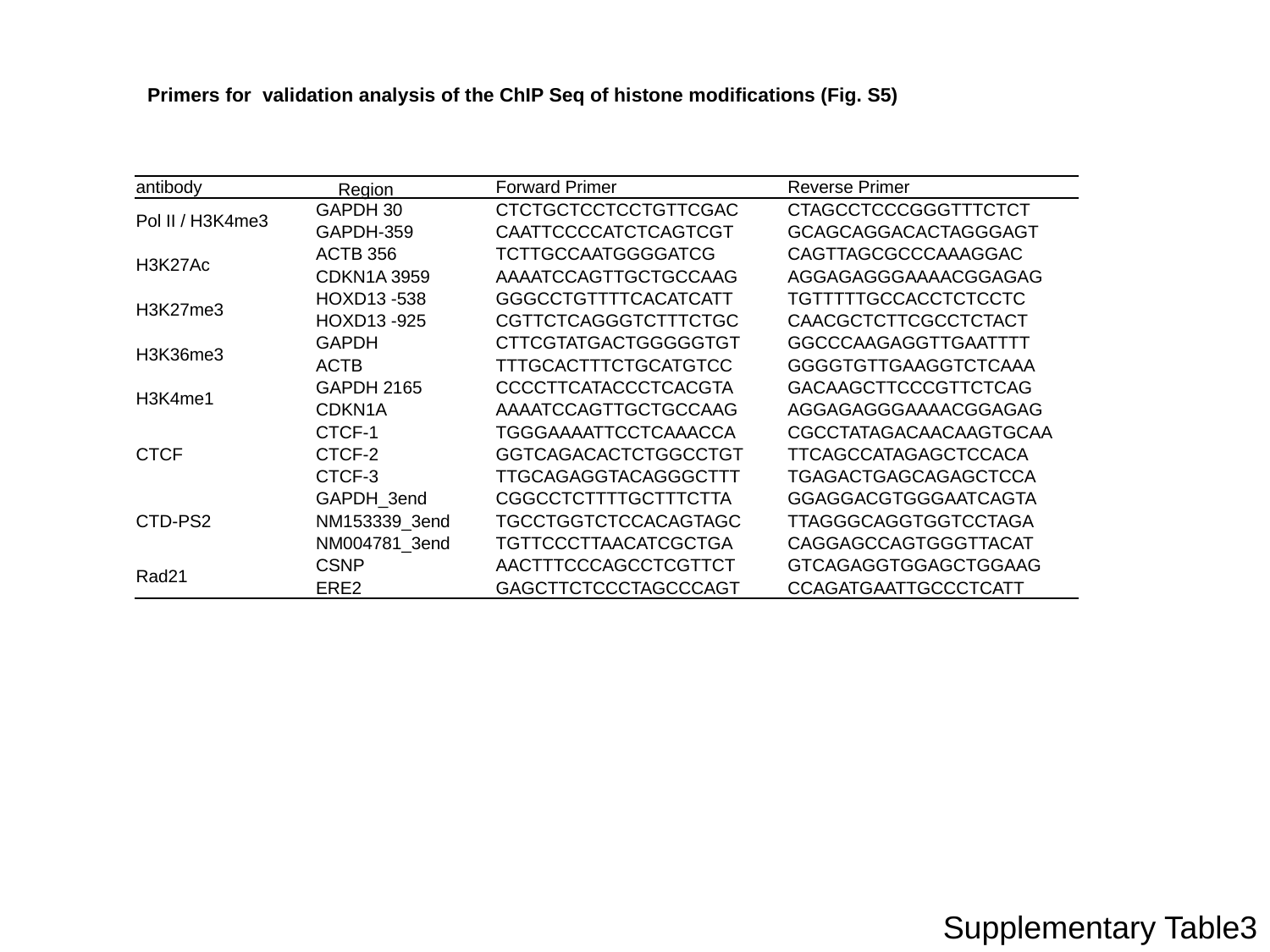

Primers for validation analysis of the ChIP Seq of histone modifications (Fig. S5)
| antibody | Region | Forward Primer | Reverse Primer |
| --- | --- | --- | --- |
| Pol II / H3K4me3 | GAPDH 30 | CTCTGCTCCTCCTGTTCGAC | CTAGCCTCCCGGGTTTCTCT |
| | GAPDH-359 | CAATTCCCCATCTCAGTCGT | GCAGCAGGACACTAGGGAGT |
| H3K27Ac | ACTB 356 | TCTTGCCAATGGGGATCG | CAGTTAGCGCCCAAAGGAC |
| | CDKN1A 3959 | AAAATCCAGTTGCTGCCAAG | AGGAGAGGGAAAACGGAGAG |
| H3K27me3 | HOXD13 -538 | GGGCCTGTTTTCACATCATT | TGTTTTTGCCACCTCTCCTC |
| | HOXD13 -925 | CGTTCTCAGGGTCTTTCTGC | CAACGCTCTTCGCCTCTACT |
| H3K36me3 | GAPDH | CTTCGTATGACTGGGGGTGT | GGCCCAAGAGGTTGAATTTT |
| | ACTB | TTTGCACTTTCTGCATGTCC | GGGGTGTTGAAGGTCTCAAA |
| H3K4me1 | GAPDH 2165 | CCCCTTCATACCCTCACGTA | GACAAGCTTCCCGTTCTCAG |
| | CDKN1A | AAAATCCAGTTGCTGCCAAG | AGGAGAGGGAAAACGGAGAG |
| CTCF | CTCF-1 | TGGGAAAATTCCTCAAACCA | CGCCTATAGACAACAAGTGCAA |
| | CTCF-2 | GGTCAGACACTCTGGCCTGT | TTCAGCCATAGAGCTCCACA |
| | CTCF-3 | TTGCAGAGGTACAGGGCTTT | TGAGACTGAGCAGAGCTCCA |
| CTD-PS2 | GAPDH\_3end | CGGCCTCTTTTGCTTTCTTA | GGAGGACGTGGGAATCAGTA |
| | NM153339\_3end | TGCCTGGTCTCCACAGTAGC | TTAGGGCAGGTGGTCCTAGA |
| | NM004781\_3end | TGTTCCCTTAACATCGCTGA | CAGGAGCCAGTGGGTTACAT |
| Rad21 | CSNP | AACTTTCCCAGCCTCGTTCT | GTCAGAGGTGGAGCTGGAAG |
| | ERE2 | GAGCTTCTCCCTAGCCCAGT | CCAGATGAATTGCCCTCATT |
Supplementary Table3

## Slide 22
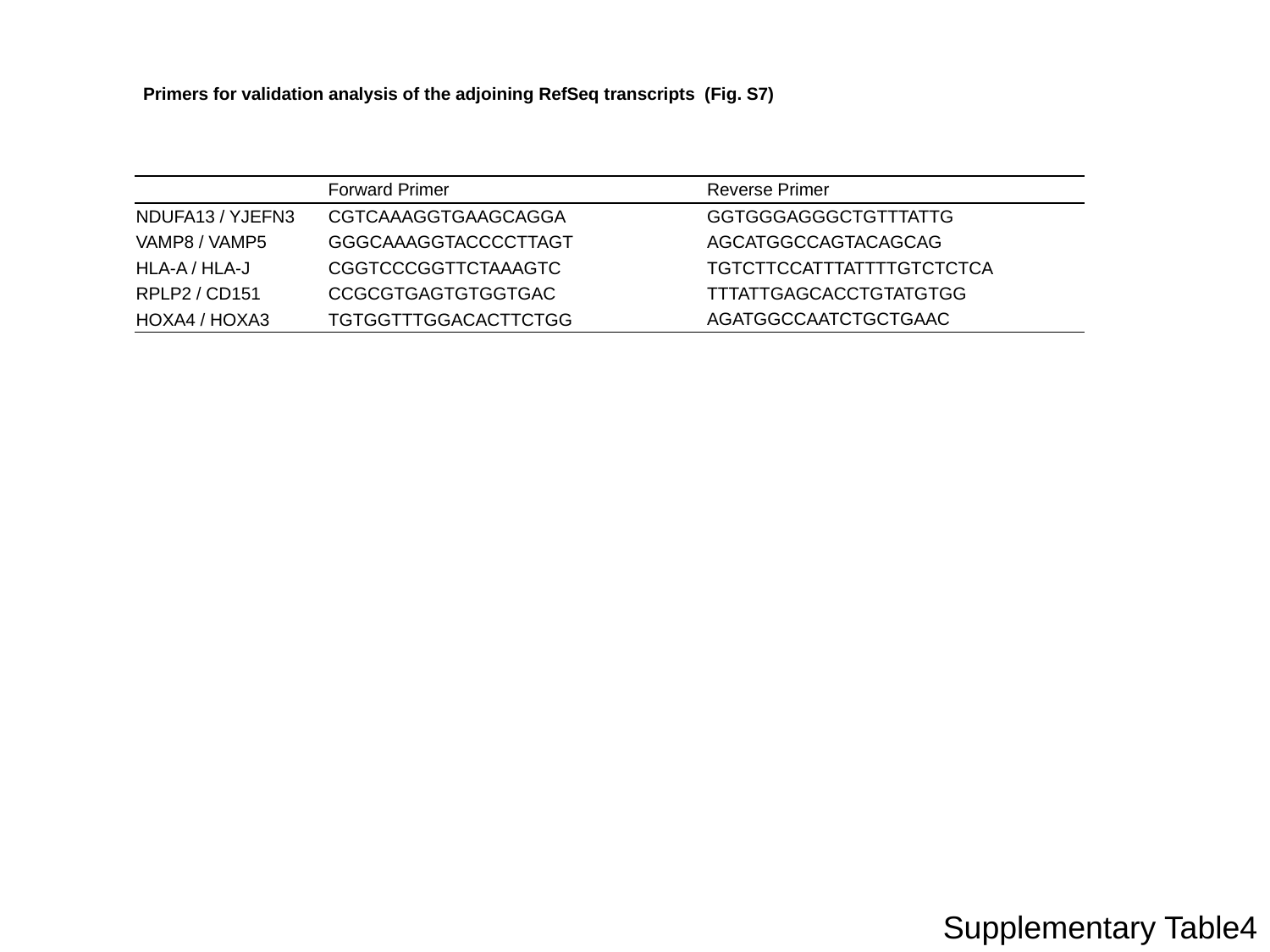

Primers for validation analysis of the adjoining RefSeq transcripts (Fig. S7)
| | Forward Primer | Reverse Primer |
| --- | --- | --- |
| NDUFA13 / YJEFN3 | CGTCAAAGGTGAAGCAGGA | GGTGGGAGGGCTGTTTATTG |
| VAMP8 / VAMP5 | GGGCAAAGGTACCCCTTAGT | AGCATGGCCAGTACAGCAG |
| HLA-A / HLA-J | CGGTCCCGGTTCTAAAGTC | TGTCTTCCATTTATTTTGTCTCTCA |
| RPLP2 / CD151 | CCGCGTGAGTGTGGTGAC | TTTATTGAGCACCTGTATGTGG |
| HOXA4 / HOXA3 | TGTGGTTTGGACACTTCTGG | AGATGGCCAATCTGCTGAAC |
Supplementary Table4

## Slide 23
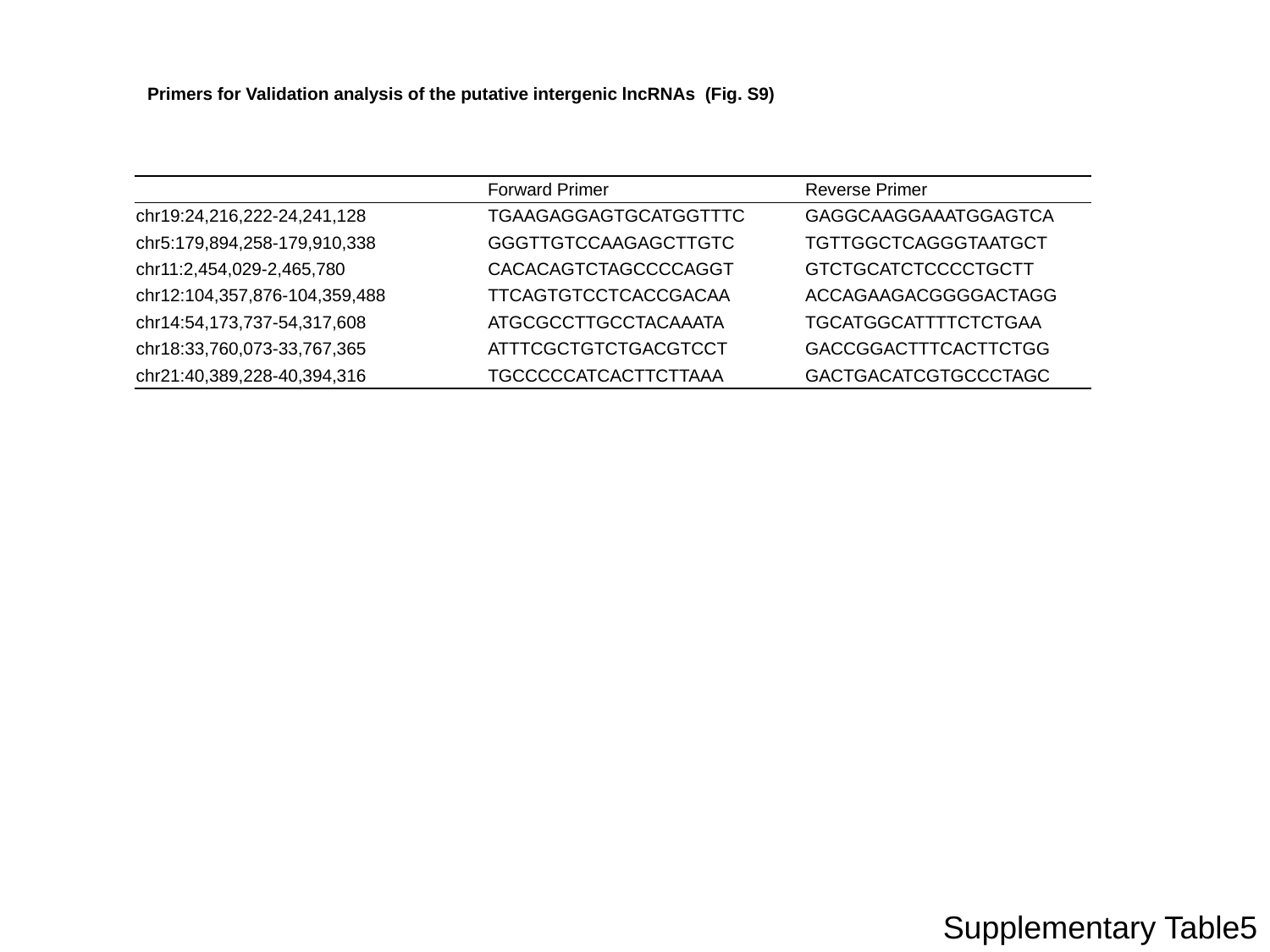

Primers for Validation analysis of the putative intergenic lncRNAs (Fig. S9)
| | Forward Primer | Reverse Primer |
| --- | --- | --- |
| chr19:24,216,222-24,241,128 | TGAAGAGGAGTGCATGGTTTC | GAGGCAAGGAAATGGAGTCA |
| chr5:179,894,258-179,910,338 | GGGTTGTCCAAGAGCTTGTC | TGTTGGCTCAGGGTAATGCT |
| chr11:2,454,029-2,465,780 | CACACAGTCTAGCCCCAGGT | GTCTGCATCTCCCCTGCTT |
| chr12:104,357,876-104,359,488 | TTCAGTGTCCTCACCGACAA | ACCAGAAGACGGGGACTAGG |
| chr14:54,173,737-54,317,608 | ATGCGCCTTGCCTACAAATA | TGCATGGCATTTTCTCTGAA |
| chr18:33,760,073-33,767,365 | ATTTCGCTGTCTGACGTCCT | GACCGGACTTTCACTTCTGG |
| chr21:40,389,228-40,394,316 | TGCCCCCATCACTTCTTAAA | GACTGACATCGTGCCCTAGC |
Supplementary Table5
