# Supplementary material for: Construction of mate pair full-length cDNAs libraries and characterization of transcriptional start sites and termination sites
Source: Nucleic Acids Res. 2014 Jul 17;42(16):e125. doi: 10.1093/nar/gku600 (PMC4176323; doi:10.1093/nar/gku600)
Supplement: SUPPLEMENTARY DATA [file supp_gku600_nar-00343-met-g-2014-File004.docx]

**SUPPLEMENTARY TABLE AND FIGURE LEGENDS**

Supplementary Figure S1. Statistics of the tags used for this study.

(A) Qualities of the starting RNA materials. RNA Integrity number for each sample is shown. Bioanalyzer charts for each samples are also shown. (B-D) Sequence tags generated and used for the analysis from (B) TSS-PAS libraries, (C) TSS-Random libraries and (D) ChIP Seq analysis. For details on the precise replacement of the cap structure with the synthetic oligo, see references (3), (25).

Supplementary Figure S2. Overlap of the TSCs identified in this study with TSCs from previous studies.

(A) Populations of the TSCs and PACs identified in this study that overlapped RefSeq (NM for protein-coding genes; NR for putative lncRNAs) alternative promoters (A), RefSeq alternative termination sites (B) and DBTSS (http://dbtss.hgc.jp/; C). (D) Overlap of the TSCs (upper) and PACs (lower) of >5 ppm that were identified in this study and the ones identified in a previous study using the RNA-PET method (23). (E) Comparison of the TSS tags and PAS tags identified in this study (Hs: humans) and in a previous study in *Drosophila melanogaster* (Dm) (22). To pair human and fly genes, Homologene (http://www.ncbi.nlm.nih.gov/homologene) was used. Pairs of genes for which sequence alignment of p< 1e-15 was obtained by LALIGN (https://www.ebi.ac.uk/Tools/psa/lalign/) for the exon regions were used. Statistics of the tags used for this analysis are shown in the upper panels. For the 1,541 such gene pairs, the numbers of tags in humans and flies (the seventh and the eighth columns) and the genomic coordinates at which those tags were mapped (the fifth and the sixth columns) were counted for the TSS tags and PAS tags (bottom panel; the fourth column). Genomic coordinates were counted separately depending on whether the sequence was aligned between humans and flies or the tags were present on both the human and fly sides (the first to the third columns). A fifty-base allowance was made to consider the presence of the tags in the different species. In the top margin, the tubulin gene is used as an example of how the tags were distributed. (F) Overlap of the PACs of >5 ppm that were identified in the present study with the ones identified by a previous study using the PA Seq method (11). (G) Presence of poly(A) addition signal patterns detected in the proximal regions of the PACs in this study (left panel). Note that the patterns are similar to the patterns identified by a previous study (right panel; (11)). (H) The TSS cDNA library was constructed using cap-replacing oligos with a random mixture of A/C/G/T at the 3’-end. The base composition of the donor site (the last base of the TSS oligo) and the adaptor site (the first base of the RNA) were separately counted for the different cap-replacing oligos (upper). Examples of TSS detection by different cap-replacing oligos (having the donor base as indicated by the vertical bars) at the indicated genomic positions are shown for the GAPDH gene (lower). (I) Length distribution of the first intron. Note that 8% of the first introns are longer than 50 kb. An example is shown of a case where the RefSeq model annotates a distal 5’-end, as well as a proximal 5’-end for an alternative promoter. Note that the downstream TSC was counted as downstream of the RefSeq 5’-end in this case, as we used the most distal 5’-end for the reference. (J) Details of Figure 2B. Distributions of the distances between the TSCs and the 5'-end of the RefSeq gene models (upper two panels) and those between the PACs and the 3'-end of the RefSeq genes models (lower two panels) are shown. Coarser bin sizes were used for the second and the fourth panels than those for the top and the third panels, so that the right most bar in the top panel and the left most bar in the third panel are further dissected. Examples of the cases are shown in the right margin for the cases where the TSCs or the PACs were separated from the termini of the RefSeq gene models by large distances.

Supplementary Figure S3. Validation analysis of TSCs and PACs

TSCs and PACs selected for validation analysis. The results of RT-PCR validation are shown for the indicated genes. Graphical views of the TSCs and PACs are also shown. The positions of the PCR primers are indicated in the chart by arrows (black arrows for alternative sites and white arrows for full-length transcripts).

Supplementary Figure S4. Examples of the alternative TSC or alternative PAC products

(A) Graphical view of examples of alternative promoter (left) and alternative termination sites (right) that showed tissue-preferred expression patterns. (B) (C) Presence of TATA box (left panel; B), CpG island (right panel; B) and poly(A)-additional signal (C) in the “preferred” TSC-PAC pairs.

Supplementary Figure S5. ChIP Seq analysis of the chromatin signatures in the surrounding regions of the TSCs and PACs

(A) (B) Results of similar analysis as conducted in Fig. 6 in different cell types for the TSC and PACs (A) and preferred TSC and PAC pairs (B). Note that essentially the same results were obtained regardless of cell type.

Supplementary Figure S6. Identification of the fusion gene transcripts in cancerous cell lines

(A) Models assumed for the fusion gene transcripts. (B) The numbers of cases in which the indicated categories of the gene fusions (right two cases in (A)) are suggested are shown. Also note that no chimeric transcript was identified by this selection procedure and thus was not originally included in the dataset. (C) List of the genes for fusion gene candidates. The numbers of tags representing fusion gene transcripts are shown for the indicated cell lines. Note the cases were identified using the filter of: Mapping Quality>=37; both the TSC and PAC should be located in the genic regions of RefSeq genes; tags should represent >5% of the total tags that correspond to the authentic intragenic TSC-PAC pair.

Supplementary Figure S7. Validation analysis of the adjoining RefSeq transcripts

TSCs and PACs selected for validation analysis. The results of the RT-PCR validations are shown for the indicated genes. Graphical views of the TSCs and PACs are also shown. The positions of the PCR primers are indicated in the chart by arrows.

Supplementary Figure S8. Schematic representation of the mapped assembly of transcript based on the TSS-Random sequence tags.

(A) Each arrow represents a tag obtained from the PAS or Random tags. The target genomic region which ranged from TSC to PAS and, thus, in which the transcript should be embedded is shown by the dotted lines. Tophat2 was used to detect splice junctions that were not represented in the RefSeq models. The integrity of the assembly was evaluated as the covered genomic regions compared to the target region. Note that potential intronic regions, which are represented by curved lines, are regarded as “covered” regions. (B) Number of cases where Tophat2 detected novel splicing patterns by the indicated number of supporting tags (upper). Statistics on the splicing sites for which alternative splicing events were represented by the indicated numbers of tags (lower). Example of cases where alternative splicings were represented by the TSS-Random tags but not properly represented. The site of suspected alternative splicing was shown in the red box. Note that this alternative splicing and the exon structures were confused in the assembled transcript model.

Supplementary Figure S9. Validation analysis of putative intervening non-protein coding RNAs.

TSCs and PACs selected for validation analysis. The results of the RT-PCR validations are shown for the indicated genes. Graphical views of the TSCs and PACs are also shown. The positions of the PCR primers are indicated in the chart by arrows. Their sequences are shown at the bottom.

Supplementary Tables 1-5: PCR primers for validation analyses.

Sequences of the PCR primers used for the indicated analyses are shown.
