# Supplementary material for: Construction of mate pair full-length cDNAs libraries and characterization of transcriptional start sites and termination sites
Source: Nucleic Acids Res. 2014 Jul 17;42(16):e125. doi: 10.1093/nar/gku600 (PMC4176323; doi:10.1093/nar/gku600)
Supplement: SUPPLEMENTARY DATA [file supp_gku600_nar-00343-met-g-2014-File005.docx]

Supplementary Methods

**Construction of TSS/TTS Mate Pair library and TSS/Random Mate Pair library**

1. Purification of total RNA using RNeasy

1. Add 15 mL of RLT to the recovered 7×10^7^cells.
2. Add 15 mL of 70% (v/v) ethanol to the tube and mix well.
3. Apply the solution to the RNeasy column, and let it pass through by brief centrifugation at 3500 rpm at room temperature (RT). Discard the flow-through and repeat this step until the entire sample has been applied.
4. To wash the column, apply 15 mL of RW to the column and centrifuge briefly at 3500 rpm at rt. Discard the flow-through.
5. To further wash the column, apply 10 mL of RPE to the column, and centrifuge briefly at 3500 rpm at RT. Discard the flow-through, and repeat this step once more with a 5-min centrifugation.
6. Use a fresh tube to collect the eluate. To elute the sample, apply 1 mL of dH_2_O to the column, and let it stand for 1 min at RT. Centrifuge at 3500 rpm for 5 min at RT. Collect the eluate.
7. Check the RNA with a bioanalyzer.


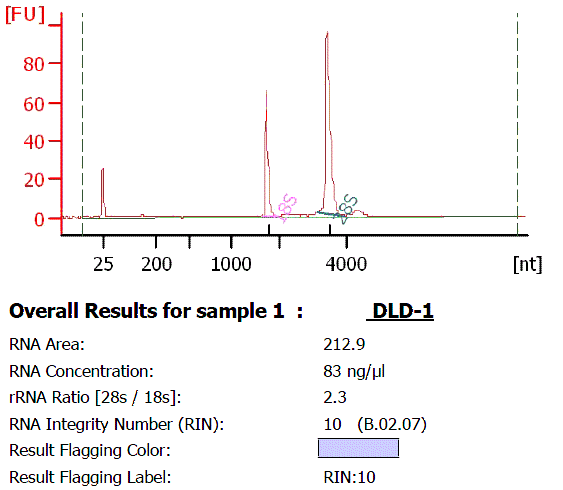


Bioanalyzer chart of total RNA

2. BAP Reaction

1. Dissolve 100 μg of the RNA in 100 μL of dH_2_O.
2. Incubate at 60 °C for 3 min to denature.
3. Set up a BAP reaction by combining:

sample 100 μL

dH_2_O 39.6 μL

5× BAP buffer 40.0 μL

RNasin 5.4 μL

BAP (0.4 U/μL) 12.5 μL

1. Incubate at 37 °C for 60 min.
2. Add an equal volume of phenol : chloroform (1 : 1) to the sample and mix well. Centrifuge at 14,000 rpm briefly at 4 °C. Transfer the upper aqueous layer to a fresh tube.
3. Ethanol precipitate the RNA by adding:

ethachinmate 1 μL

sodium acetate, pH 5.5 20 μL

100% ethanol 500 μL

Centrifuge at 14,000 rpm at 4 °C for 10 min.

1. Remove the supernatant and rinse the pellet with 100 μL of 80% (v/v) ethanol. Drying is not necessary.
2. Dissolve the sample in 36.65 μL of dH_2_O.

3. TAP Reaction

1. Set up a TAP reaction by combining:

sample 36.65 μL

5× TAP buffer 10.0 μL

RNasin 1.35 μL

TAP 2.0 μL

1. Incubate at 37 °C for 60 min.
2. Add 50 μL of dH_2_O.
3. Add an equal volume of phenol : chloroform (1 : 1) to the sample and mix well. Centrifuge at 14,000 rpm briefly at 4 °C. Transfer the upper aqueous layer to a fresh tube.
4. Ethanol precipitate the RNA by adding:

ethachinmate 1 μL

sodium acetate, pH 5.5 10 μL

100% ethanol 250 μL

1. Centrifuge at 14,000 rpm at 4 °C for 10 min. Remove the supernatant and rinse the pellet with 100 μL of 80% (v/v) ethanol. Drying is not necessary.
2. Re-suspend the sample in 21.0 μL of dH_2_O.

4. RNA Ligation

1. Ligate the BAP/TAP-treated poly(A)+ RNA to the 5’-oligoribonucleotide by combining:

sample 21.7 μL

5’-oligoribonucleotide (100 ng/μL) 12.0 μL

10× ligation buffer 30.0 μL

50 mM MgCl_2_ 60.0 μL

24 mM ATP 6.3 μL

RNasin 7.5 μL

T4 RNA ligase (40 U/μL) 12.5 μL

50% (w/v) PEG 8000 150.0 μL

1. Incubate at 20 °C for 3 h.
2. Add 450 μL of dH_2_O.
3. Extract with 300 μL of phenol : chloroform (1 : 1). Ethanol precipitate by adding 1 μL of ethachinmate, 60 μL of sodium acetate, pH 5.5 and 1500 μL of 100% ethanol (the rest of the procedure is as described in 3.6).
4. Dissolve the sample in 54.3 μL of dH_2_O.

5. DNase I Treatment

1. Remove the residual DNA with DNase I by combining:

sample 54.3 μL

25 mM MgCl_2_ 32.0 μL

1 M Tris-HCl (pH 7.0) 4.0 μL

0.1 M dithiothreitol (DTT) 5.0 μL

(*Use DTT supplied with SuperScript II)

RNasin 2.7 μL

DNase I 2.0 μL

1. Incubate at 37 °C for 10 min.
2. Extract with phenol : chloroform (1 : 1) and ethanol precipitate (as described in 3.4-6).
3. Dissolve the sample in 1200 μL of dH_2_O.

6. Poly(A) Selection of the RNA with Oligo-dT cellulose (Molecular Research Center)

1. Transfer the oligo-dT powder from two pre-packed columns* to a polypropylene Poly-Prep column (BIORAD). The bed volume of the powder should be approximately 0.5 mL when the powder from two columns is used.
2. Denature the dT powder by washing with 3 mL of 0.1 N NaOH.
3. Wash out the alkaline solution using 5 mL of dH_2_O.
4. Pre-equilibrate the column with 5 mL of 1× Loading Buffer.
5. Set a fresh tube to collect the flow-through.
6. Add an equal volume (1.2 mL) of 2× Loading Buffer to the sample, mix well and apply to the column.
7. Collect the flow-through and apply to the column. Repeat this step two more times.
8. Wash the column with 5 mL of 1× Loading Buffer.
9. Set a fresh collection tube and elute the sample by applying 3 mL of dH_2_O.
10. Add 8 mL of 100% ethanol and 330 μL of 3 M sodium acetate (pH 5.5), and centrifuge for ethanol precipitation.
11. Dissolve the sample in 100 μL of dH_2_O and ethanol precipitate once more (as described in 3.5-6).
12. Dissolve the sample in 21.0 μL of dH_2_O.

7. First Strand cDNA Synthesis

1. Synthesize first strand cDNA with reverse-transcriptase, SuperScript II, by combining:

sample 21.0 μL

5× First strand buffer 10.0 μL

4 dNTPs at 5 mM each 8.0 μL

0.1 M DTT 6.0 μL

oligo dT adapter primer* 2.5 μL

RNasin 1.0 μL

SuperScript II 2.0 μL

for the TSS/TTS Mate Pair library. For the TSS/Random Mate Pair library, use 2.5 μL of the dR (random hexamer) adapter primer instead of the oligo dT adapter primer.

* It may be better to utilize a different oligonucleotide that harbors a V (mix of C,G,A) at the 3’ terminal position. Such an oligonucleotide would exclude amplification initiated within long 3’ terminal poly(A) stretches, although we have not tried this option.

1. Incubate at 42 °C for more than 3 h for the TSS/TTS Mate Pair library. For the TSS/Random Mate Pair library, incubate at 12 °C for 1 h and 42 °C for more than 3 h. Note: The random hexamer oligonucleotide terminates in cytosine at the 3’ end, as we intended to enhance the hybridization at the starting site of the reverse transcription because the binding of the G-C pair is stronger than the binding of the A-T pair.
2. Add 50 μL of dH_2_O and extract the solution with phenol : chloroform (1 : 1) (as described in 3.4).
3. Add 2 μL of 0.5 M EDTA pH 8.0, to thoroughly stop the reaction.

8. Alkaline Degradation of the Template mRNA

1. Degrade the template RNA by adding 15 μL of 0.1 M NaOH. Incubate at 65 °C for 40 min.
2. Add 20 μL of 1 M Tris-HCl, pH 7.0 to neutralize.
3. To remove the fragmented RNA, ethanol precipitate the first strand cDNA by adding:

ethachinmate 1 μL

7.5 M ammonium acetate 70 μL

ethanol 500 μL

(the rest of the procedure is described in 3.6).

1. Dissolve the sample in 31 μL of dH_2_O.
2. Check 1 µL of the DNA using a bioanalyzer.

9. PCR Amplification of the cDNA

- 1. Use 10 μL for the TSS/TTS Mate Pair library, 20 μL for the TSS/Random Mate Pair library

sample + dH_2_O 52.4 μL

3.3× Reaction buffer II 30.0 μL

4 dNTPs at 2.5 mM each 8.0 μL

25 mM magnesium acetate 4.4 μL

PCR 5’-primer 1.6 μL

PCR 3’-primer 1.6 μL

DNA polymerase 2.0 μL

- 1. Thermocycle for 20 cycles at 94 °C, 1 min; 58 °C, 1 min; 72 °C, 2 min.
  2. Extract with phenol : chloroform (1 : 1) and ethanol precipitate (as described in 2.3.4-6).
  3. Dissolve the sample in 10 μL of dH_2_O.


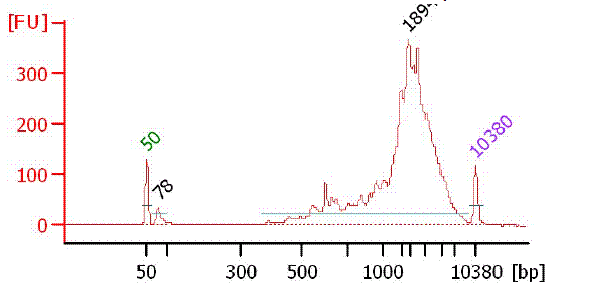


Bioanalyzer chart of PCR product

10. Size Fractionation of the PCR Products

1. Prepare a 1% agarose gel

2. Run gel at 100 V for 30 min.

3. Excise a chunk of gel containing the DNA fraction from 500 bp to 5 kbp for the TSS/TTS Mate Pair library. Excise three chunks of gel containing the DNA fractions from 500 bp to 1 kbp, from 1 kbp to 2 kbp, and from 2 kbp to 5 kbp for the TSS/Random Mate Pair library.

4. Purify the DNA using a Gel Extraction Kit.

5. Check the DNA using a bioanalyzer.


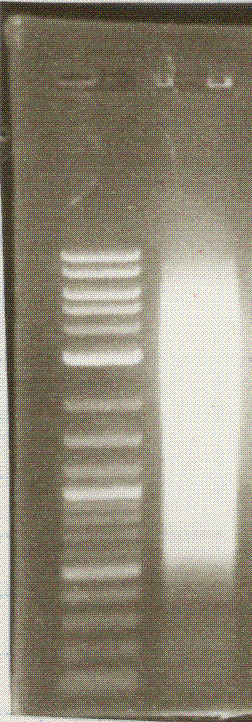


Picture of TSS/PAS PCR product on 1% agarose gel


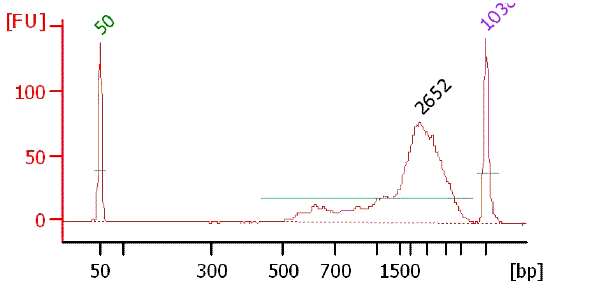


Bioanalyzer chart of

TSS/PAS gel fraction (0.5 - 5 kbp)

3

1

0.5


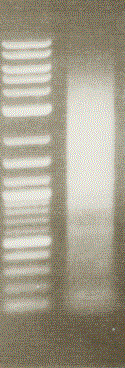


Picture of TSS/Random PCR product on 1% agarose gel

2k - 5k : fraction3

3

1

0.5

1k - 2k : fraction2

500 bp - 1k : fraction1


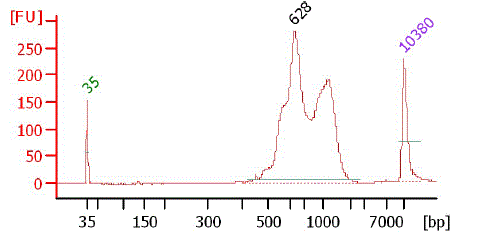


Bioanalyzer chart of

TSS/Random gel fraction (500 bp - 1 k)


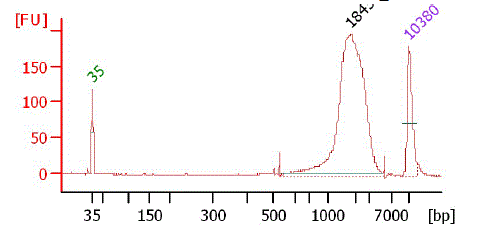


Bioanalyzer chart of

TSS/Random gel fraction (1 k - 2 k)


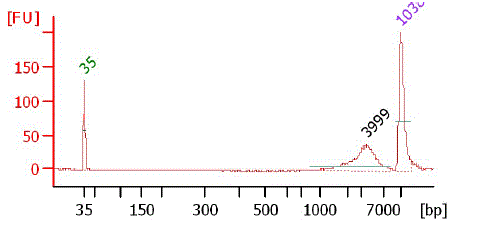


Bioanalyzer chart of

TSS/Random gel fraction (2 k - 5 k)

11. Perform End Repair

1. Dissolve 600 μg of the DNA in 75 μL of dH_2_O.

Size selected DNA 75 μL

10× End Repair Buffer 10 μL

Water 7.5 μL

Natural dNTP Mix 4 μL

T4 DNA Polymerase 5 μL

T4 Polynucleotide Kinase 5 μL

Klenow DNA Polymerase 1 μL

2. Incubate at 20 °C for 30 min. After incubation, place immediately on ice.

3. Purify the DNA using a QIAquick PCR Purification Kit. Dissolve the sample in 50 μL of dH_2_O.

12. Circularize DNA

1. Circularize the DNA to bind the TSS and TTS for the TSS/TTS Mate Pair library or to bind the TSS and inside cDNA for TSS/Random Mate Pair Library.

Sample 50 μL

10× circularization buffer 30 μL

dH_2_O 206.6 μL

Circularization ligase 13.4 μL

2. Incubate overnight for 16 h at 30 °C.

13. Digest Linear DNA

Remove the linear DNA using DNA exonuclease.

1. Add 3 μL of DNA exonuclease to 300 μL of the circularization reaction. Mix by gently flicking the tube and briefly centrifuge.

2. Incubate at 37 ºC for 20 minutes followed by 70 ºC for 30 minutes.

3. Add 12 μl of EDTA to the exonuclease-treated sample.

4. Add an equal volume of phenol : chloroform (1 : 1) to the sample and mix well. Centrifuge at 14,000 rpm briefly at 4 °C. Transfer the upper aqueous layer to a fresh tube.

5. Ethanol precipitate the RNA by adding:

ethachinmate 1 μL

3 M sodium acetate 30 μL

100 % ethanol 750 μL

6. Dissolve the sample in 37.5 μL of dH_2_O.

14. Fragment Circularized DNA

1. Fragment the circularized DNA at the *EcoP* 15I recognition site.

1. Sample 37.5 μl

10× NEBuffer 5 μL

100× BSA 0.5 μL

10× ATP 5 μL

*EcoP*15I 2 μL

2. Incubate at 37 °C for 1 h.

3. Inactivate the EcoP enzyme at 65 °C for 20 min.

4. Add 50 μL of dH_2_O.

5. Extract with phenol : chloroform (1 : 1) and ethanol precipitate (as described in 3.4-6).

6. Dissolve the sample in 50 μL of dH_2_O.

2. For the TSS/TTS Mate Pair library, additionally fragment with nebulization.

1. Transfer *EcoP*15I-treated DNA to the nebulizer, and add 250 μL of dH_2_O, 400 μL of the nebulization buffer and approximately 150 μL of 100% glycerol to the DNA and mix well.

2. Chill the nebulizer containing the DNA solution on ice while performing the next step.

3. Nebulize with delivered air at 32 psi for 6 min. Vapor may rise from the nebulizer, which is normal.

4. Centrifuge the nebulizer at 450 ×g for 2 min to collect approximately 400 μL of droplets from the side of the nebulizer.

5. Follow the instructions in the QIAquick PCR Purification Kit to purify the sample solution and concentrate it on one QIAquick column, eluting in 50 μl of dH_2_O.


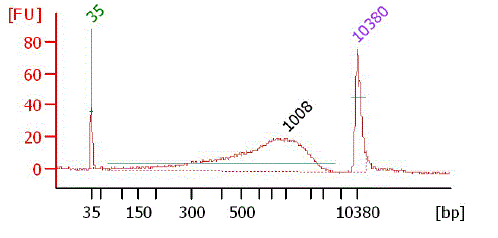


Bioanalyzer chart after nebulization

15. Purify Biotinylated DNA

Select the fragmented DNA with biotin using Dynal magnetic M-280

streptavidin beads.

1. Transfer 20 μL of well resuspended beads into a 1.5-mL microcentrifuge tube.

2. Place the tube in the magnetic rack for approximately 1 min, until the beads are separated from the solution. Remove and discard the supernatant.

3. Wash the beads. Add 50 μl of streptavidin bead binding buffer. Add the buffer solution, and resuspend the beads by gently flicking the tube. Centrifuge the tube for 1–2 s. Place the tube in the magnetic rack for 1 min, then remove and discard the supernatant.

4. Repeat step 3 once.

5. Resuspend the beads in 50 μL of fresh streptavidin bead binding buffer.

6. Add 50 μL of the bead solution to 50 μl of the fragmented DNA sample and incubate for 15 min at 20 ºC. Resuspend the beads every 2 min by gentle mixing.

7. Place the tube in the magnetic rack for 1 min, and then remove and discard the supernatant.

8. Wash the beads in 200 μL of streptavidin bead wash buffer 1. Add the buffer solution, and resuspend the beads by gently flicking the tube. Centrifuge the tube for 1–2 s. Place the tube in the magnetic rack for 1 min, then remove and discard the supernatant.

9. Repeat step 8 three times.

10. Wash the beads in 200 μL of QIAGEN EB Buffer. Add the buffer solution and resuspend the beads by gently flicking the tube. Centrifuge the tube for 1–2 s. Place the tube in the magnetic rack for 1 min, and then remove and discard the supernatant.

11. Wash the beads a second time in 200 μL of QIAGEN EB buffer. Place the sample on ice without removing the final wash solution.

16. Perform End Repair

Convert the overhangs resulting from fragmentation into blunt ends.

1. Prepare the reaction mix in a new tube on ice.

10× End Repair Buffer 10 μL

dH_2_O 75 μL

Natural dNTP Mix 4 μL

T4 DNA Polymerase 5 μL

T4 Polynucleotide Kinase 5 μL

Klenow DNA Polymerase 1 μL

1. Place the washed beads back on the magnet for 1 min; remove and discard the supernatant.
2. Resuspend the beads immediately in 100 μL of the end-repair reaction mix.
3. Incubate for 30 min at 20 ºC.
4. Place the tube in the magnetic rack for 1 min; remove and discard the supernatant.
5. Wash the beads in 200 μL of streptavidin bead wash buffer 1, as described in 15.8. and 15.9.
6. Wash the beads in 200 μL of QIAGEN EB buffer as described in 15.10.
7. Wash the beads a second time in 200 μL of QIAGEN EB buffer. Place the sample on ice without removing the final wash solution.

17. A-tail DNA Fragment

Add an ‘A’ base to the 3'-ends of the blunt phosphorylated DNA fragments.

1. Prepare the reaction mix in a new tube.

10× A-Tailing buffer 5 μL

dH_2_O 32 μL

1 mM dATP 10 μL

A-Tailing enzyme 3 μL

1. Place the washed beads back on the magnet for 1 min; remove and discard the supernatant.
2. Resuspend the beads immediately in 50 μL of A-Tailing reaction mix.
3. Incubate for 30 min at 37 °C.
4. Place the tube in the magnetic rack for 1 min; remove and discard the supernatant.
5. Wash the beads in 200 μL of streptavidin bead wash buffer 1 as described in 15.8. and 15.9.
6. Wash the beads in 200 μL of QIAGEN EB buffer as described in 15.10.
7. Wash the beads a second time in 200 μL of QIAGEN EB buffer. Place the sample on ice without removing the final wash solution.

18. Ligate Adapters to DNA Fragments

1. Prepare the reaction mix in a new tube.

2× Adapter Ligation Buffer 25 μL

dH_2_O 19 μL

PE Adapter Oligo Mix 1 μL

2. Place the washed beads back on the magnet for 1 min; remove and discard the supernatant.

3. Resuspend the beads immediately in 45 μL of A-Tailing reaction mix.

4. Add 5 μL of adapter ligase to the reaction, and mix by pipetting gently up and down several times.

5. Incubate for 15 min at 20 °C.

6. Wash the beads in 200 μL of streptavidin bead wash buffer 1 as described in 15.8. and 15.9.

7. Wash the beads in 200 μL of streptavidin bead wash buffer 2. Add the buffer solution, and resuspend the beads by gently flicking the tube. Centrifuge the tube for 1-2 s. Place the tube in the magnetic rack for 1 min, then remove and discard the supernatant. Repeat wash once, discarding wash supernatant.

8. Wash the beads in 200 μL of QIAGEN EB buffer as described in 15.10.

9. Wash the beads a second time in 200 μL of QIAGEN EB buffer. Place the sample on ice without removing the final wash solution.

19. Enrich Adapter-Modified DNA Fragments by PCR

1. Prepare the following PCR mix.

Phusion DNA polymerase 25 μL

dH_2_O 23 μL

PCR Primer 1.0 1 μL

PCR Primer 2.0 1 μL

2. Place the washed beads back on the magnet for 1 min; remove and discard the supernatant.

3. Resuspend the beads in 50 μL of the PCR mix and transfer to a 0.2 mL- PCR tube.

4. Thermocycle for 18 cycles at 98 °C for 10 s; 65 °C for 10 s; 72 °C for 30 s.

5. Remove and retain the PCR supernatant from the beads using a magnetic rack, and discard the beads.


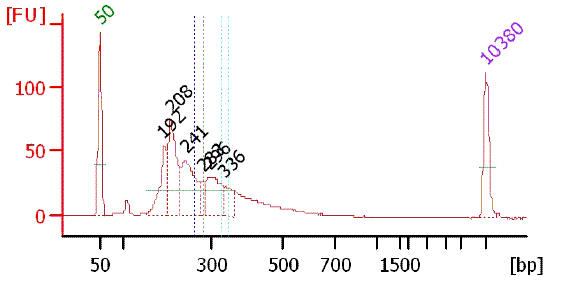


Bioanalyzer chart of PCR product

20. Size selection

Size Fractionation of the Products

1. Prepare an 8% polyacrylamide gel.
2. Run gel at 160 V for 90 min.
3. Excise a chunk of gel containing the DNA fraction from 280 bp to 330 bp for TSS/TTS Mate Pair library, and a single band at approximately 250 bp for TSS/Random Mate Pair library.
4. Purify the DNA and dissolve in 5 μL of dH_2_O.
5. Check 1 μL of the DNA with a bioanalyzer.


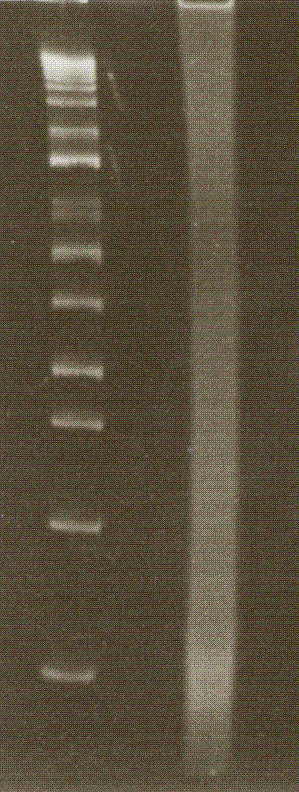


Picture of TSS/PAS PCR product on 8 % polyacrylamide gel

280 - 330bp

300

200


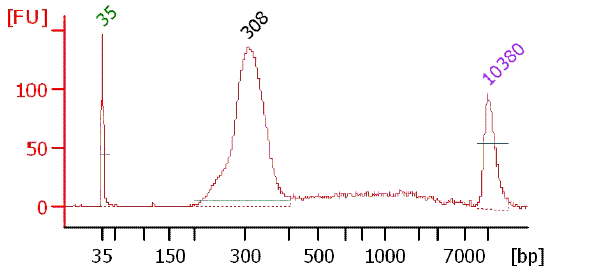


Bioanalyzer chart of purified TSS/PAS Mate Pair library


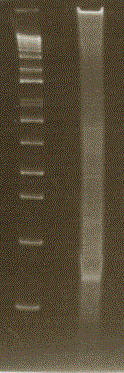


Picture of TSS/Random PCR product on 8 % polyacrylamide gel

300

200

250bp


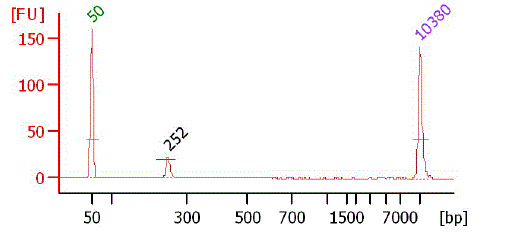

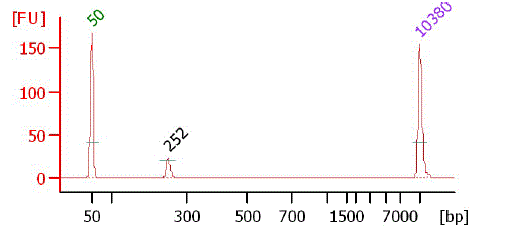

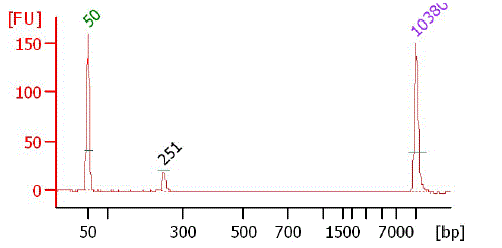


Bioanalyzer chart of

purified TSS/Random Mate Pair library (500 bp-1 k)

Bioanalyzer chart of

purified TSS/Random Mate Pair library (1 k-2 k)

Bioanalyzer chart of

purified TSS/Random Mate Pair library (2 k-5 k)

**ChIP-Seq**

1. Crosslinking

1. Use 1×10^8^ cells for ChIP seq.

2. Add a final concentration of 1% formaldehyde at room temperature for 10 min.

3. Add a final concentration of 166 mM glycine at room temperature for 10 min.

4. Wash the cells twice with 1×PBS.

5. Harvest the cells and centrifuge at 1,500 rpm for 5 min at 4 °C. Cells can be stored at -80 °C.

2. Prepare the magnetic beads

1. Translate 100 μL of proteinA magnetic beads or proteinG magnetic beads, depending on the antibody.

2. Add 1 mL of inhibition buffer and mix well gently.

3. Wash the beads with 1 mL of inhibition buffer. Place the washed beads back on the magnet for 1 min; remove and discard the supernatant.

4. Repeat step 3 three times.

5. Add 250 μL of inhibition buffer and 10 μg of antibody.

6. Rotate the beads with antibody for more than 4 h or over night at 4 °C.

7. Repeat step 3 and 4.

8. Dissolve the beads in 100 μL of inhibition buffer.

3. Lyse the cells and sonicate the cross-linked DNA

1. Add protease inhibitor to all lysis buffers before use.

2. Lyse the cells in 5 mL of Lysis Buffer 1.

3. Incubate the lysates at 4 °C for 10 min while gently flicking. Centrifuge at 1,500 rpm for 5 min at 4 °C. Discard the supernatant.

4. Resuspend the pellets in 5 mL of Lysis Buffer 2 and incubate at room temperature for 10 min; centrifuge at 1,500 rpm for 5 min at 4 °C. Discard the supernatant.

5. Resuspend the pellets in 1 mL of Lysis Buffer 3.

6. Sonicate the cells for 16 cycles of 30 s each on ice.

7. Add 100 μL of 10% Triton-X 100 to the sonicated cells.

8. Centrifuge at 14,000 rpm for 10 min.

9. Take the supernatant; save 50 μL of the supernatant for the controls (whole cell extract (WCE) DNA) at -20 °C.

4. Chromatin immunoprecipitation

1. Add 100 μL of beads with antibody prepared in step 2.8. in approximately 1 mL of lysate prepared in step 3.9.

2. Rotate the lysate over night at 4 °C.

5. De-crosslink

1. Wash the sample 8 times with 1 mL of wash buffer and once with TE buffer containing 50 mM NaCl, using the magnetic stand on ice.

2. Centrifuge at 1,000 rpm for 3 min at 4 °C. Discard the TE buffer.

3. Add 200 μL of the elution buffer to the beads.

4. Centrifuge at 14,000 rpm for 1 min at room temperature.

5. Take the 200 μl of supernatant.

6. Incubate at 65 °C for more than 6 h or over-night and de-crosslink (ChIP-DNA).

7. Melt the WCE DNA and add 150 μL of the elution buffer. Incubate at 65 °C for more than 6 h or over-night.

6. Resolution of protein and RNA

1. Add 200 μL of TE and 8 μL of 10 mg/ml RNase A to the ChIP-DNA and WCE DNA samples.

2. Incubate at 37 °C for 2 h.

3. Add 4 μL of 20 mg/ml proteinase K and 7 μL of 300 mM CaCl_2_.

4. Incubate at 55 °C for 30 min.

5. Purify using phenol/chloroform and ethanol precipitation. Elute samples with 35 μL of dH_2_O.


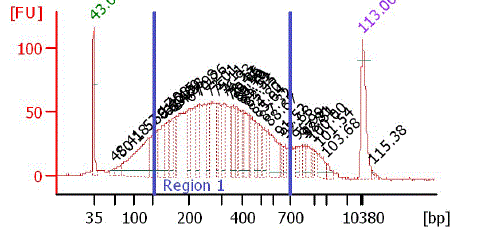


Bioanalyzer chart of eluted ChIP-DNA


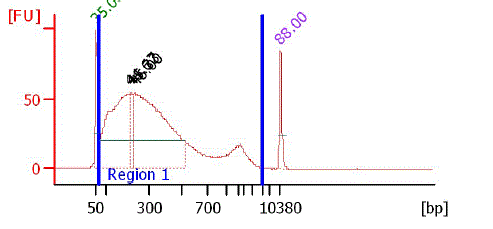


Bioanalyzer chart of eluted WCE-DNA

7. Preparation of ChIP library

1. Samples for the ChIP library were prepared according to the Illumina Truseq DNA preparation kit manufacturer’s instructions.


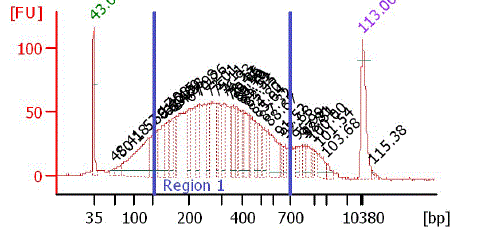


Bioanalyzer chart of prepared ChIP library


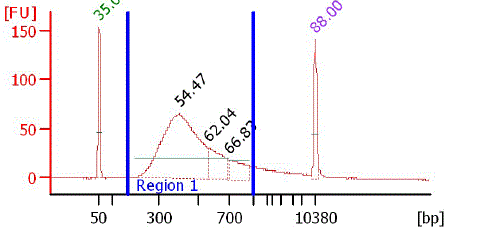


Bioanalyzer chart of prepared ChIP library (WCE)
